# Serine protease inhibitor from *Trichinella spiralis* ameliorates diet-induced obesity and adipose tissue inflammation through TIM-3-dependent macrophage reprogramming

**DOI:** 10.1371/journal.ppat.1014606

**Published:** 2026-09-25

**Authors:** Mingwei Tong, Ge Liu, Guidong Ren, Wangxuan Zhang, Yuyu Qiao, Xiaodan Yang, Weiping Fan, Hui Yang, Yong Yang

**Affiliations:** 1 Department of Endocrinology, First Hospital of Shanxi Medical University, Taiyuan, Shanxi, China; 2 School of Basic Medical Sciences, Shanxi Medical University, Jinzhong, China; 3 Shanxi Key Laboratory of Metabolic-Immune Homeostasis and Drug Innovation, Shanxi Medical University, Jinzhong, China; 4 Key Laboratory of Cellular Physiology (Shanxi Medical University), Ministry of Education, and Shanxi Key Laboratory of Cellular Physiology, Taiyuan, China; 5 Department of Infectious Diseases, First Hospital of Shanxi Medical University, Taiyuan, Shanxi, China; University of Dundee, UNITED KINGDOM OF GREAT BRITAIN AND NORTHERN IRELAND

## Abstract

Pro-inflammatory M1 polarization of adipose tissue macrophages (ATMs) drives adipose inflammation and obesity, suggesting that reprogramming ATM polarization holds therapeutic promise. Parasitic helminths have co-evolved with hosts to induce immune tolerance via M2 polarization, making helminth-derived M2-inducing molecules a potential therapeutic strategy against metabolic disorders. This study aimed to develop a defined serine protease inhibitor derived from *Trichinella spiralis* (Ts-SPI) as a novel immunomodulatory candidate against diet-induced metabolic disorders. In a high-fat diet (HFD)-induced obese mouse model, recombinant Ts-SPI (rTs-SPI) attenuated obesity and adipose tissue inflammation, and this effect was associated with its ability to reprogram macrophage polarization. *In vitro* studies confirmed that rTs-SPI drives phenotypic changes in both RAW264.7 cells and bone marrow-derived macrophages (BMDMs). Notably, a free fatty acid (FFA)-stimulated inflammatory adipocyte model and adoptive transfer assays demonstrated that rTs-SPI-reprogrammed macrophages mediate the anti-obesity and anti-inflammatory effects. Mechanistically, TIM-3 was identified as a key mediator through *in vivo* TIM-3 blockade assays. Pharmacological inhibition revealed downstream PI3K/AKT/mTOR signaling. In summary, this study identifies a parasite-derived protein as a potent TIM-3-targeting biologic candidate for treating metabolic inflammation, proposes a novel “helminth-inspired checkpoint modulation” strategy, and provides new insight into parasite-host immune crosstalk.

## Author summary

Pro-inflammatory M1 polarization of adipose tissue macrophages (ATMs) serves as a key driver of metabolic inflammation. Repolarizing ATMs toward an anti-inflammatory M2 state represents a promising therapeutic strategy. Parasitic helminths naturally induce M2 responses to evade host immunity, suggesting they could serve as a natural source of therapeutic molecules. Here we demonstrate that a defined serine protease inhibitor derived from *Trichinella spiralis* (Ts-SPI) effectively counteracts obesity in high-fat diet-fed mice. rTs-SPI treatment attenuates adipose inflammation and reprograms ATMs from M1 to M2 phenotype. rTs-SPI-triggered immunomodulation was confirmed in RAW264.7 cells and bone marrow-derived macrophages. The functional role of rTs-SPI-reprogrammed macrophages was validated using a free fatty acid-stimulated adipocyte model and adoptive transfer assays. Mechanistically, *in vivo* immune checkpoint TIM-3 blockade identified TIM-3 as a key mediator, with downstream PI3K/AKT/mTOR signaling involved. This study identifies a parasite-derived biologic candidate, supports a novel “helminth-inspired checkpoint modulation” strategy for metabolic inflammation, and provides new insight into parasite-host immune crosstalk.

## Introduction

Obesity and its associated metabolic syndrome pose a formidable global health challenge,affecting approximately 13% of the adult population worldwide [1] and significantly increasing mortality rates from cancer and a variety of chronic diseases [2,3]. Chronic low-grade inflammation within expanding adipose tissue is now recognized as a central driver of insulin resistance and systemic metabolic dysfunction [4,5]. In this inflammatory microenvironment, the dynamic polarization of adipose tissue macrophages (ATMs) plays a pivotal role [6]. A hallmark of obesity is the phenotypic shift in ATMs from an anti-inflammatory M2 state toward a pro-inflammatory M1 state, a transition that exacerbates metabolic deterioration [7,8]. Therefore, therapeutic strategies aimed at reprogramming macrophage polarization and restoring immune homeostasis represent a promising approach, underscoring the urgent need for novel and effective immunomodulators.

The “hygiene hypothesis” and epidemiological observations have long suggested an inverse relationship between helminth infections and the incidence of metabolic disorders [9,10]. Animal models support the ability of helminth infections or certain components to prevent metabolic dysfunction, including insulin resistance, obesity, and nonalcoholic fatty liver disease (NAFLD) [11–13]. It is well recognized that parasitic helminths have co-evolved with hosts to establish immunological privilege and immune tolerance [14]. Anti-inflammatory M2 macrophages are critical for maintaining self-tolerance and immune homeostasis [15], and helminths are proposed to promote M2 polarization to attenuate the host’s immune attack and prolong survival [16]. This immunoregulation, which prevents excessive immune activation, concurrently protects the host against inflammatory disorders. For example, administration of *Schistosoma mansoni*-soluble egg antigens (SEA) induces M2 macrophages to prevent diet-induced obesity [17]. We have recently found that crude extracts from *T. spiralis* muscle larvae protect against high fat diet (HFD)-induced obesity and chronic inflammation, an effect associated with their capacity to regulate adipose tissue macrophages [18].

Although helminth-inspired therapies hold promise, drug development remains a challenge owing to the side effects of live helminth therapy and the undefined complexity of crude helminth products. The identification and characterization of helminth molecules and the molecular pathways they target in the host present a unique opportunity to develop nature-inspired novel drugs that are efficacious, safe, and minimally immunogenic [19]. Nevertheless, the identification of protective active helminth-derived molecules remains poorly characterized. Serine protease inhibitors from helminths protect the organisms from serine protease hydrolysis and facilitate immune evasion [20,21]. *T. spiralis* serine protease inhibitor (Ts-SPI) has recently been shown to exhibit potent anti-inflammatory regulatory effects and to play a crucial role in immune regulation during *T. spiralis* infection [22,23]. Specifically, Ts-SPI has been reported to alleviate experimental colitis in mice by promoting M2 macrophage polarization [24]. Our previous research also demonstrated that Ts-SPI ameliorates NAFLD in mice through its anti-inflammatory properties [25]. However, whether specific parasite-derived molecules, such as Ts-SPI, can directly mitigate HFD-induced obesity and adipose tissue inflammation, and whether their therapeutic effects are mediated by direct regulation of macrophage polarization, remain largely unexplored.

The immune checkpoint molecule T-cell immunoglobulin and mucin-domain containing-3 (TIM-3) has emerged as a key regulator of macrophage function, influencing their polarization and cytokine production beyond its established role in T cell exhaustion [26]. While the function of TIM-3 in cancer and autoimmunity is well-documented [27,28], and it has recently been implicated in nonalcoholic steatohepatitis (NASH) [29], its role in obesity-associated adipose tissue inflammation remains nascent and underexplored. Crucially, whether TIM-3 serves as a direct target for parasite-derived immunomodulators such as Ts-SPI remains unknown.

Herein, we identify Ts-SPI as a potential immunomodulator that attenuates HFD-induced obesity and adipose tissue inflammation by targeting TIM-3 on macrophages to promote their phenotypic shift from M1 to M2 polarization. Our findings reveal a novel Ts-SPI/TIM-3 axis in immunometabolism regulation and identify a promising biologic for developing innovative, parasite-inspired therapeutics for metabolic diseases.

## Results

### rTs-SPI alleviates HFD-induced obesity in mice

**Fig 1:**
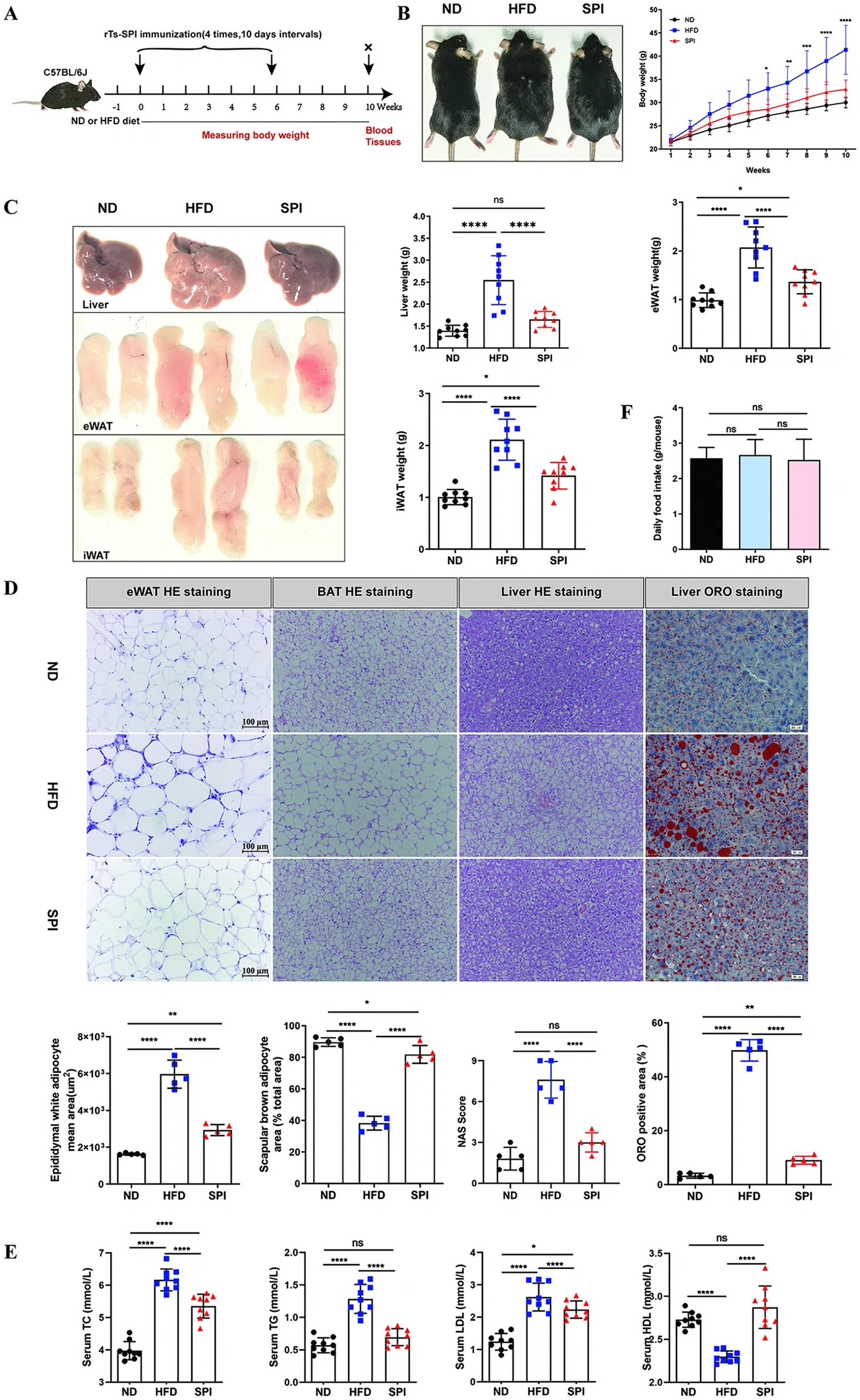
rTs-SPI alleviates HFD-induced obesity in mice. Mice were fed an HFD and administered rTs-SPI via intraperitoneal injection, and then samples were collected for analysis (n = 9 per group). (A) Experimental design timeline. (B) Gross appearance of representative mice and weekly body weight was recorded throughout the experiments. n = 9, biological replicates within a single experiment. ^*^p < 0.05, ^**^p < 0.01, ^***^p < 0.001, and ^****^p < 0.0001, HFD group compared with SPI group, two-way ANOVA followed by Sidak’s multiple comparison test. (C) Gross appearance and weights of liver, eWAT, and iWAT. n = 9, biological replicates within a single experiment. ^*^p < 0.05, ^***^p < 0.001, ^****^p < 0.0001, and ns means no statistical difference, one-way ANOVA with Tukey’s post-hoc test. (D) Photomicrographs of adipose tissue and liver sections after staining with H&E (scale bar: 100 μm) and ORO (scale bar: 20 μm). n = 5 biological replicates. ^*^p < 0.05, ^**^p < 0.01, ^****^p < 0.0001, and ns means no statistical difference, one-way ANOVA with Tukey’s post-hoc test. (E) Serum levels of TC, TG, LDL, and HDL were determined using an automated biochemical analyzer. n = 9, biological replicates within a single experiment. ^*^p < 0.05, ^****^p < 0.0001, and ns means no statistical difference, one-way ANOVA with Tukey’s post-hoc test. (F) Food intake was monitored every other day throughout the experiment, and daily food intake per mouse was calculated. ns means no statistical difference, one-way ANOVA with Tukey’s post-hoc test. Data are expressed as mean ± SD.

To investigate the effect of rTs-SPI on obesity, an obese mouse model was established by feeding a HFD for 10 weeks. Body weight (BW), along with the weights of the liver, epididymal white adipose tissue (eWAT), and inguinal white adipose tissue (iWAT) were measured. As expected, HFD feeding significantly increased BW, liver, eWAT, and iWAT weights (Fig 1B and C). H&E and Oil Red O (ORO) staining revealed excessive lipid accumulation in both adipocytes and hepatocytes induced by HFD (Fig 1D). In contrast, treatment with rTs-SPI significantly reduced the HFD-induced increases in body and tissue weights, as well as lipid deposition (Fig 1B-D). Moreover, rTs-SPI ameliorated HFD-induced hyperlipidemia, as indicated by a significant decrease in serum total cholesterol (TC), triacylglycerol (TG), and low-density lipoprotein cholesterol (LDL), and an increase in high-density lipoprotein cholesterol (HDL) after treatment (Fig 1E). Notably, there was no significant difference in food intake among the groups (Fig 1F). Collectively, these findings demonstrate that rTs-SPI has marked potential to alleviate HFD-induced obesity and associated metabolic disturbances, and its beneficial effects are not attributable to reduced food intake.

### rTs-SPI decreases adipose tissue inflammation in HFD-fed mice

**Fig 2:**
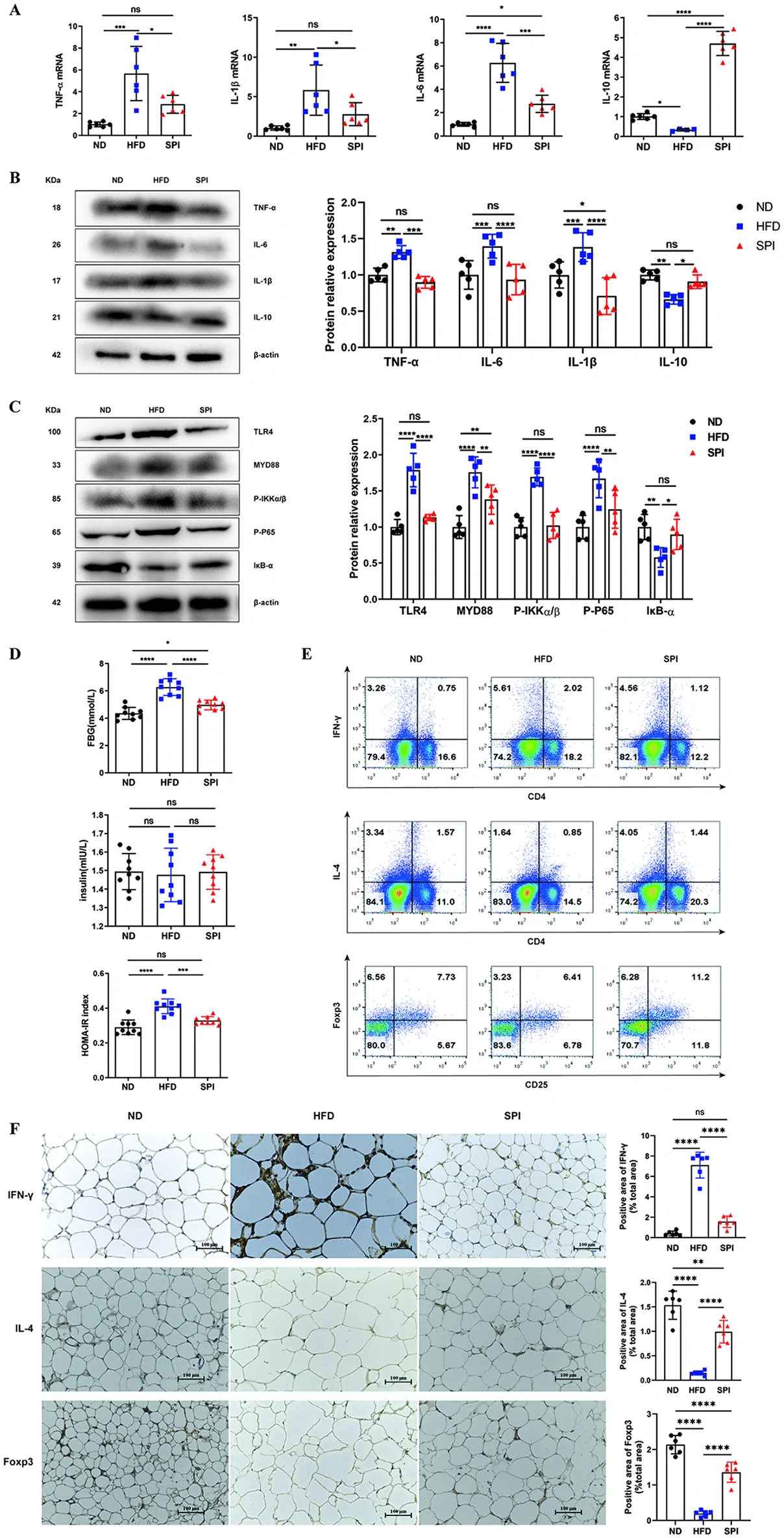
rTs-SPI decreases adipose tissue inflammation and alters CD4^+^ T cell subsets in HFD-fed mice. Mice were fed an HFD and administered rTs-SPI via intraperitoneal injection. Serum and eWAT were collected for analysis. (A) mRNA expression levels of the cytokines TNF-α, IL-1β, IL-6, and IL-10 in eWAT were analyzed by qRT-PCR. For IL-10, the HFD group comprised n = 4 after exclusion of two samples with Ct values > 35; all other groups had n = 6. n represents biological replicates. ^*^p < 0.05, ^**^p < 0.01, ^***^p < 0.001, and ^****^p < 0.0001, one-way ANOVA with Tukey’s post-hoc test. (B) Protein levels of cytokines TNF-α, IL-6, IL-1β, and IL-10 in eWAT were analyzed by Western blot. n = 5 biological replicates. ^*^p < 0.05, ^**^p < 0.01, ^***^p < 0.001, ^****^p < 0.0001, and ns means no statistical difference, one-way ANOVA with Tukey’s post-hoc test. (C) Protein levels of TLR4, MyD88, p-IKKα/β, p-P65, and IκB-α in eWAT were determined by Western blot. n = 5 biological replicates. ^*^p < 0.05, ^**^p < 0.01, ^****^p < 0.0001, and ns means no statistical difference, one-way ANOVA with Tukey’s post-hoc test. (D) Serum levels of FBG were determined using an automated biochemical analyzer. Plasma insulin was quantified using an ELISA kit. The insulin resistance index (HOMA-IR) was calculated using the standard formula. n = 9, biological replicates within a single experiment. ^***^p < 0.001, ^****^p < 0.0001, and ns means no statistical difference, one-way ANOVA with Tukey’s post-hoc test. (E) SVF was isolated from eWAT. Expression of IFN-γ, IL-4, and Foxp3 in CD4^+^ T cells within the SVF was analyzed by flow cytometry. (F) Expression of IFN-γ, IL-4, and Foxp3 in eWAT sections was analyzed by immunohistochemical analysis. n = 6 biological replicates. ^**^p < 0.01, ^****^p < 0.0001, and ns means no statistical difference, one-way ANOVA with Tukey’s post-hoc test. Data are expressed as mean ± SD.

Obesity is commonly accompanied by chronic inflammation [30]. To investigate the role of rTs-SPI in adipose tissue inflammation, we first measured the expression levels of inflammatory cytokines in eWAT. As shown in Fig 2A and B, compared with the ND group, HFD feeding significantly upregulated the mRNA and protein levels of cytokines TNF-α, IL-1β, and IL-6, while downregulating IL-10. Treatment with rTs-SPI partially reversed these alterations, bringing expression levels closer to those observed in the ND group. Notably, in the context of HFD-induced adipose tissue inflammation, the elevated IL-6 expression observed in our study is interpreted as a pro-inflammatory response, consistent with its well-documented role as a pro-inflammatory cytokine in obesity [31,32].

Within the TLR4/NF-κB pro-inflammatory pathway, rTs-SPI markedly reduced the protein levels of TLR4, MyD88, p-IKKα/β, and phosphorylated NF-κB (p-P65), while upregulating IκB-α expression in eWAT (Fig 2C). Given that enhanced activation of the NF-κB pathway is associated with insulin resistance, we further evaluated the effect of rTs-SPI on insulin sensitivity. As shown in Fig 2D, rTs-SPI decreased fasting blood glucose (FBG) and ameliorated insulin resistance in HFD-fed mice, although circulating insulin levels remained unchanged.

Flow cytometric analysis revealed that HFD feeding altered the proportions of adipose tissue T cell subsets; however, these changes were largely restored by rTs-SPI treatment. Specifically, HFD increased the proportion of CD4⁺ IFN-γ⁺ Th1 cells in eWAT, while decreasing the proportions of CD4⁺IL-4⁺ Th2 cells and CD4⁺CD25⁺Foxp3⁺ Treg cells. Administration of rTs-SPI suppressed the HFD-induced increase in Th1 cells and restored the diminished populations of Th2 and Treg cells (Fig 2E). To complement our flow cytometry data, we performed immunohistochemical staining for IFN-γ, IL-4, and Foxp3 on adipose tissue sections. Compared with the HFD group, eWAT sections from rTs-SPI-treated obese mice exhibited increased Foxp3⁺ and IL-4⁺ immunoreactivity, along with reduced IFN-γ⁺ cells (Fig 2F), which was broadly consistent with the trends observed by flow cytometry (Fig 2E). Collectively, these results demonstrate that rTs-SPI acts as an adipose tissue inflammation-suppressing protein, associated with suppression of the TLR4/NF-κB signaling pathway and attenuation of Th1 differentiation in HFD-fed mice.

### The effect of rTs-SPI is related to its immunoregulatory role in reprogramming macrophage polarization

**Fig 3:**
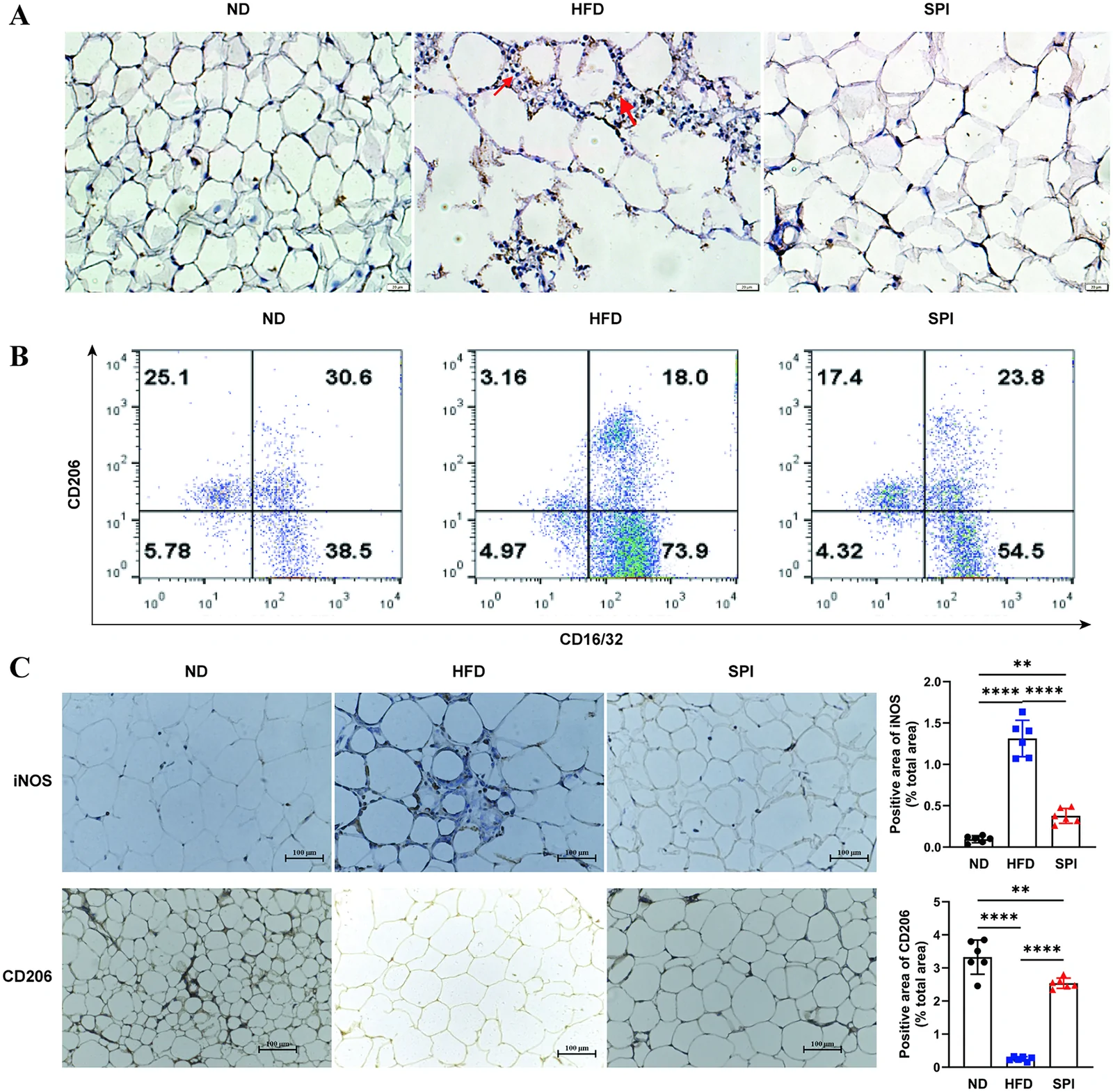
rTs-SPI regulates macrophage polarization in the SVF of HFD-fed mice. Mice were fed an HFD and administered rTs-SPI via intraperitoneal injection. eWAT were collected for analysis. (A) Immunohistochemical analysis of F4/80^+^ macrophages in eWAT sections. Red arrows indicate F4/80^+^ macrophages. (B) Flow cytometric analysis of M2 (CD16/32^-^CD206^+^) and M1 (CD16/32^+^CD206^-^) macrophages in SVF, gated on F4/80^+^ cells. (C) Immunohistochemical analysis of iNOS (M1 marker) and CD206 (M2 marker) in eWAT sections. n = 6 biological replicates. Data are expressed as mean ± SD, ^**^p < 0.01 and ^****^p < 0.0001, one-way ANOVA with Tukey’s post-hoc test.

Macrophage accumulation in adipose tissue and their phenotypic switching contributes to obesity-associated inflammation [33]. Given the anti-inflammation activity of rTs-SPI in the adipose tissue of HFD-fed mice, we next investigated whether this protein could modulate macrophage polarization. Immunohistochemistry (IHC) using the macrophage-specific antigen F4/80 as a marker revealed that HFD increased the number of F4/80^+^ macrophages, which encircled adipocytes to form crown-like structures (CLS). Administration of rTs-SPI inhibited this HFD-induced accumulation of F4/80⁺ macrophages (Fig 3A).

In addition to reducing total F4/80^+^ macrophages, rTs-SPI exhibited a robust immunoregulatory effect on macrophage polarization. Specifically, flow cytometric analysis showed that HFD almost doubled the frequency of pro-inflammatory M1 macrophages (F4/80^+^⁺CD16/32^+^⁺CD206^−^) in eWAT compared with the ND group (73.9% vs. 38.5%). Conversely, rTs-SPI decreased M1 macrophages and increased anti-inflammatory M2 macrophages (F4/80^+^⁺CD16/32^−^CD206^+^) compared with HFD-fed mice (Fig 3B). To corroborate these findings, we performed immunohistochemical staining for the M1 marker iNOS and the M2 marker CD206 on adipose tissue sections. Compared with the HFD group, eWAT sections from rTs-SPI-treated obese mice exhibited decreased iNOS immunoreactivity and increased CD206 immunoreactivity (Fig 3C), a pattern consistent with the flow cytometry results (Fig 3B). These findings indicate that rTs-SPI administration promotes resolution of obesity-related adipose tissue inflammation by reprogramming macrophage polarization.

### rTs-SPI induces the alternative activation of RAW264.7 and BMDMs *in vitro*

**Fig 4:**
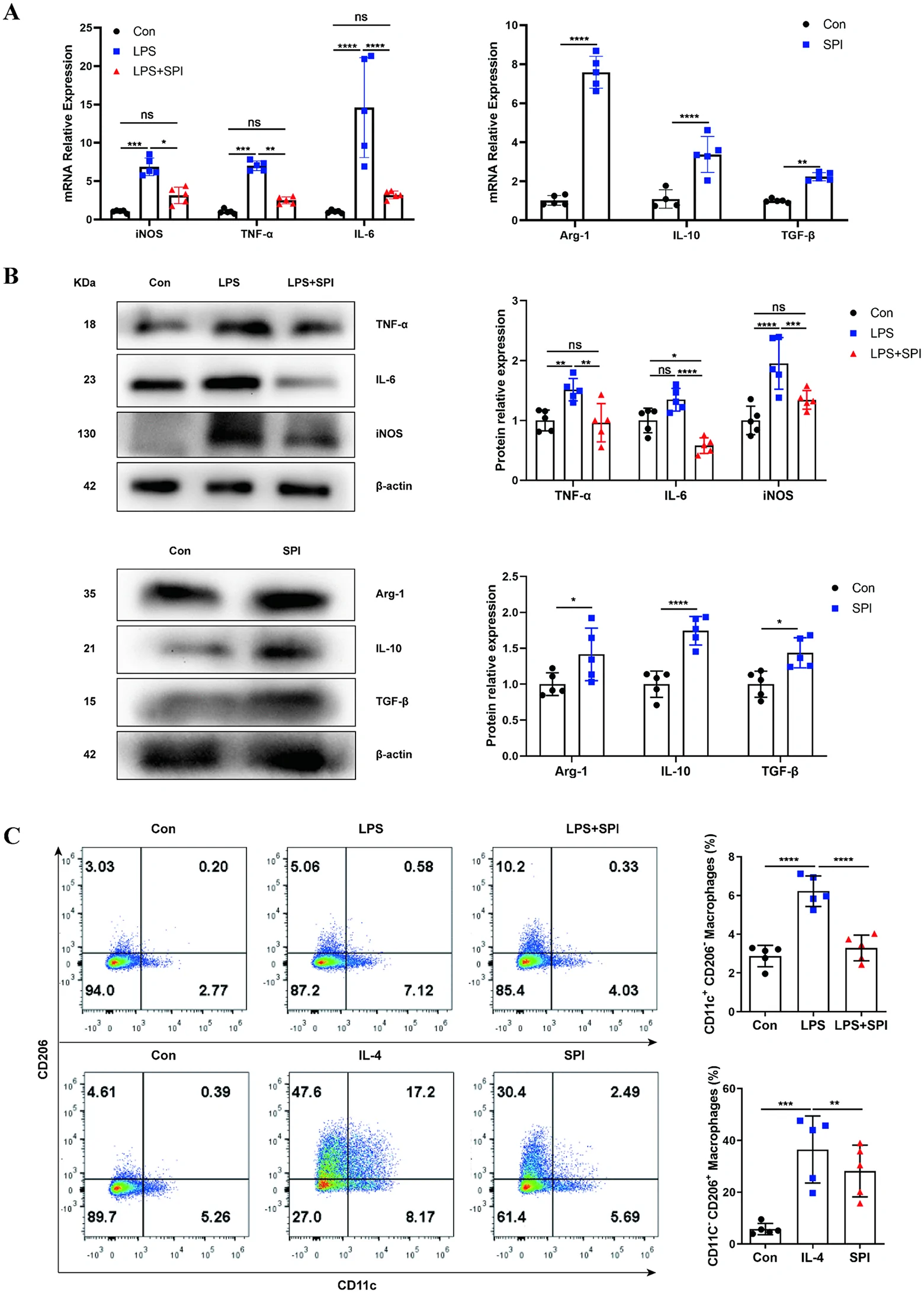
Immunoregulatory properties of rTs-SPI on RAW264.7 cells and BMDMs. RAW264.7 cells were incubated with rTs-SPI (10 µg/mL) alone or co-incubated with LPS (100 ng/mL) for 24 **h.** M1- and M2-related markers were assessed by qRT-PCR (A) and Western blot **(B)**. n = 5 biological replicates. For IL-10 in the qRT-PCR analysis, the Con group comprised n = 4 after exclusion of one sample with Ct values >35. ^*^p < 0.05, ^**^p < 0.01, ^***^p < 0.001, ^****^p < 0.0001, and ns means no statistical difference, two-way ANOVA with Sidak’s multiple comparison test. **(C)** BMDMs were incubated with rTs-SPI (10 µg/mL) alone or co-incubated with LPS (100 ng/mL) for 24 **h.** Cells challenged with LPS (100 ng/mL) or IL-4 (20 ng/mL) were used as positive controls. Flow cytometric analysis of M1 (F4/80^+^CD11c^+^CD206^-^) and M2 (F4/80^+^CD11c^-^CD206^+^) subsets in BMDMs. n = 5 biological replicates. ^**^p < 0.01, ^***^p < 0.001, and ^****^p < 0.0001, one-way ANOVA with Tukey’s post-hoc test. Data are expressed as mean ± SD.

To verify the immunoregulatory effects of rTs-SPI on macrophages, we performed *in vitro* validation experiments using RAW264.7 cells and bone marrow-derived macrophages (BMDMs). In RAW264.7 cells, HFD feeding increased the M1 population, as indicated by elevated expression of iNOS and the cytokines TNF-α and IL-6 at both the transcriptional and translational levels. However, rTs-SPI treatment significantly attenuated these increases (Fig 4A and B). Along with the reduction in M1 macrophages, rTs-SPI enhanced M2 polarization, as evidenced by the upregulation of Arg-1 and the cytokines IL-10 and TGF-β (Fig 4A and B).

This phenotypic switch triggered by rTs-SPI was further corroborated in BMDMs. Flow cytometric analysis confirmed more than 80% purity of BMDMs (F4/80⁺) prior to protein exposure (S1 Fig). Consistent with the *in vivo* data, rTs-SPI markedly decreased the proportion of F4/80^+^CD11c^+^CD206^-^ M1 macrophages, while increasing the F4/80^+^CD11c^-^CD206^+^ M2 macrophages (Fig 4C). Collectively, our *in vivo* and *in vitro* results consistently demonstrate that rTs-SPI drives macrophage polarization toward the alternatively activated, anti-inflammatory M2 phenotype.

### Medium of rTs-SPI-treated BMDMs decreases lipid accumulation and inflammation in 3T3-L1 adipocytes

**Fig 5:**
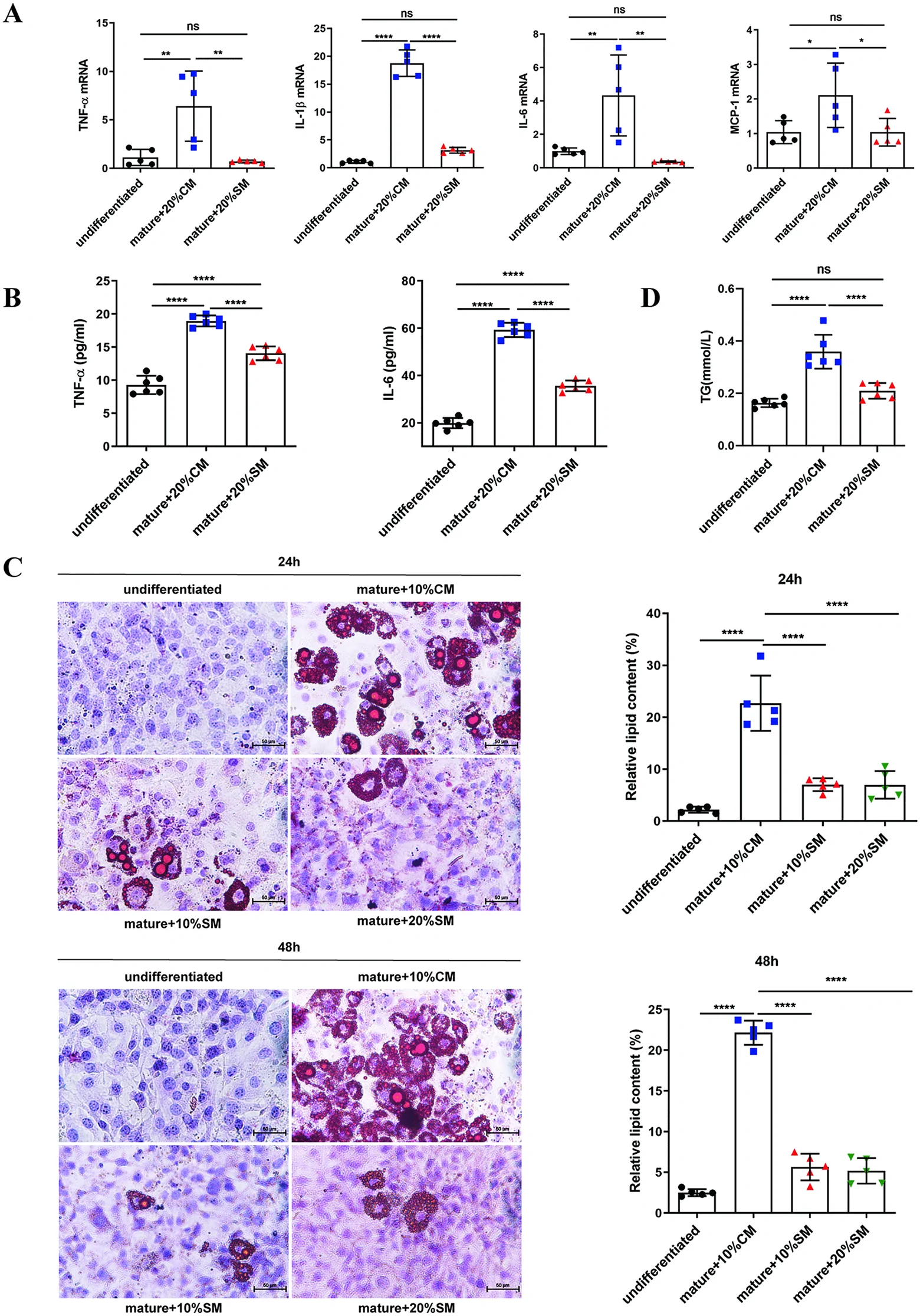
Conditioned medium from rTs-SPI-treated BMDMs decreases inflammation and lipid accumulation in 3T3-L1 mature adipocytes. 3T3-L1 preadipocytes were differentiated into mature adipocytes; thereafter, the mature adipocytes were challenged with FFA and co-cultured with conditioned medium from BMDMs that had been exposed to either rTs-SPI (SM) or PBS (CM). (A) qRT-PCR analysis of TNF-α, IL-1β, IL-6, and MCP-1 in 3T3-L1 adipocytes. n = 5 biological replicates. **(B)** ELISA analysis of TNF-α and IL-6 levels in the culture medium of 3T3-L1 adipocytes. n = 6 biological replicates. **(C)** Cellular lipid deposition in adipocytes treated with either SM or CM for 24 or 48 h (scale bar: 50 μm). n = 5 biological replicates. **(D)** TG levels in the culture medium of 3T3-L1 adipocytes. n = 6 biological replicates. ^*^p < 0.05, ^**^p < 0.01, ^***^p < 0.001, ^****^p < 0.0001, and ns means no statistical difference, one-way ANOVA with Tukey’s post-hoc test. Data are expressed as mean ± SD.

Given the beneficial effects of rTs-SPI on obesity and its associated adipose tissue inflammation, coupled with our demonstration that rTs-SPI polarizes macrophages toward an anti-inflammatory phenotype, we next investigated whether rTs-SPI-conditioned macrophages contribute to the alleviation of obesity. 3T3-L1 preadipocytes were differentiated to mature adipocytes and then challenged with FFA in the presence of conditioned medium from BMDMs that had been pre-exposed to either rTs-SPI (SM, rTs-SPI-conditioned medium) or vehicle (CM, PBS-conditioned medium), to mimic the obese adipose tissue microenvironment following rTs-SPI treatment. As shown in Fig 5A, compared with undifferentiated preadipocytes, FFA-stimulated mature adipocytes exhibited markedly elevated mRNA levels of the cytokines TNF-α, IL-1β, and IL-6, as well as the chemokine MCP-1. However, these increases were largely abolished when CM was replaced with SM. Concurrently, ELISA analysis of the culture supernatants revealed that SM significantly attenuated the FFA-induced secretion of TNF-α and IL-6 compared with CM (Fig 5B).

Excessive lipid accumulation is a well-recognized hallmark of adipocyte dysfunction in obesity [34]. Remarkably, adipocytes exposed to SM exhibited a pronounced reduction in lipid droplet content compared with those incubated with CM, an effect that was consistent across multiple SM dilutions and time points (24 h and 48 h) (Fig 5C). Similar to these findings, SM dramatically lowered the TG levels in the culture medium (Fig 5D). Overall, these data indicate that rTs-SPI-programmed macrophages attenuate FFA-elicited inflammation and lipid deposition in adipocytes.

### Adoptive transfer of rTs-SPI-treated BMDMs ameliorates HFD-induced obesity and adipose tissue inflammation in mice

**Fig 6:**
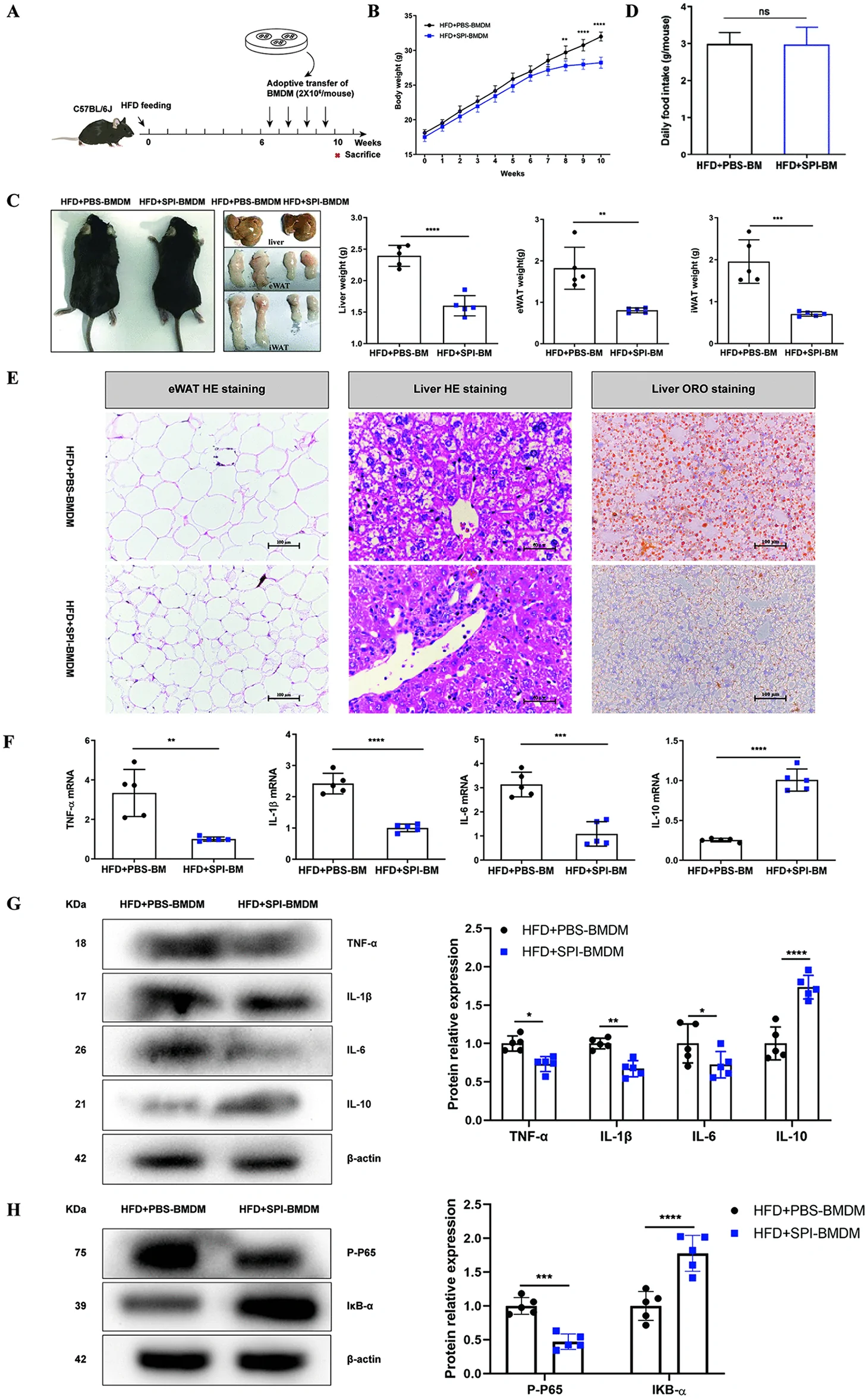
Adoptive transfer of rTs-SPI-treated BMDMs ameliorates HFD-induced obesity and adipose tissue inflammation in mice. Male C57BL/6J mice were maintained an HFD for 10 weeks. During weeks 7-10, PBS- or rTs-SPI-treated BMDMs (2x10^6^ cells per mouse) were adoptively transferred by intravenous injection into mice once weekly (total of 4 injections). **(A)** Adoptive transfer protocol. **(B)** Body weight changes in mice. n = 5, biological replicates within a single experiment. ^**^p < 0.01 and ^****^p < 0.0001, two-way ANOVA followed by Sidak’s multiple comparison test. **(C)** Gross appearance and weights of liver, eWAT, and iWAT. n = 5, biological replicates within a single experiment. ^**^p < 0.01, ^***^p < 0.001, and ^****^p < 0.0001, unpaired two-tailed Student’s t-test. **(D)** Food intake was monitored every other day throughout the experiment. Daily food intake per mouse was calculated. ns means no statistical difference, unpaired two-tailed Student’s t-test. **(E)** Photomicrographs of tissue sections after staining with H&E and ORO. Scale bar: 100 μm. (F) qRT-PCR and **(G)** Western blot analyses of the cytokines TNF-α, IL-1β, IL-6, and IL-10 in eWAT. n = 5 biological replicates. ^*^p < 0.05, ^**^p < 0.01, ^***^p < 0.001, and ^****^p < 0.0001, unpaired two-tailed Student’s t-test for (F) and two-way ANOVA followed by Sidak’ multiple comparison test for **(G)**. **(H)** Western blot detection of p-P65 and IκB-α in eWAT. n = 5 biological replicates. ^***^p < 0.001 and ^****^p < 0.0001, two-way ANOVA followed by Sidak’s multiple comparison test. Data are expressed as mean ± SD.

To further confirm the contribution of rTs-SPI-programmed macrophages to the mitigation of HFD-induced obesity and adipose tissue inflammation, we performed adoptive transfer experiments (Fig 6A). Consistent with our *in vitro* observations, mice that received rTs-SPI-treated BMDMs exhibited a marked attenuation of HFD-associated metabolic and inflammatory phenotypes. Specifically, recipients of rTs-SPI-treated BMDMs displayed reduced body weight and lower liver, eWAT, and iWAT weights compared with mice given PBS-treated BMDMs (Fig 6B and C), whereas food intake remained unchanged (Fig 6D). Histological analysis revealed diminished lipid accumulation in both adipocytes and hepatocytes (Fig 6E). Additionally, the levels of the cytokines TNF-α, IL-1β, and IL-6 were suppressed, while the anti-inflammatory cytokine IL-10 was upregulated (Fig 6F and G). We also observed a similar decrease in NF-κB phosphorylation and an increase in its inhibitory molecule IκB-α (Fig 6H). Collectively, these *in vivo* data corroborate our *in vitro* adipocyte findings and confirm that rTs-SPI drives macrophage polarization toward an anti-inflammatory phenotype, thereby alleviating HFD-induced obesity and adipose tissue inflammation.

### Blocking TIM-3 diminishes the beneficial effects of rTs-SPI in HFD-fed mice

**Fig 7:**
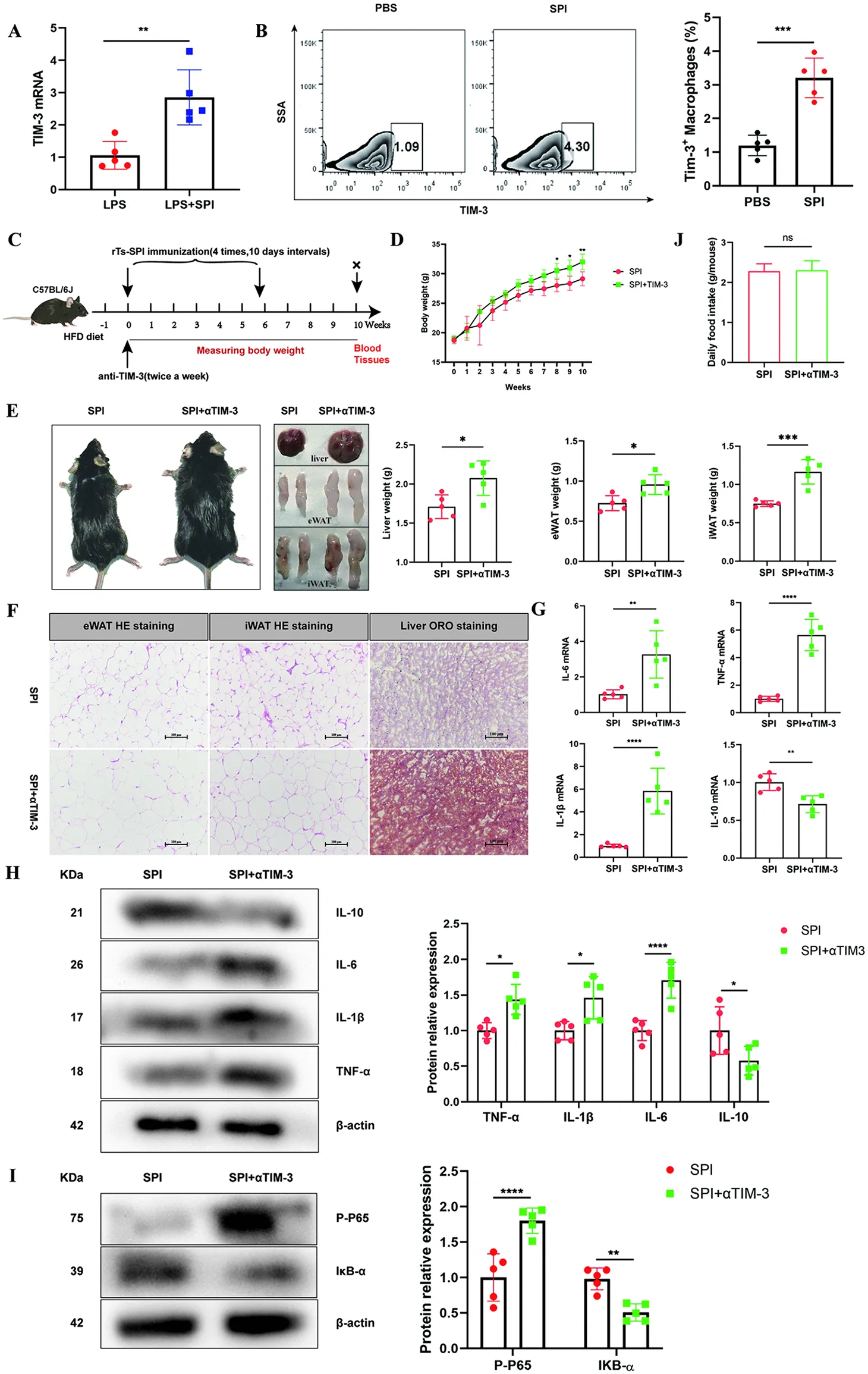
TIM-3 blockade abates rTs-SPI-mediated protection against obesity and adipose tissue inflammation in HFD-fed mice. **(A)** BMDMs were treated with LPS (100 ng/mL) alone or co-incubated with rTs-SPI (10 µg/mL) for 24 h. qRT-PCR analysis of TIM-3 mRNA in BMDMs. n = 5 biological replicates. ^**^p < 0.01, unpaired two-tailed Student’s t-test. **(B)** BMDMs were treated with or without rTs-SPI (10 µg/mL) for 24 **h.** Flow cytometric analysis of TIM-3 expression in BMDMs, gated on F4/80^+^ cells. n = 5 biological replicates. ^***^p < 0.001, unpaired two-tailed Student’s t-test. **(C-J)** Male C57BL/6J mice were maintained an HFD for 10 weeks. Beginning at week 0, each mouse received 200 µg of anti-TIM-3 monoclonal antibody or an equivalent dose of isotype control mAb (rat IgG2a) via intraperitoneal injection twice weekly throughout the experimental period. One day after the initial antibody treatment, all mice were treated intraperitoneally with rTs-SPI for four doses at 10-day intervals. **(C)** Experimental protocol. **(D)** Body weight changes in mice. n = 5 biological replicates within a single experiment. ^*^p < 0.05 and ^**^p < 0.01, two-way ANOVA with Sidak’s multiple comparison test. **(E)** Gross appearance and weights of liver, eWAT, and iWAT. n = 5 biological replicates within a single experiment. ^**^p < 0.01 and ^***^p < 0.001, unpaired two-tailed Student’s t-test. **(F)** Photomicrographs of tissue sections after staining with H&E and ORO. Scale bar: 100 μm. (G) qRT-PCR and **(H)** Western blot analyses of the cytokines TNF-α, IL-1β, IL-6, and IL-10 in eWAT. n = 5. Experiments were repeated for at least 3 times. ^*^p < 0.05, ^**^p < 0.01, and ^****^p < 0.0001, unpaired two-tailed Student’s t-test for (G) and two-way ANOVA followed by Sidak’s multiple comparison test for **(H)**. **(I)** Western blot analysis of P-P65 and IκB-α in eWAT. n = 5. Experiments were repeated for at least 3 times. ^**^p < 0.01 and ^****^p < 0.0001, two-way ANOVA followed by Sidak’s multiple comparison test. **(J)** Food intake was monitored every other day throughout the experiment. Daily food intake per mouse was calculated. ns means no statistical difference, unpaired two-tailed Student’s t-test. Data are expressed as mean ± SD.

We have shown that rTs-SPI drives macrophage polarization toward an anti-inflammatory phenotype. Given that TIM-3 has been implicated in metabolic disorders, such as nonalcoholic steatohepatitis and insulin resistance [29,35], and that it regulates macrophage polarization in sepsis [36], we hypothesized that TIM-3 might serve as a molecular mediator through which rTs-SPI regulates macrophage repolarization and confers protection against HFD-induced obesity. We first examined whether rTs-SPI affects TIM-3 expression in BMDMs, and found that TIM-3 was significantly upregulated upon rTs-SPI treatment in both LPS-stimulated and unstimulated BMDMs (Fig 7A and B). To functionally validate this finding, we performed an *in vivo* TIM-3 blocking experiment (Fig 7C).

Obese mice receiving rTs-SPI were randomized to treatment with anti-TIM-3 neutralizing antibody or an isotype control. Neutralization of TIM-3 significantly reversed the rTs-SPI-mediated reductions in body weight and in liver, eWAT, and iWAT weights (Fig 7D and E). Histologically, TIM-3 blockade restored the pathological features of adipocyte hypertrophy and hepatic steatosis (Fig 7F). Moreover, antibody-mediated TIM-3 blockade resulted in a pro-inflammatory shift, as evidenced by elevated expression of TNF-α, IL-1β, and IL-6, alongside a decline in IL-10 (Fig 7G and H). Simultaneously, the NF-κB pathway was reactivated upon TIM-3 blockade, as indicated by increased p-P65 levels and diminished IκB-α expression (Fig 7I). Notably, administration of the blocking antibody did not significantly affect food intake (Fig 7J). Collectively, these data indicate that the beneficial effects of rTs-SPI on obese mice were largely abrogated when TIM-3 was blocked, highlighting its crucial role in mediating the protective actions of rTs-SPI.

### rTs-SPI modulates macrophage polarization via TIM-3-dependent activation of the PI3K/AKT/mTOR pathway

**Fig 8:**
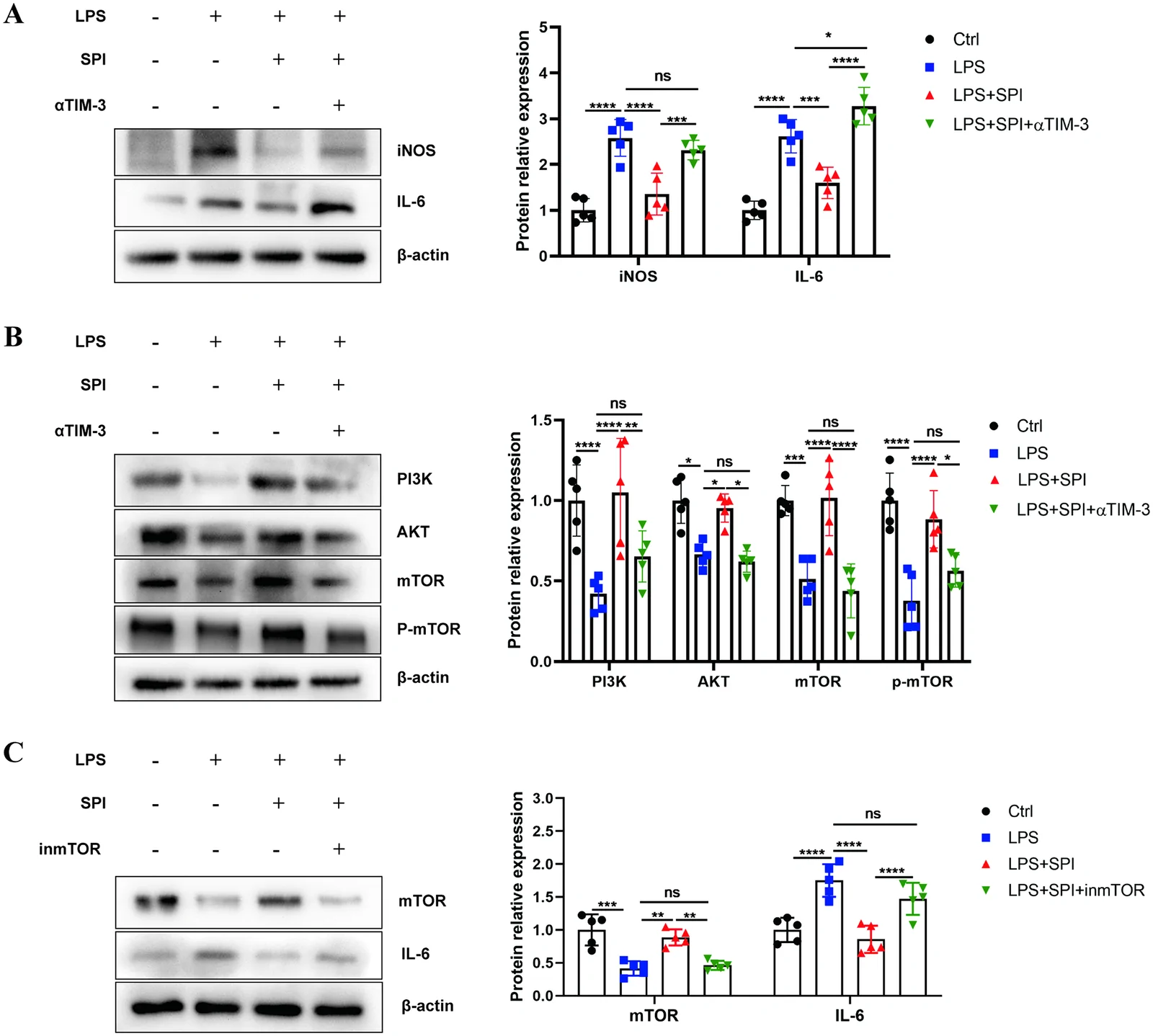
rTs-SPI modulates macrophage polarization via TIM-3-dependent activation of the PI3K/AKT/mTOR pathway. (A and B) BMDMs were pre-treated with rTs-SPI (10 µg/mL) in the presence of either an anti-TIM-3 monoclonal antibody (10 µg/mL) or an equal amount of rat IgG2a isotype control for 4 h. The anti-TIM-3 antibody was added 30 min prior to rTs-SPI. LPS (100 ng/mL) was then added for 24 h. Adherent cells were harvested for Western blot analysis of iNOS and IL-6 (A) and PI3K/AKT/mTOR pathway-related proteins (B). (C) BMDMs were incubated with the mTOR inhibitor rapamycin (50 nM) or vehicle control (0.1% DMSO) for 1 h, followed by rTs-SPI (10 µg/mL) for 4 h. Subsequently, LPS (100 ng/mL) was added, and the cells were cultured for an additional 24 h. Expressions of mTOR and IL-6 was determined by Western blot analysis. n = 5 biological replicates. ^*^p < 0.05, ^**^p < 0.01, ^***^p < 0.001, ^****^p < 0.0001, and ns means no statistical difference, two-way ANOVA with Sidak’s multiple comparison test. Data are expressed as mean ± SD.

Given that TIM-3 functions as an immune checkpoint and is highly expressed on macrophages, we reasonably speculated that rTs-SPI might exploit this molecule to direct macrophage polarization. To test this hypothesis, LPS-stimulated BMDMs were exposed to rTs-SPI in the presence or absence of anti-TIM-3 blocking mAb, and the expression of M1 markers (iNOS and IL-6) was quantified. PBS and LPS alone were used as negative and positive controls, respectively. As shown in Fig 8A, LPS stimulation resulted in significantly elevated levels of iNOS and IL-6 compared with the PBS control. Treatment with rTs-SPI alone markedly suppressed the LPS-induced upregulation of these markers; however, neutralization of TIM-3 completely reversed this suppression.

To further elucidate the intracellular signaling pathway downstream of TIM-3, the PI3K/AKT/mTOR axis was investigated. Compared with the negative control, LPS challenge reduced the levels of PI3K, AKT, total mTOR, and phosphorylated mTOR (p-mTOR) in BMDMs (Fig 8B). rTs-SPI treatment fully restored these proteins to approximately baseline levels, but this restorative effect was largely abrogated upon TIM-3 blockade (Fig 8B). Conversely, inhibition of mTOR not only prevented the rTs-SPI-mediated increase in mTOR but also restored IL-6 levels to those observed in the LPS-alone control group (Fig 8C). Collectively, these data reveal that rTs-SPI programs macrophages toward an anti-inflammatory phenotype via TIM-3-dependent activation of the PI3K/AKT/mTOR pathway.

## Discussion

The global obesity epidemic and its associated chronic low-grade inflammation present an urgent therapeutic need. A promising yet underexplored therapeutic strategy involves developing immunomodulatory molecules from helminths, which have co-evolved with mammalian hosts to finely regulate immune responses [37]. This study aimed to investigate whether a specific helminth-derived molecule, Ts-SPI, could directly ameliorate HFD-induced obesity and adipose tissue inflammation. We further sought to elucidate the underlying cellular and molecular mechanisms, with a focus on macrophage polarization.

Our principal findings demonstrate that recombinant Ts-SPI administration effectively attenuated HFD-induced weight gain, adiposity, hepatic steatosis, and systemic dyslipidemia in mice, and these effects were independent of changes in food intake. Crucially, rTs-SPI significantly suppressed adipose tissue inflammation, as evidenced by reduced expression of cytokines TNF-α, IL-1β, and IL-6, elevated IL-10 levels, inhibition of the TLR4/MyD88/NF-κB pathway, and a shift in CD4^+^ T cell subsets towards an anti-inflammatory profile, characterized by increased Th2 and Treg populations and a concomitant decrease in Th1 cells. We found these benefits to be closely associated with the ability of rTs-SPI to reprogram macrophage polarization within adipose tissue, favoring an anti-inflammatory M2 state over a pro-inflammatory M1 phenotype. This observation aligns with and extends earlier reports on the anti-inflammatory properties of helminth-derived products. For example, treatment with a small peptide from egg extracts of *Schistosoma japonicum* induces Tregs to prevent immune-related colitis and psoriasis [38]*. Schistosoma mansoni* SEA improves metabolic parameters by promoting adipose tissue M2 macrophages [17]. Similarly, our previous work indicated that crude extracts derived from *T. spiralis* conferred metabolic benefits [18], and Ts-SPI itself alleviates experimental colitis by inducing M2 polarization [24]. The novelty of the present study lies in identifying a single, defined parasite molecule, Ts-SPI, as a direct effector against diet-induced obesity and in clarifying a specific receptor-mediated signaling axis responsible for its action.

A pivotal and particularly intriguing finding of our work is the identification of the immune checkpoint molecule TIM-3 as a critical mediator of the effects of Ts-SPI. We provide multifaceted evidence supporting a functional Ts-SPI/TIM-3 axis. For instance, rTs-SPI upregulated TIM-3 expression in macrophages *in vitro*, *in vivo* blockade of TIM-3 using a neutralizing antibody largely prevented the anti-obesity and anti-inflammatory benefits conferred by rTs-SPI, and TIM-3 blockade abolished the capacity of rTs-SPI to suppress M1 marker expression in LPS-stimulated macrophages *in vitro*. While the role of TIM-3 in T cell exhaustion is well-established [39,40], its function on macrophages within metabolic contexts is nascent. Recent studies have connected TIM-3 to metabolic disorders. For example, increased TIM-3 expression was found to alleviate liver injury in a NASH model by regulating macrophage activation [29], and the miR-330-5p/TIM-3 axis has been implicated in macrophage polarization and insulin resistance in diabetes [35]. Our findings meaningfully advance this field by establishing TIM-3 not merely as a marker or a downstream component, but as a functional target for a parasite-derived immunomodulator in the context of obesity-associated inflammation. This establishes a novel conceptual link between helminth-mediated immunomodulation and host metabolic checkpoint pathways.

Mechanistically, we further discovered that the Ts-SPI/TIM-3 axis targets the PI3K/AKT/mTOR signaling pathway to drive macrophage polarization reprogramming. rTs-SPI restored the levels of PI3K, AKT, mTOR, and p-mTOR that were suppressed by LPS, while this effect was largely prevented by TIM-3 blockade. Furthermore, pharmacological inhibition of mTOR reversed the suppressive effect of rTs-SPI on M1 markers. The PI3K/AKT/mTOR pathway is a crucial regulator of cellular metabolism and is recognized as a key influencer of macrophage polarization, frequently promoting macrophage toward an M2 phenotype [41,42]. Our data imply that Ts-SPI activates this pathway to favor alternative activation by engaging TIM-3, providing a plausible intracellular signaling pathway connecting the initial cell surface event to the final phenotypic outcome. The adoptive transfer experiments and the conditioned medium studies on adipocytes establish causality that Ts-SPI acts via TIM-3 on macrophages to induce M2 polarization, which in turn alters the macrophage secretory profile, thereby reducing adipocyte inflammation and lipid accumulation, and ultimately improving systemic metabolism. This mechanism highlights the fundamental importance of macrophage-adipocyte crosstalk in mediating the effects of Ts-SPI. However, whether the immunomodulatory effects of rTs-SPI depend on its serine protease inhibitory activity or other molecular properties remains unclear. Given that many serpins function independently of their anti-protease activity [43,44], and that a *T. spiralis*-derived serpin promotes macrophage alternative activation independently of its enzyme inhibitory activity, potentially via receptor-dependent signaling [23], it is plausible that rTs-SPI may act through receptor-mediated signaling pathways beyond its enzymatic function. Future structure-function studies using enzymatic activity-deficient mutants will be essential to clarify this issue.

Despite these insights, several limitations of our study must be acknowledged. First, the *in vivo* experiments were performed once due to the large group sizes and extensive downstream analyses. Second, although our data demonstrate that rTs-SPI modulates adipose tissue T cell populations, these findings are preliminary. The mechanistic relationship between TIM-3⁺ macrophages and the observed T cell changes remains to be elucidated, and the functional contribution of T cells to the anti-obesity effects of rTs-SPI warrants further investigation. Third, the lack of a positive control in the TIM-3 blockade experiment precludes assessment of the antibody’s efficacy magnitude. Fourth, while our blocking antibody data indicate that TIM-3 is necessary for the effects of Ts-SPI, a direct physical interaction between Ts-SPI and TIM-3 protein remains to be formally proven. We cannot rule out the possibility that Ts-SPI acts upstream of TIM-3, modulating its expression through an intermediate signal. Finally, our *in vivo* TIM-3 blockade was systemic. Although macrophages are a likely primary site of action, TIM-3 is also expressed on T cells, NK cells, and dendritic cells. Thus, potential contributions of TIM-3 of these immune cell types to the observed metabolic phenotype cannot be discounted. To address these gaps, future studies should focus on: (i) validation in independent experimental cohorts with larger sample sizes; (ii) mechanistic dissection using conditional TIM-3 knockout, T cell–macrophage co-culture systems with TIM-3 blockade, or T cell-deficient mice; (iii) confirmatory studies using TIM-3 knockout mice or independent efficacy assays to definitively confirm the specificity of TIM-3 blockade-mediated effects in our system; (iv) defining the direct ligand-receptor relationship between Ts-SPI and TIM-3 using biochemical and structural approaches; and (v) employing cell-specific TIM-3 knockout mice to clearly establish the intrinsic role of macrophage TIM-3 in mediating the effects of rTs-SPI.

## Conclusion

**Fig 9:**
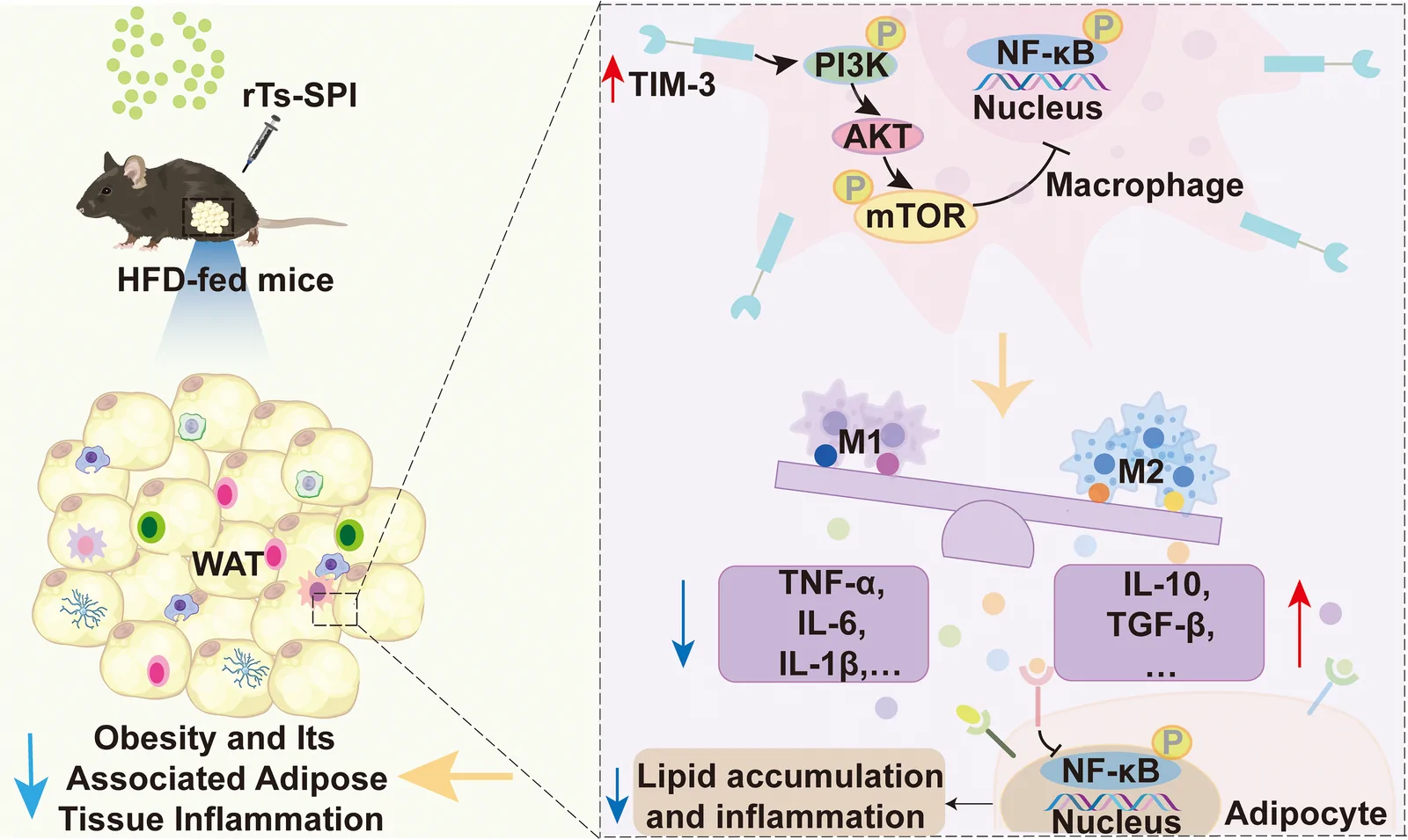
Schematic diagram of the proposed mechanisms underlying the protective effects of rTs-SPI against HFD-induced obesity and adipose tissue inflammation. rTs-SPI upregulates TIM-3 on macrophages, thereby activating the PI3K/AKT/mTOR pathway, driving protective M2 polarization, and ultimately alleviating HFD-induced obesity and the associated adipose tissue inflammation.

This study identifies the helminth-derived molecule Ts-SPI as a potent agent capable of ameliorating HFD-induced obesity and metabolic inflammation. We outline a novel mechanism whereby Ts-SPI, potentially by engaging TIM-3 on macrophages, activates the PI3K/AKT/mTOR pathway to drive protective M2 polarization, thereby resolving adipose tissue inflammation and improving systemic metabolism (Fig 9). These findings not only advance our understanding of parasite-host metabolic communication but also highlight the TIM-3 checkpoint as a promising target for immunometabolism intervention. We introduce the concept of “helminth-inspired checkpoint modulation” as a new strategic framework for treating metabolic diseases and provide new insight into parasite-host immune crosstalk. Ts-SPI itself represents a promising biologic template for developing novel, pathogenesis-targeted therapeutics against obesity and its inflammatory complications. Future work aimed at advancing these findings into more complex preclinical models and, ultimately, toward clinical application holds considerable promise.

## Materials and methods

### Ethics statement

Animal experiments were approved and performed in accordance with the guidelines of the Ethics Committee of Shanxi Medical University (Project ID: SYDL2023010).

### Animals, diets, and murine obesity model

Five-week-old male C57BL/6J mice were purchased from Beijing SPF Biotechnology Co., Ltd. (Beijing, China). All mice were maintained under pathogen-free conditions with suitable humidity and temperature. The obesity model was established as previously described [45]. In brief, C57BL/6J mice were fed a high-fat diet (HFD; 60 kcal% fat; TROPHIC Animal Feed High-Tech Co., China) for 10 weeks. As a negative control, mice received a normal diet (ND; 10 kcal% fat; TROPHIC Animal Feed High-Tech Co., China).

### Preparation of recombinant Ts-SPI and treatment

Recombinant Ts-SPI (rTs-SPI) was prepared according to our previous report [46]. Briefly, cDNA from the muscle larvae *T. spiralis* was used as the amplification template, and the amplified DNA sequence was subcloned into the pCold I vector. Then, the recombinant proteins were expressed in *Escherichia coli Rosetta-gami* (DE3), purified, removed of endotoxin using an endotoxin removal kit (Abbkine, China), and stored at -80°C.

To evaluate the role of rTs-SPI in obesity, male C57BL/6J mice were randomly assigned to three groups (n = 9) and maintained on either ND or HFD for 10 weeks. Beginning at week 0, HFD-fed mice were administered 200 µL of rTs-SPI (80 µg per mouse) in ISA206 adjuvant via intraperitoneal injection. Three more booster immunizations (50 µg per mouse each) were subsequently administered at 10-day intervals. An equal volume of PBS was used as a control. The dosing regimen (dose, route, frequency, and timing) for rTs-SPI administration was optimized from established protocols, with modifications based on our previous study [25] and published literature [23]. All mice had ad libitum access to food and water. The body weight was recorded weekly, and the food intake was recorded every other day. At the end of the experimental period (week 10), mice were fasted for 12 h, and blood samples were collected for plasma analysis. Intact liver, eWAT, and iWAT were carefully and quickly removed for weight measurement. In addition, liver, eWAT, iWAT, and brown adipose tissue tissue (BAT) were collected for further analysis.

### Isolation of the adipose tissue stromal vascular fraction

Isolation of the stromal vascular fraction (SVF) from adipose tissue was performed as previously described [47]. In brief, epididymal adipose tissues from rTs-SPI-treated and untreated mice were collected, finely minced, and digested for 1 h at 37 °C in HEPES buffer (pH 7.4) containing 0.5 g/L of type II collagenase from *Clostridium histolyticum* (Solarbio, China) and 2% (w/v) dialyzed BSA (Sigma-Aldrich, USA). The disaggregated adipose tissue was filtered through 100-μm nylon mesh. The filtrate was then centrifuged (350g, 10 min, room temperature), and the supernatant was discarded. The obtained SVF pellet was resuspended in erythrocyte lysis buffer (eBioscience, USA) for flow cytometric analysis.

### RAW264.7 cell lines, BMDMs preparation, and *in vitro* stimulation

RAW264.7 cells were cultured in Dulbecco’s Modified Eagle’s Medium (DMEM) supplemented with 10% fetal bovine serum (FBS), 100 U/mL penicillin, and 100 μg/mL streptomycin at 37 °C in a humidified atmosphere with 5% CO_2_. DMEM, FBS, and penicillin/streptomycin antibiotic solutions were purchased from Thermo Fisher Scientific (Waltham, MA). BMDMs were prepared and cultured as previously described [23]. In brief, bone marrow cells were flushed from the long bones of C57BL/6J mice, seeded, and cultured in DMEM with 10% FBS and 40 ng/mL recombinant mouse (rm) M-CSF (Sino Biological, China) at 37 °C with 5% CO_2_ for 48 h. Subsequently, all adherent cells were cultured in DMEM with 10% FBS and 40 ng/mL rm M-CSF for 72 h.

To investigate the immunoregulatory effect of rTs-SPI on macrophages, RAW264.7 cells and BMDMs were incubated with rTs-SPI (10 µg/mL) alone or co-incubated with LPS (100 ng/mL, Sigma-Aldrich, USA) for 24 h. The dosing regimen was determined based on previous report with slight modifications [23]. Cells challenged with LPS (100 ng/mL) or IL-4 (20 ng/mL, Novoprotein, China) were used as controls. Thereafter, the cells were harvested for immediate flow cytometric analysis or processed for western blot and quantitative real-time PCR to assess polarization status.

### Differentiation of 3T3-L1 preadipocytes and treatment with rTs-SPI-conditioned medium from BMDMs

3T3-L1 preadipocytes were differentiated to 3T3-L1 mature adipocytes as previously reported [48]. In brief, 3T3-L1 preadipocytes were seeded in growth medium and cultured at 37 °C in a humidified incubator with 5% CO_2_ until reaching 90–100% confluence. Forty-eight hours after the cells reached confluence, adipocyte differentiation was initiated by replacing the growth medium with differentiation medium. The total differentiation time was 14 days. To mimic the obese model *in vivo*, 3T3-L1 adipocytes were treated with cell culture medium containing the indicated concentrations of FFA (palmitic acid (0.25 mM) and oleic acid (0.5 mM); Kunchuang Biotechnology, China).

To determine whether rTs-SPI-modulated macrophages affect adipocyte inflammation in obesity-related microenvironment, BMDMs were incubated with or without rTs-SPI (10 µg/mL) for 24 h. Then the medium was collected and centrifuged at 2,000 × *g* for 5 min. The supernatant was concentrated using a 3 KDa ultrafiltration tube and sterilized by filtration through a 0.22 µm sterile filter. The culture medium collected from BMDMs with or without rTs-SPI pretreatment was designated as rTs-SPI-conditioned medium (SM) and control medium (CM), respectively. Thereafter, the FFA-stimulated mature adipocytes were co-incubated with multiple dilutions of SM or CM for 24 or 48 h. At the end, lipid droplets, the inflammatory cytokines TNF-α, IL-1β, and IL-6, the chemokine MCP-1, and triacylglycerol (TG) levels were measured.

### Adoptive transfer experiment

To further confirm whether rTs-SPI-conditioned macrophages mediate the anti-obesity and anti-inflammatory effects, an adoptive transfer experiment was performed. Five-week-old male C57BL/6J mice were randomly assigned to two groups (n = 5) and maintained on HFD for 10 weeks. BMDMs were pre-incubated with rTs-SPI (10 µg/mL) or PBS for 24 h. Thereafter, PBS- or rTs-SPI-treated BMDMs (2x10^6^ cells per mouse) were adoptively transferred by intravenous injection into HFD-fed mice once weekly from week 7 to week 10, for a total of four administrations. This regimen was selected based on established protocols [49–51]. The experimental design is graphically depicted in Fig 6A. Body weight and daily food intake were monitored. Liver, eWAT, and iWAT were harvested for weight determination and subsequent analysis.

### *In Vivo* Blockade of TIM-3

To determine the contribution of TIM-3 to the anti-obesity effects of rTs-SPI, *in vivo* TIM-3 blockade was performed. Five-week-old male C57BL/6J mice were randomly assigned to two groups (n = 5) and maintained on HFD for 10 weeks. Beginning at week 0, each mouse received an intraperitoneal injection of either an anti-TIM-3 monoclonal antibody (clone RMT3–23; 200 µg per mouse, Bio X Cell, USA) or an equivalent dose of isotype control mAb (rat IgG2a). The injections were administered twice weekly throughout the experimental period, and the blocking regimen was selected based on a GEO-deposited study (GSM8582691) and established protocol in the literature [52]. One day after the initial antibody treatment, all mice were treated intraperitoneally with rTs-SPI (80 µg per mouse). Three booster immunizations with rTs-SPI (50 µg per mouse) were subsequently administered at 10-day intervals. The experimental schedule is illustrated in Fig 7C. Body weight and daily food intake were recorded. Liver, eWAT, and iWAT were excised, weighed, and subjected to further analysis.

### TIM-3 blocking and mTOR inhibition in BMDMs

To elucidate the regulatory mechanism by which rTs-SPI controls macrophage polarization, BMDMs were pre-treated with rTs-SPI (10 µg/mL) in the presence of either an anti-TIM-3 monoclonal antibody (10 µg/mL, Bio X Cell, USA) or an equal amount of rat IgG2a isotype control for 4 h at 37 °C in a humidified incubator with 5% CO_2_. The anti-TIM-3 antibody was added 30 min prior to rTs-SPI to ensure adequate receptor blockade. Subsequently, LPS (100 ng/mL, Sigma-Aldrich, USA) was added to induce M1 polarization, and the cultures were continued for 24 h at 37 °C. After incubation, adherent cells were washed twice with ice-cold PBS, harvested by gentle scraping, and lysed in RIPA buffer supplemented with protease and phosphatase inhibitors for total protein extraction.

For the mTOR inhibition assay, BMDMs were first incubated with mTOR inhibitor rapamycin (50 nM, MCE, USA) or vehicle control (0.1% DMSO) for 1 h at 37 °C to achieve complete pathway blockade, as previously described [53,54]. The rapamycin stock solution (10 mM in DMSO) was freshly diluted in complete medium before each experiment. Following this pretreatment, the cells were incubated with rTs-SPI (10 µg/mL) for 4 h. Subsequently, LPS (100 ng/mL, Sigma-Aldrich, USA) was added, and the cells were cultured for an additional 24 h. After stimulation, cells were harvested as described above.

### Biochemical and ELISA analyses

Serum samples were obtained after centrifugation at 3,000 rpm for 10 min at 4 °C. Serum TC, TG, LDL, HDL, and FBG were determined using an automated biochemical analyzer (BioMajesty, Japan) at Taiyuan Hospital. Medium TG levels were quantified by a GPO-PAP enzyme-based colorimetric assay kit (Nanjing Jiancheng Bioengineering Institute, China). TNF-α and IL-6 concentrations in cell culture supernatants were measured using specific ELISA kits (TNF-α: CSB-E04741m-IS, sensitivity: 1.95 pg/mL; IL-6: CSB-E04639m-IS, sensitivity: 0.975 pg/mL; Cusabio, China). Plasma insulin was quantified with an ELISA kit (CEA448Mu, sensitivity: <2.65 pg/mL; Cloud-Clone, China). The insulin resistance index (HOMA-IR) was calculated using the formula: HOMA-IR = [fasting insulin level (mIU/L) × FBG level (mmol/L)]/ 22.5 [55].

### Histological analysis

For paraffin sectioning, iWAT, eWAT, BAT, and liver tissues were fixed in 10% neutral-buffered formalin for 24 h at room temperature, dehydrated through a graded ethanol series (70%, 80%, 95%, and 100%), cleared in xylene, embedded in paraffin wax, and sectioned at 4-μm thickness. H&E staining was performed according to standard protocols. Liver H&E staining was semiquantitatively scored using the NAFLD activity score (NAS) system, as previously described [56]. For ORO staining, frozen liver sections were immersed in optimal cutting temperature (OCT) compound (Solarbio, China), snap-frozen in liquid nitrogen-cooled 2-methylbutane, and stored at −80°C. Frozen sections (10-μm thick) were prepared using a cryostat (Leica CM1850, Germany), air-dried for 30 min, and fixed in 4% paraformaldehyde for 15 min. Sections were then stained with freshly prepared 0.5% ORO solution (Solarbio, China) and counterstained with hematoxylin. Lipid accumulation was quantified as the percentage of ORO-positive area per total field using ImageJ software (NIH, USA).

Immunohistochemical staining was performed on 4-μm paraffin-embedded sections. Briefly, sections were deparaffinized, rehydrated, and subjected to antigen retrieval by heating in sodium citrate buffer (10 mM, pH 6.0) at 95°C for 20 min. Nonspecific binding was blocked with 5% normal goat serum for 30 min at room temperature. Sections were then incubated overnight at 4 °C with the following primary antibodies: rabbit anti-mouse F4/80 (1:200; Thermo Fisher Scientific, USA), rabbit anti-mouse IFN-γ (1:200; UpingBio, China), rabbit anti-mouse IL-4 (1:200; Bioss, China), rabbit anti-mouse Foxp3 (1:200; UpingBio, China), rabbit anti-mouse iNOS (1:200; HUABIO, China), and rabbit anti-mouse CD206 (1:500; HUABIO, China). After three washes with PBS, sections were incubated with HRP-conjugated goat anti-rabbit IgG secondary antibody (1:500; Jackson ImmunoResearch, USA) for 1 h at room temperature. Images were captured using an AxioObserver Z1 microscope (Carl Zeiss, Germany).

### Flow cytometric analysis

SVF and BMDMs were washed and adjusted to 1 x 10^6^ cells/100 µL in FACS buffer. To minimize nonspecific binding, cells were incubated with anti-CD16/CD32 Fc block antibody (BioLegend, USA) for 10 min at 4 °C.

For cell surface marker staining, cells were pelleted by centrifugation at 300 × g for 5 min at 4 °C, resuspended in 100 µL FACS buffer, and then stained with the following fluorochrome-conjugated antibodies: CD4-FITC (BD, Cat# 561835, USA), CD25-APC (BD, Cat# 565135, USA), F4/80-APC (BioLegend, Cat# 157306, USA), CD16/32-FITC (BioLegend, Cat# 156614, USA), F4/80-PerCP-Cy5.5 (eBioscience, Cat# 45-4801-82, USA), and CD11c-FITC (eBioscience, Cat# 11-0114-81, USA). Cells were incubated for 30 min at 4 °C in the dark, then washed once with 2 mL FACS buffer (300 × g, 5 min, 4 °C).

For intracellular staining, cells were first stained for surface markers as described above, then fixed and permeabilized using the Foxp3/Transcription Factor Staining Buffer Set (eBioscience, USA) according to the manufacturer’s instructions. After centrifugation, the cells were stained with the following intracellular antibodies: IFN-γ-PE (BD, Cat# 562020, USA), IL-4-PE (BD, Cat# 562044, USA), Foxp3-PE (BD, Cat# 560414, USA), CD206-PE (BioLegend, Cat# 141706, USA), CD206-APC (eBioscience, Cat# 17-2061-82, USA), and TIM-3-PE (Elabscience, Cat# E-AB-F1192D, China). Cells were incubated for 45 min at 4 °C in the dark and subsequently washed once with FACS buffer (300 × g, 5 min, room temperature). After final resuspension in 500 µL FACS buffer, the samples were analyzed using a BD FACSCelesta flow cytometer (BD Biosciences, USA). Data analysis was performed using FlowJo software (version 10; Tree Star, USA).

### RNA isolation and quantitative real-time PCR

Total RNA was extracted from eWAT and cultured cells using TRIzol Reagent (Invitrogen, USA) following the manufacturer’s instructions. Genomic DNA contamination was eliminated using gDNA Removal Reagent (TransGen Biotech, China). Reverse transcription was performed using 4 µg of total RNA with the TransScript All-in-One First-Strand cDNA Synthesis SuperMix for quantitative real-time PCR (qRT-PCR) (TransGen Biotech, China) in a 20 µL reaction volume. qRT-PCR was performed on a 7500 Real-Time PCR machine (Applied Biosystems, USA) using the TransStart Top Green qPCR SuperMix kit (TransGen Biotech, China) according to the manufacturer’s protocol. The primer sequences are listed in S1 Table. β-actin was used as the reference housekeeping gene for normalization. Relative gene expression was calculated using the 2^-ΔΔCt^ model. Cycle threshold (Ct) values were determined by the instrument’s automatic baseline and threshold settings. Samples with Ct values >35 for any target gene were considered below the reliable detection limit and were excluded from statistical analysis for that specific gene. For statistical analysis, -ΔΔCt values (log2-transformed relative expression) were used to ensure normality.

### Western blot analysis

eWAT tissues and cells were lysed in ice-cold RIPA buffer (50 mM Tris-HCl, pH 7.4, 150 mM NaCl, 1% NP-40, 0.5% sodium deoxycholate, 0.1% SDS) supplemented with a protease inhibitor cocktail (Roche, Switzerland) and phosphatase inhibitors (10 mM sodium fluoride, 1 mM sodium orthovanadate, 10 mM β-glycerophosphate). Lysates were centrifuged at 12,000 × g for 15 min at 4 °C, and the supernatant was collected. Protein concentrations were determined using the BCA Protein Assay Kit (Pierce, USA).

Equivalent amounts of proteins (30–50 µg per lane) were separated by 10% SDS-PAGE and transferred onto a 0.45 µm PVDF membranes (Millipore, USA). Membranes were blocked with 5% non-fat milk in TBST (Tris-buffered saline with 0.1% Tween-20) for 1 h at room temperature with gentle agitation. For phosphorylated protein detection, 5% BSA in TBST was used as the blocking buffer to minimize nonspecific binding. Membranes were incubated overnight at 4 °C with the following primary antibodies: rabbit anti-mouse TLR4 (1:1000; CST, USA), MyD88 (1:1000; Wanleibio, China), p-IKKα/β (1:1000; CST, USA), IκB-α (1:1000; CST, USA), p-P65 (1:500; Bioss, China), iNOS (1:1000; HUABIO, China), IL-6 (1:1000; Cohesion, China), TNF-α (1:1000; Zenbio, China), Arg-1 (1:1000; Immunoway, China), IL-10 (1:2000; Abiowell, China), TGF-β (1:2000; Wanleibio, China), IL-1β (1:1000; Wanleibio, China), PI3K (1:1000; UpingBio, China), AKT (1:1000; Wanleibio, China), mTOR (1:1000; Wanleibio, China), p-mTOR (Ser2448; 1:1000; Abmart, China), and β-actin (1:1000; Wanleibio, China).

After three washes with TBST (10 min each), membranes were incubated with HRP-conjugated goat anti-rabbit IgG secondary antibodies (1: 10,000; CST, USA) for 1 h at room temperature. Following three additional washes with TBST, protein bands were visualized using the enhanced chemiluminescence (ECL) Detection Kit (Seven, China) and captured using a ChemiDoc XRS imaging system (Bio-Rad, USA). Band intensities were quantified using ImageJ gel analysis software (version 1.52; NIH, USA). β-actin was used as an internal control.

### Statistical analysis

Statistical analysis was performed using GraphPad Prism 8.0. Data are expressed as mean ± SD. Normality was assessed using the Shapiro–Wilk test for all datasets with n ≥ 5. For normally distributed data, comparisons between two groups were analyzed using unpaired two-tailed Student’s *t*-test, while comparisons among three or more groups were analyzed by one-way ANOVA with Tukey’s post-hoc test or two-way ANOVA with Sidak’s multiple comparison test. Statistical significance was defined as *p* < 0.05.

## Supporting information

S1 Fig Flow cytometric analysis of F4/80 ⁺ cells in BMDMs. (DOCX)

S1 Table Primer sequences used in qRT-RCR assays. (DOCX)

S1 Data Original images of western blots. (PDF)

S2 Data Excel spreadsheet containing the data points presented in Figs 1–8. (XLSX)
