# Supplementary material for: Serine protease inhibitor from Trichinella spiralis ameliorates diet-induced obesity and adipose tissue inflammation through TIM-3-dependent macrophage reprogramming
Source: PLoS Pathog. 2026 Sep 25;22(9):e1014606. doi: 10.1371/journal.ppat.1014606 (PMC13614578; doi:10.1371/journal.ppat.1014606)
Supplement: Supplementary file 1 [file ppat.1014606.s003.pdf]

Fig.2B

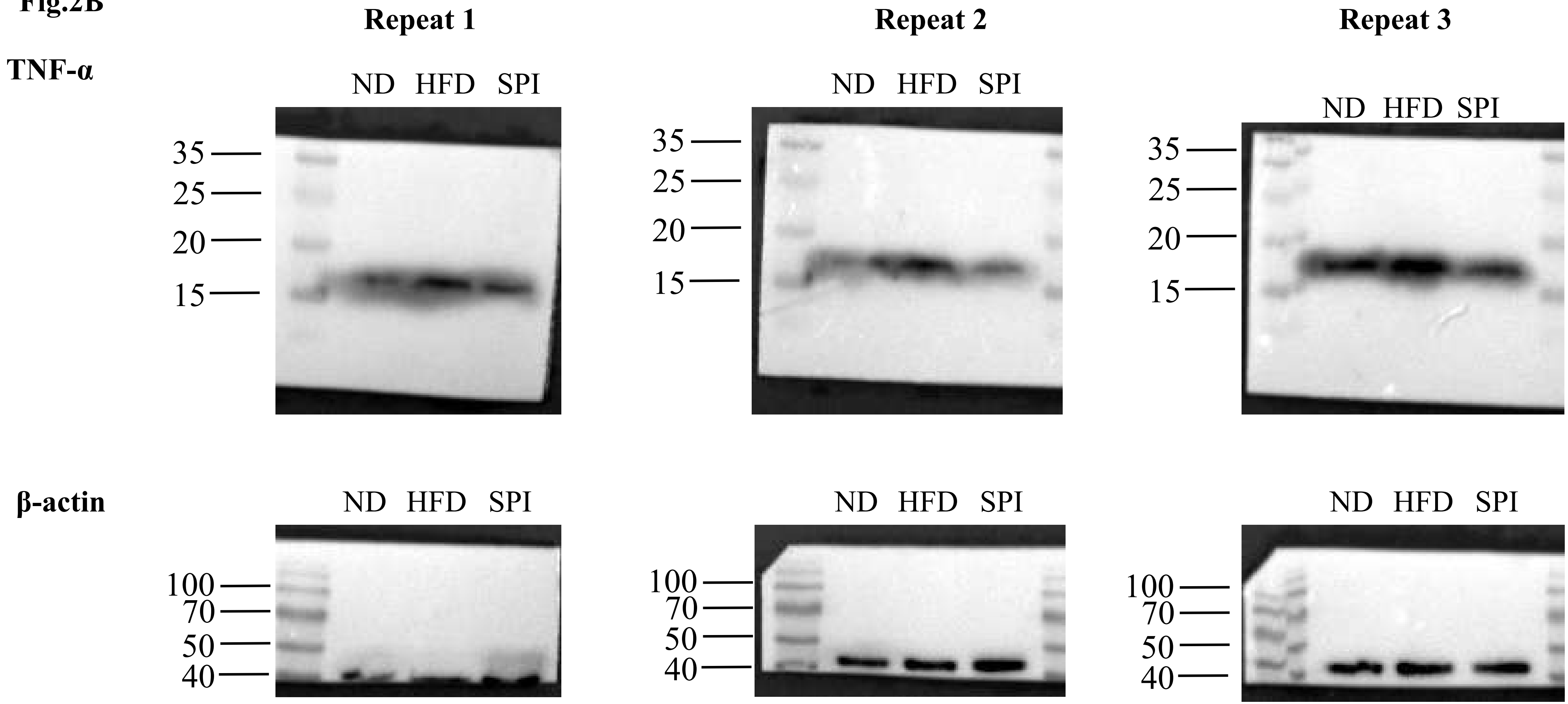
Repeat 1
Repeat 2
Repeat 3
TNF-α
ND HFD SPI
35
25
20
15
β-actin
100
70
50
40

Fig.2B

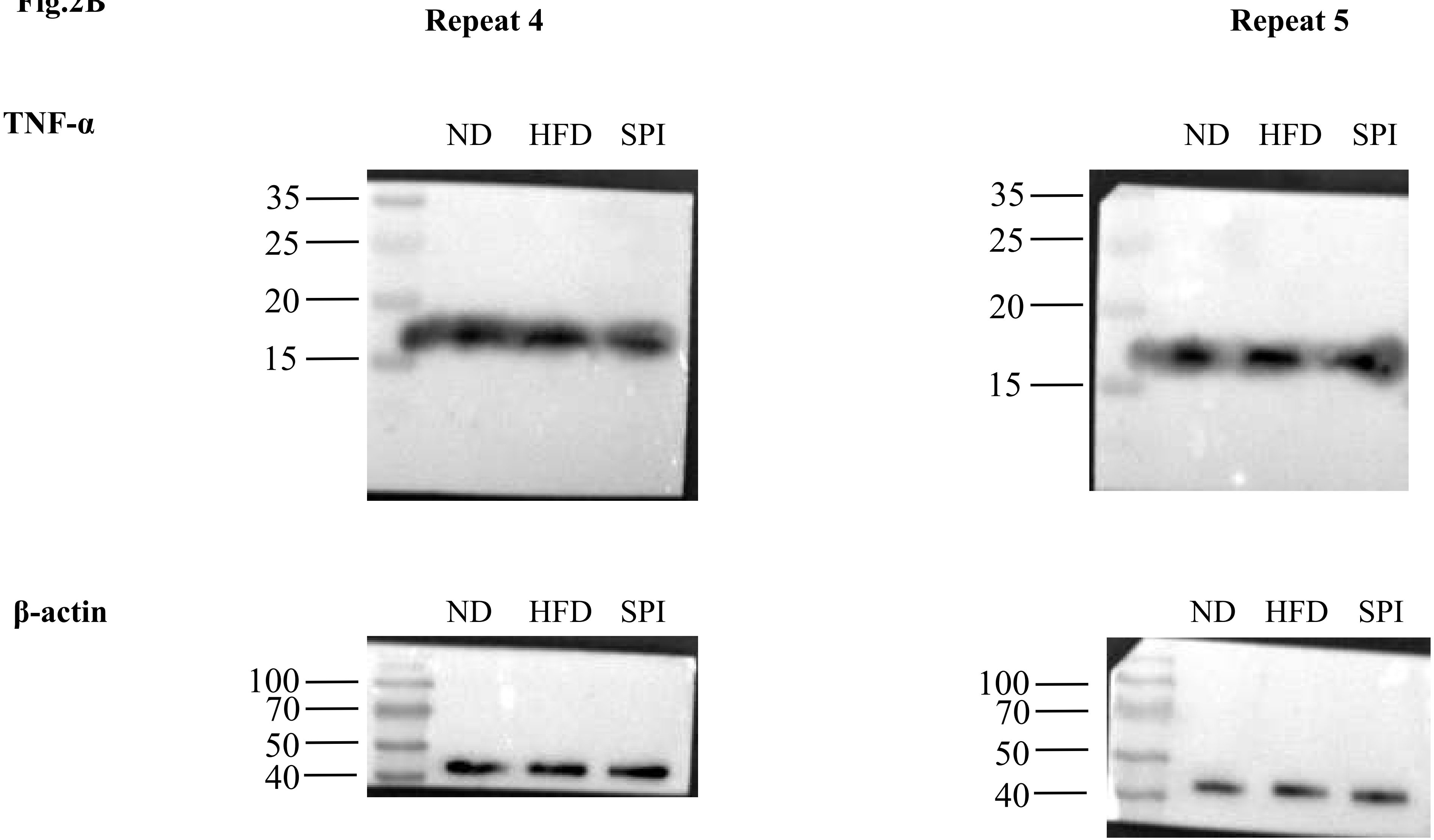
Repeat 4
Repeat 5
TNF-α
ND HFD SPI
35
25
20
15
ND HFD SPI
35
25
20
15
β-actin
ND HFD SPI
100
70
50
40
ND HFD SPI
100
70
50
40

Fig.2B

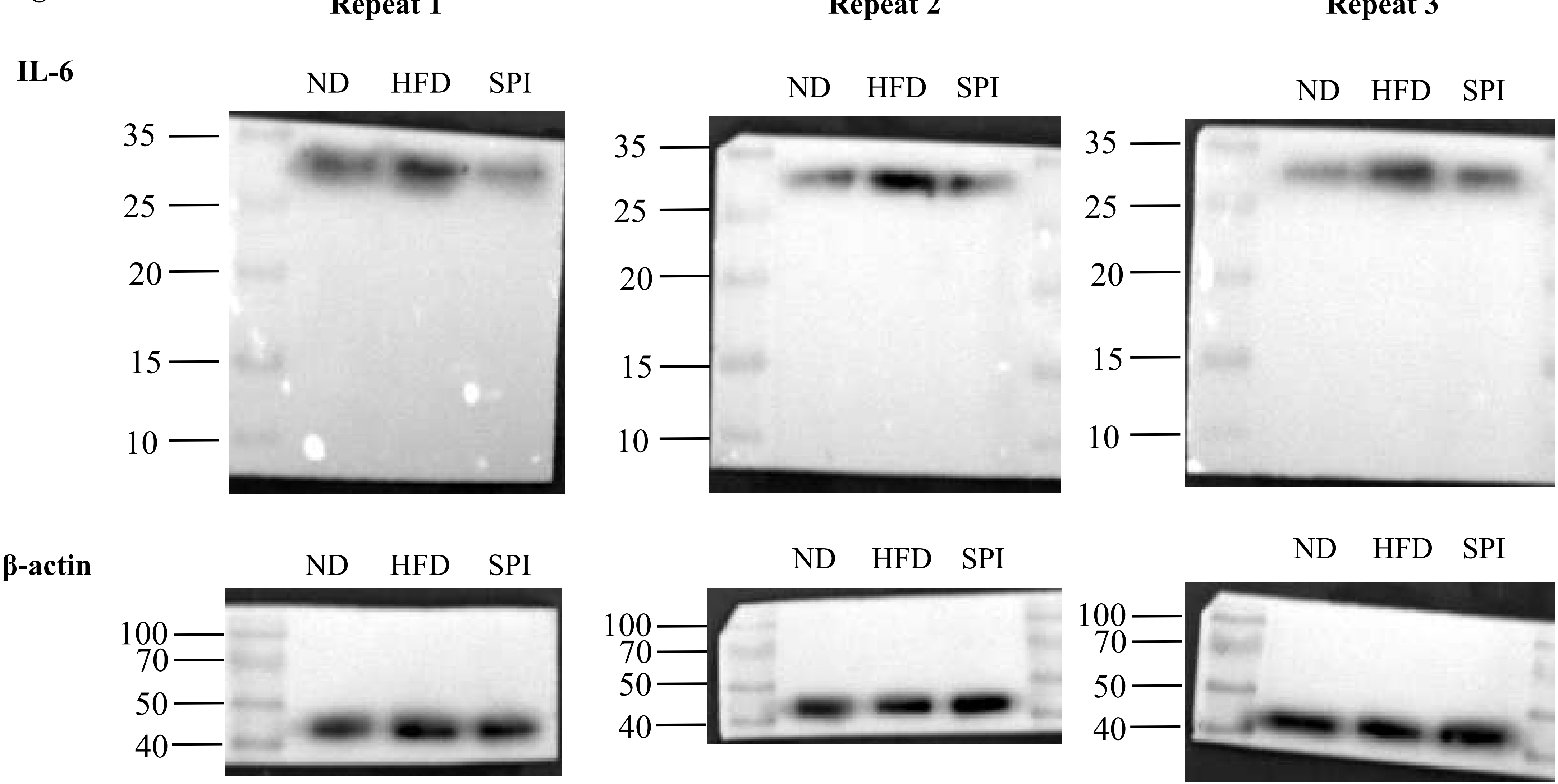
Repeat 1
Repeat 2
Repeat 3
IL-6
ND
HFD
SPI
35
25
20
15
10
β-actin
100
70
50
40

Fig.2B

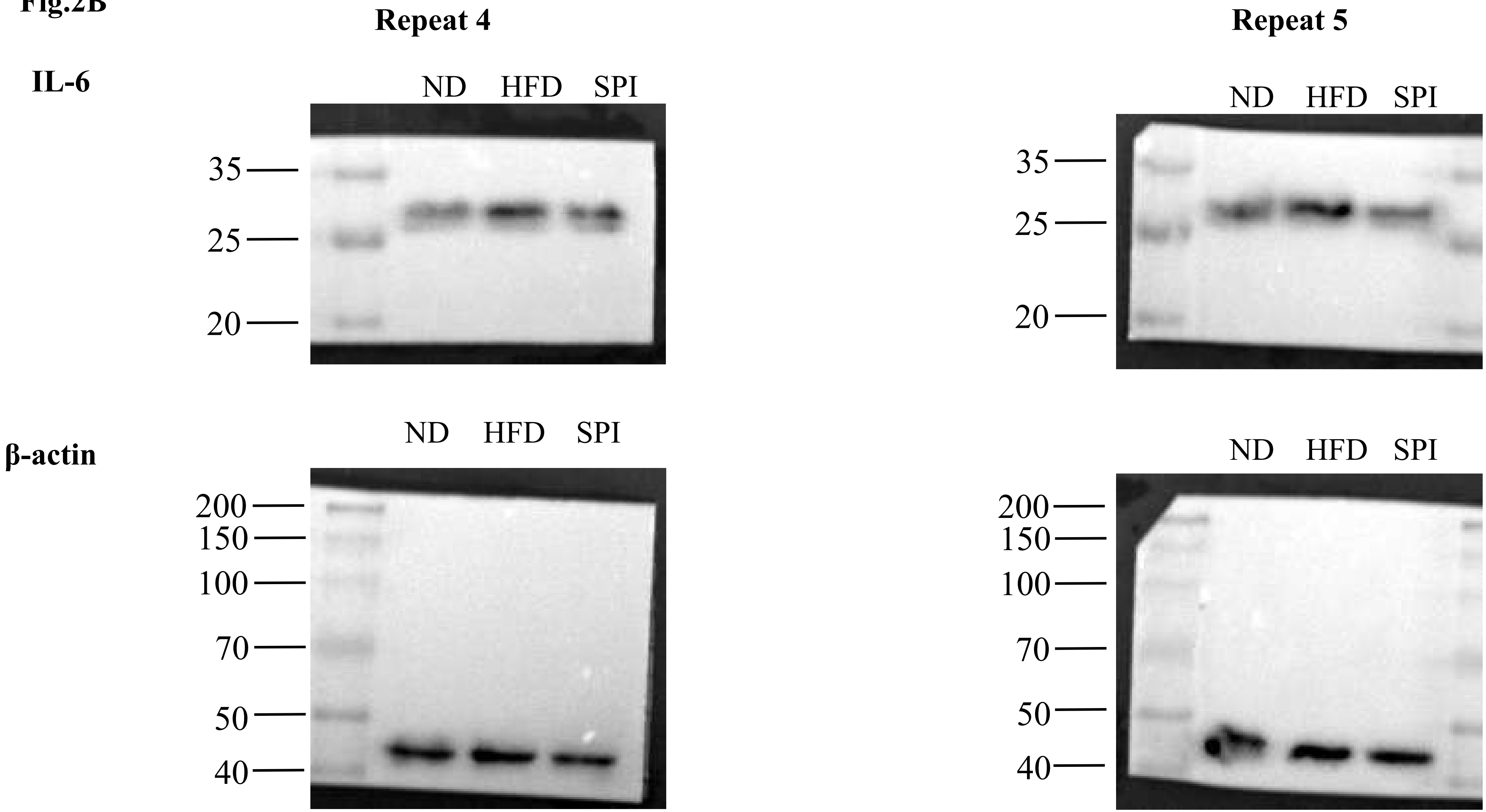
Repeat 4
Repeat 5
IL-6
ND HFD SPI
35
25
20
β-actin
ND HFD SPI
200
150
100
70
50
40

Fig.2B

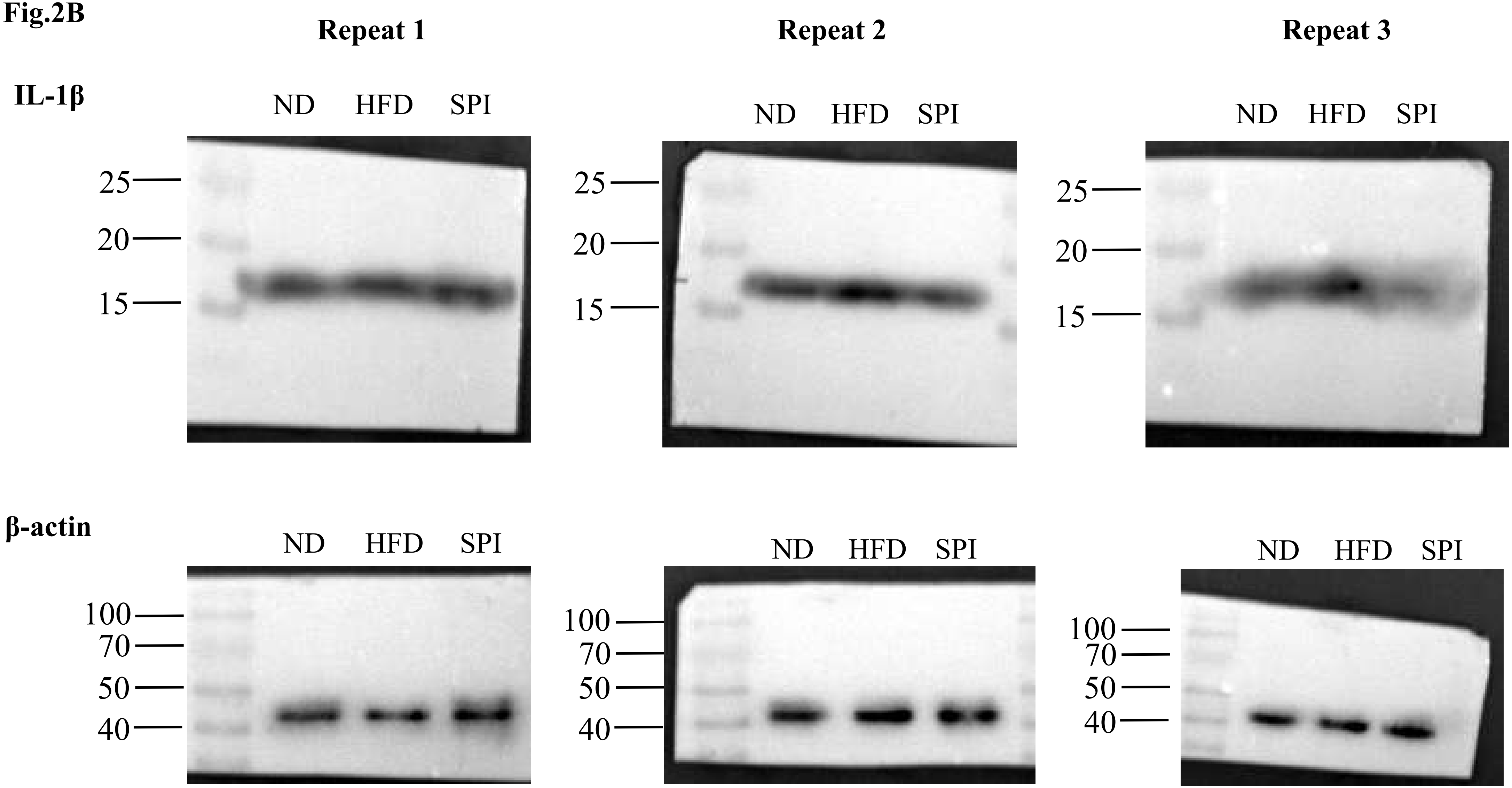
Repeat 1
Repeat 2
Repeat 3
IL-1β
ND HFD SPI
25
20
15
β-actin
100
70
50
40

Fig.2B

Repeat 4 **Repeat 5**

IL-1β

β-actin

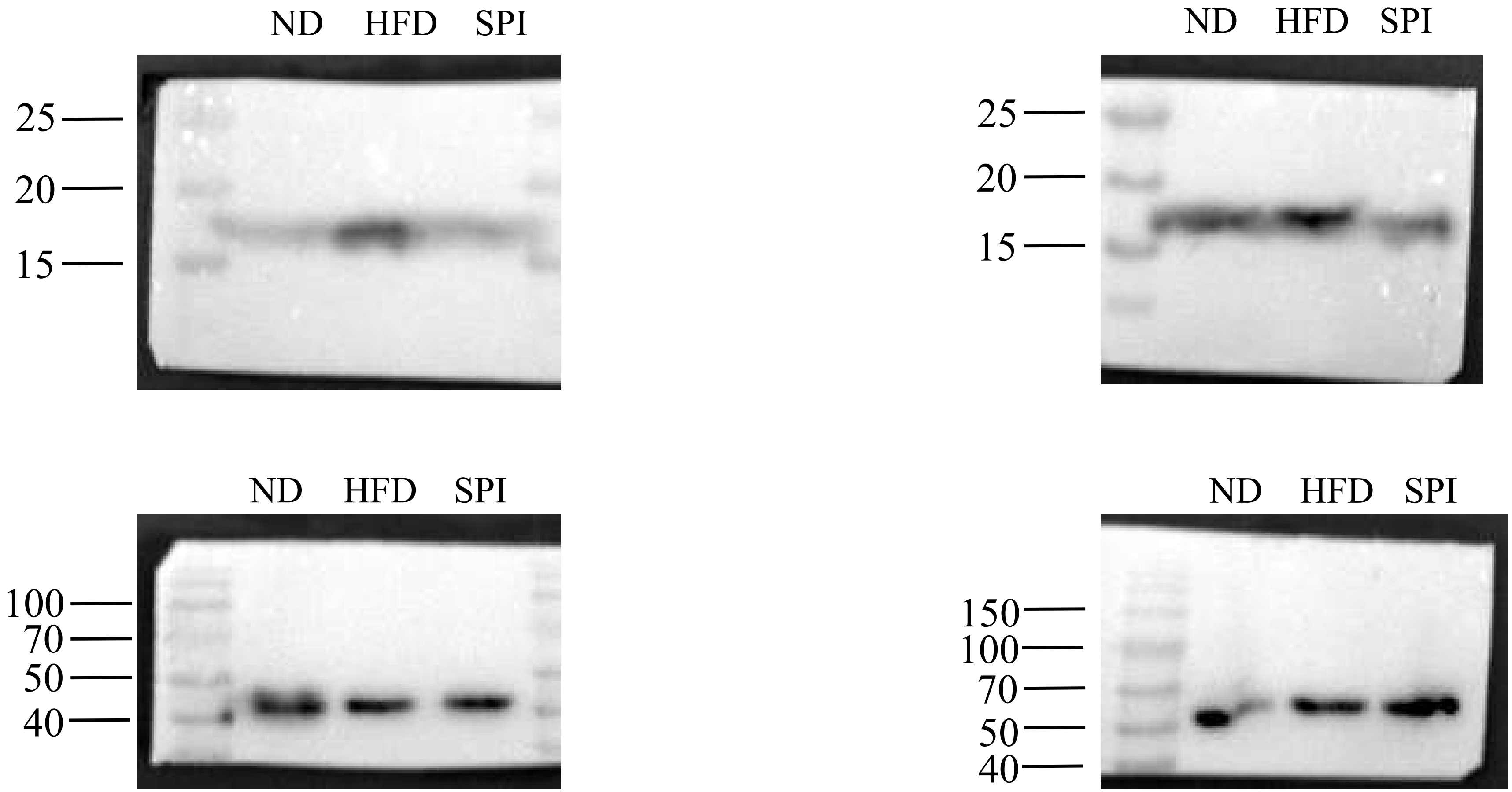

Fig.2B

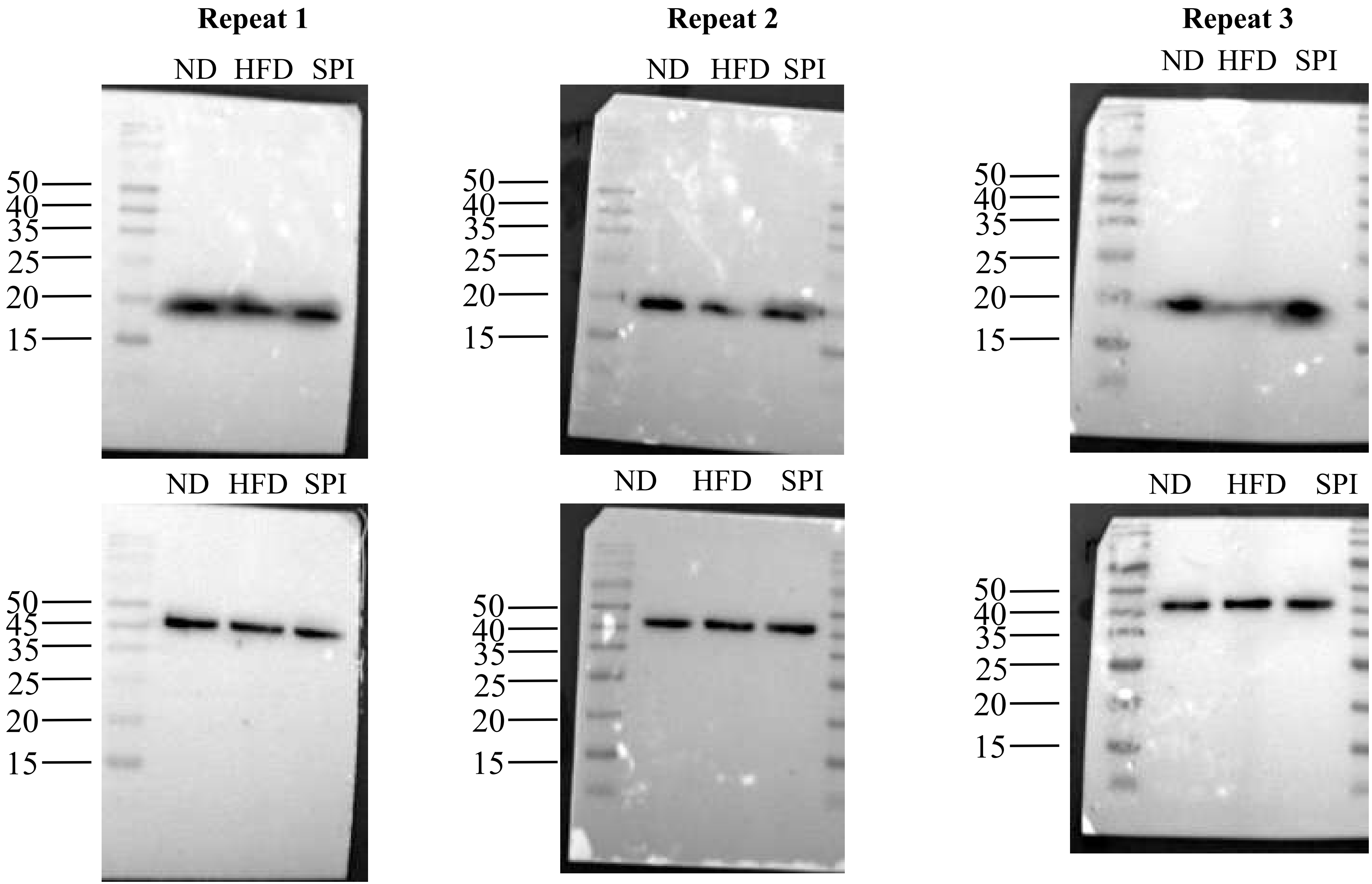
Repeat 1
Repeat 2
Repeat 3
IL-10
β-actin
ND HFD SPI
50
40
35
25
20
15
45

Fig.2B

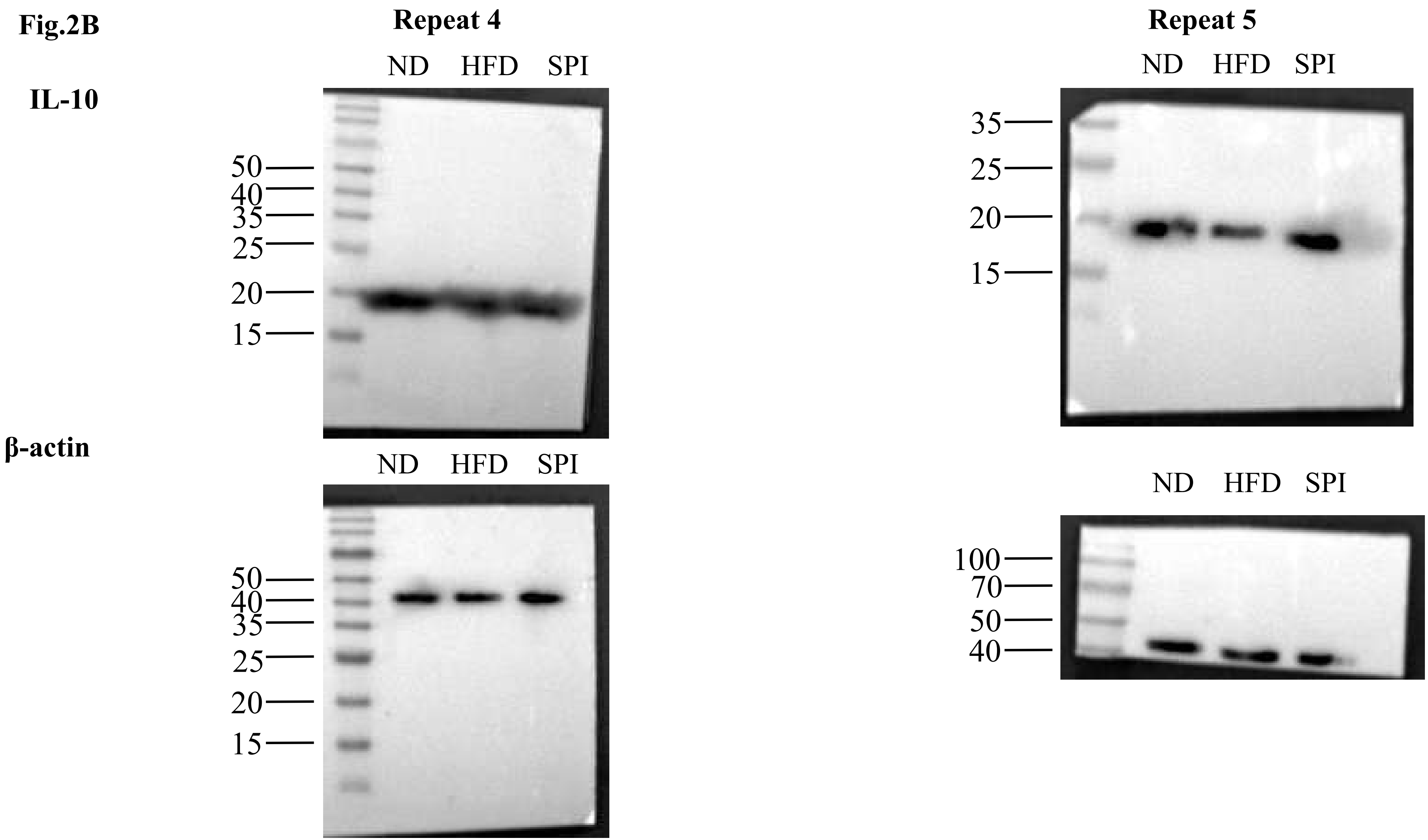

Fig.2C

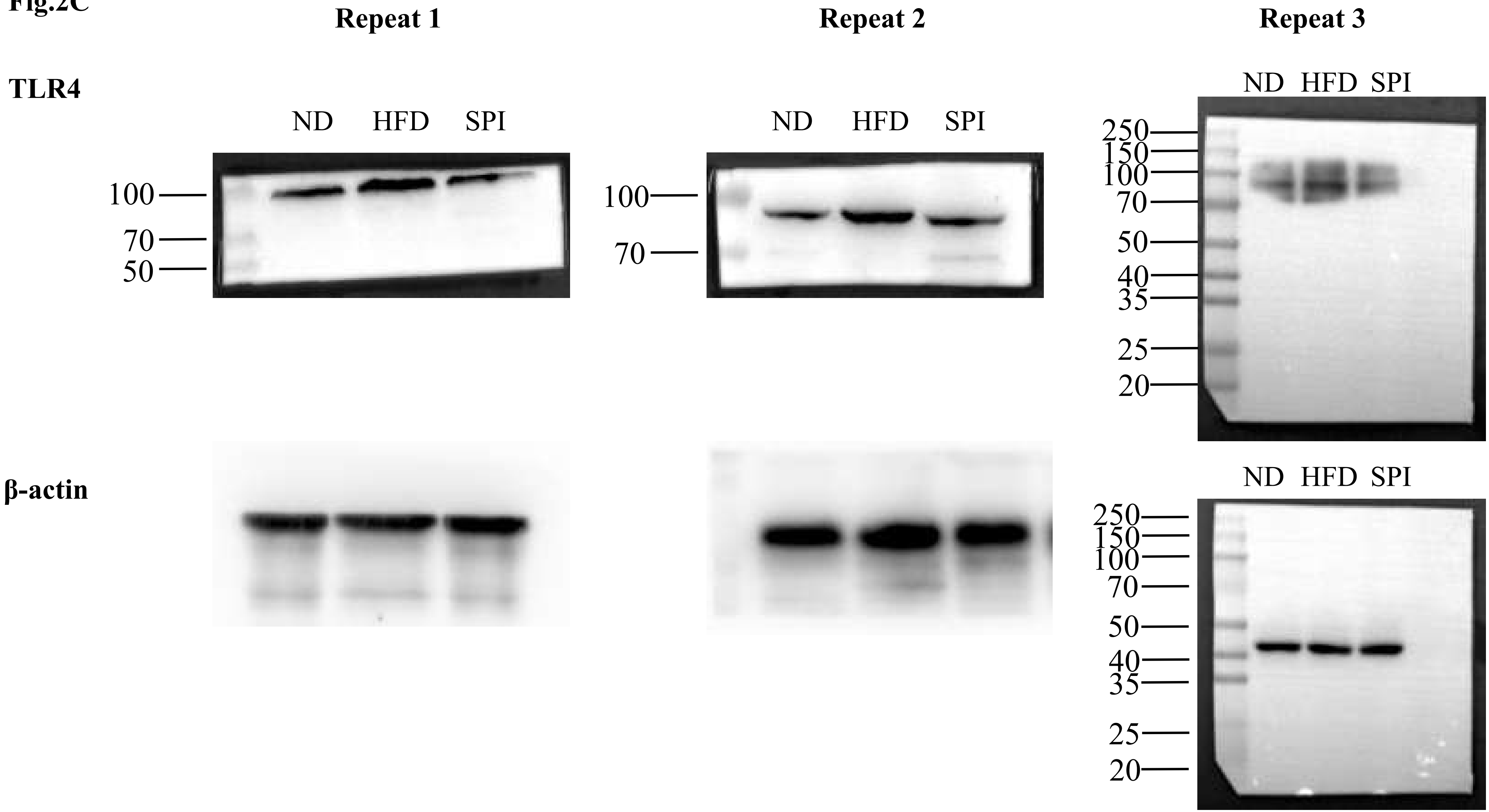
Repeat 1
Repeat 2
Repeat 3
TLR4
β-actin
ND
HFD
SPI
250
150
100
70
50
40
35
25
20

Fig.2C

Repeat 4 **Repeat 5**

TLR4

β-actin

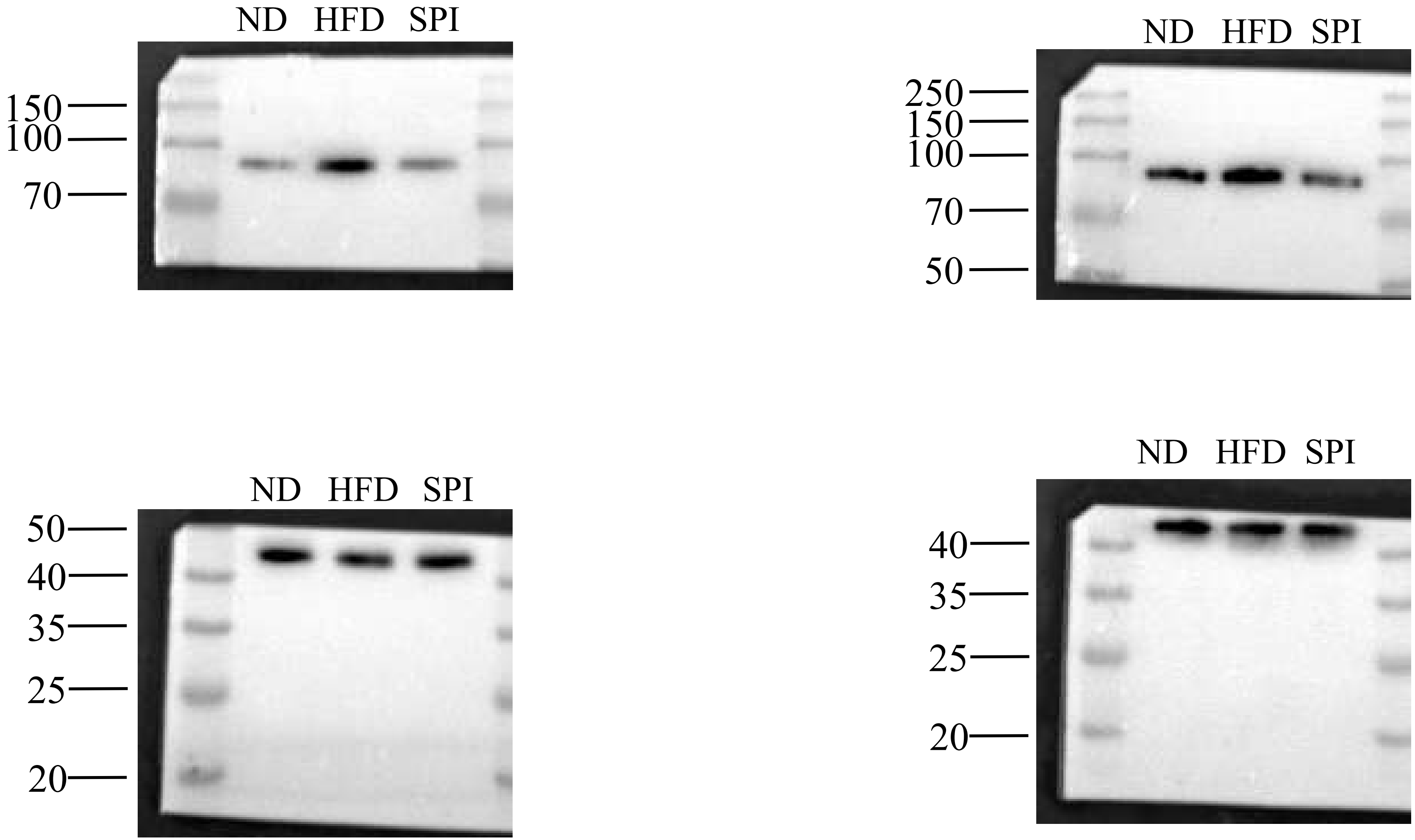

Fig.2C

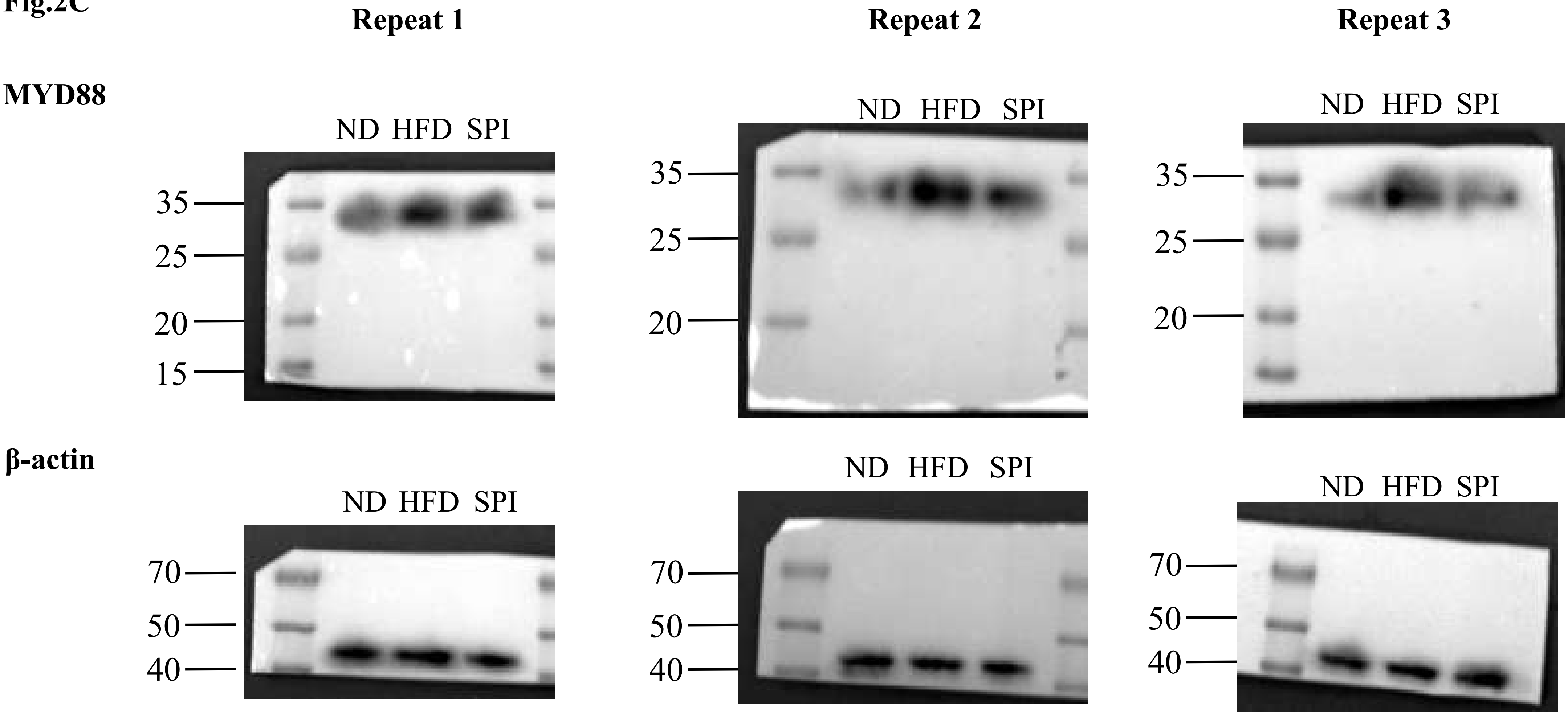

Repeat 1
Repeat 2
Repeat 3
MYD88
ND HFD SPI
35
25
20
15
β-actin
70
50
40

Fig.2C

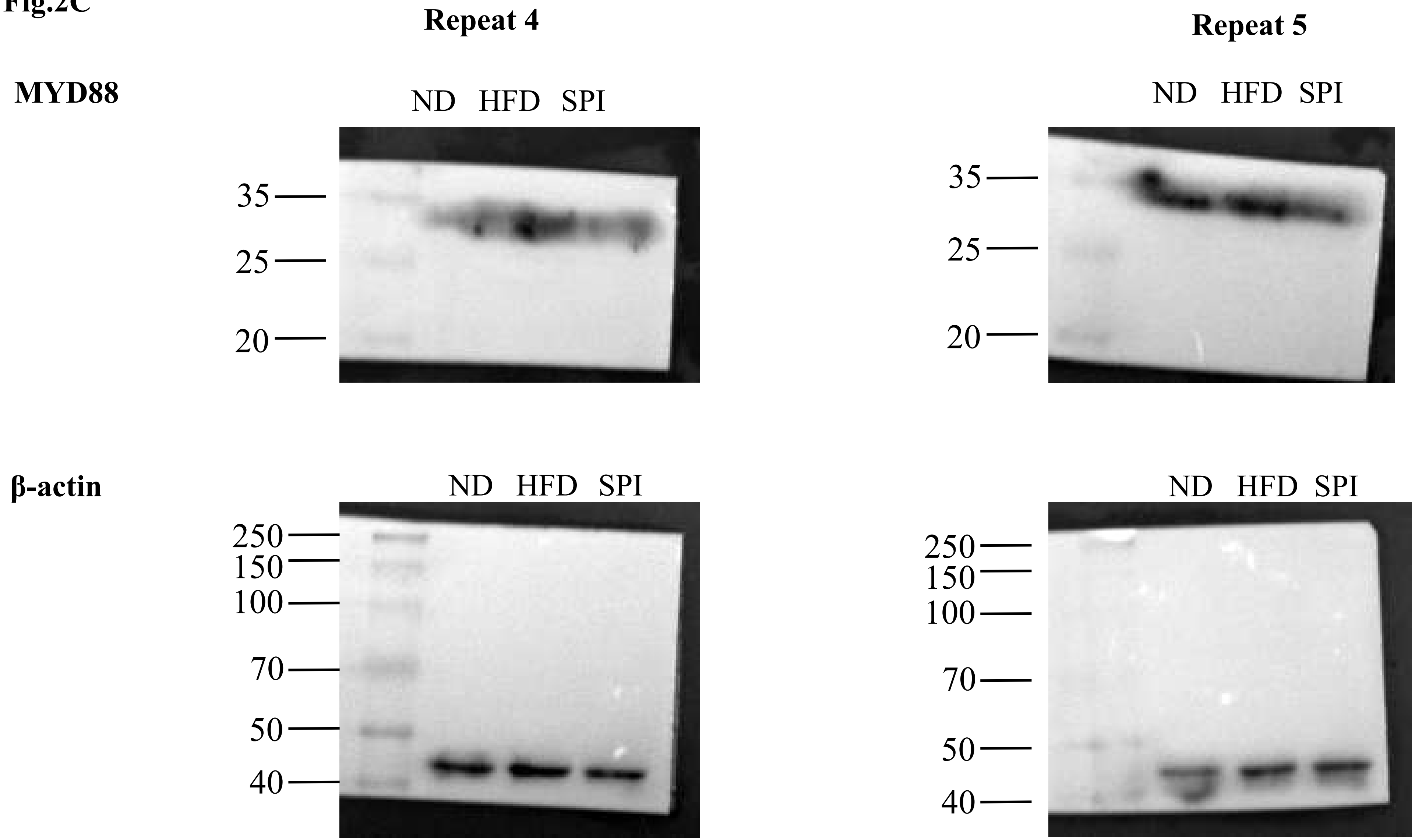
Repeat 4
Repeat 5
MYD88
ND HFD SPI
ND HFD SPI
35
25
20
35
25
20
β-actin
ND HFD SPI
ND HFD SPI
250
150
100
70
50
40
250
150
100
70
50
40

Fig.2C

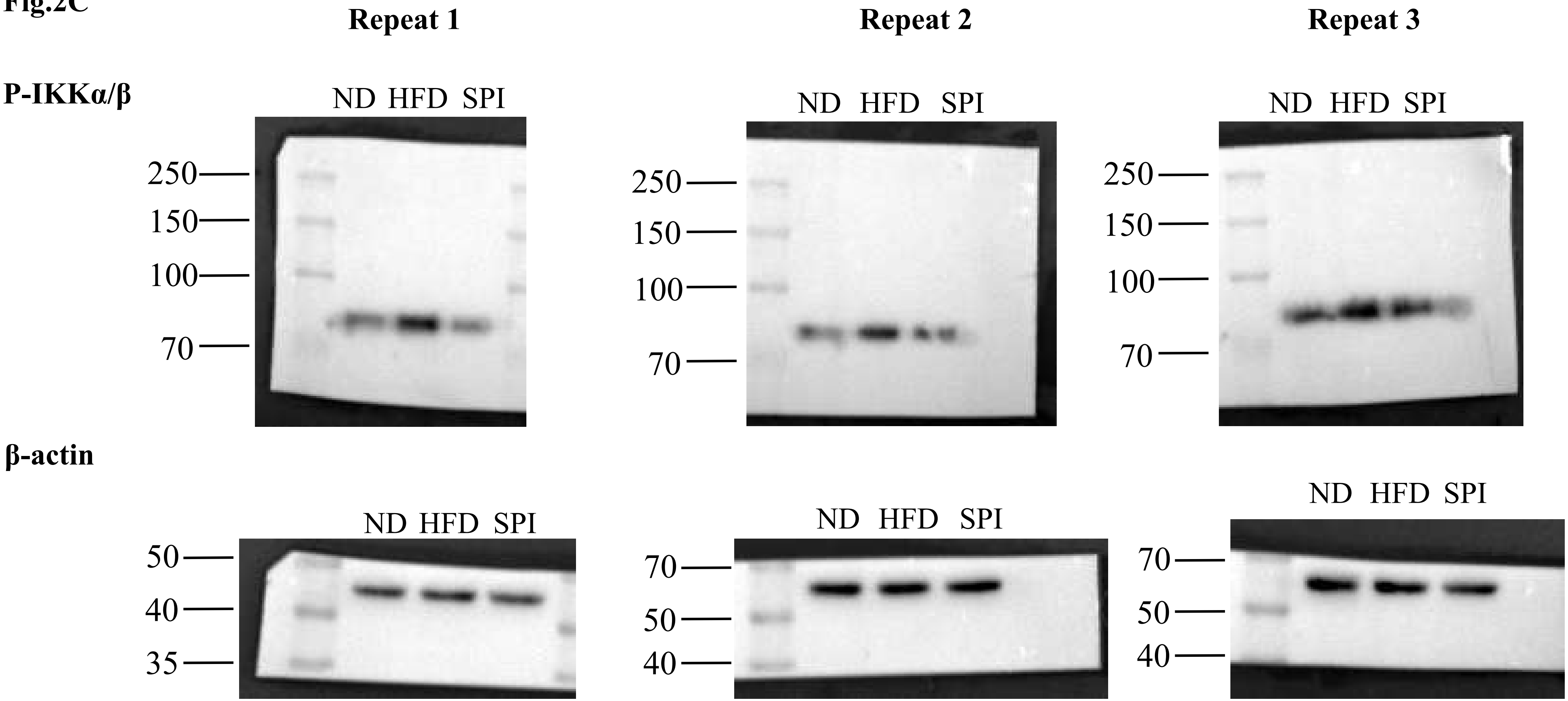
Repeat 1
Repeat 2
Repeat 3
P-IKKα/β
ND HFD SPI
250
150
100
70
β-actin
ND HFD SPI
50
40
35
70
50
40

Fig.2C

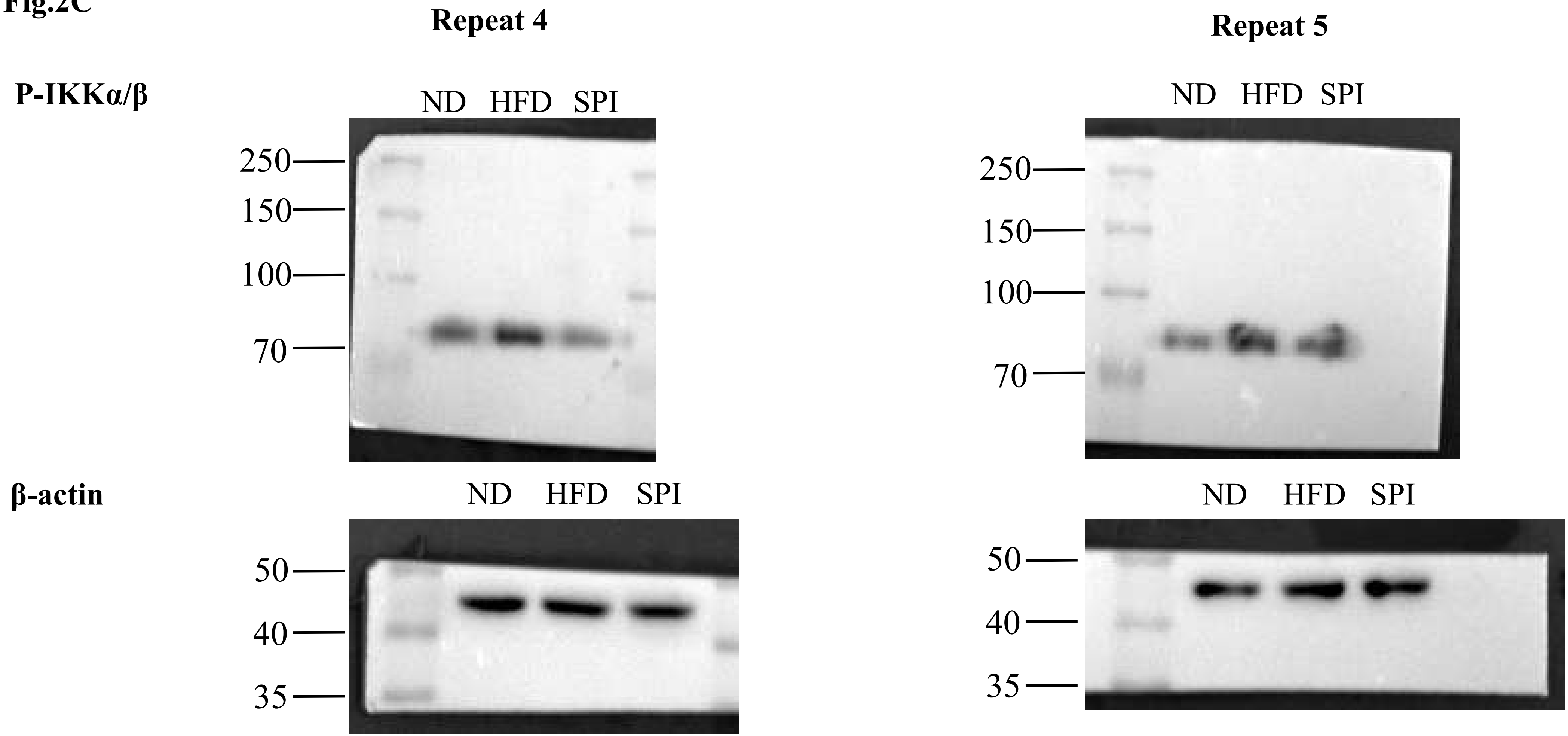
Repeat 4
Repeat 5
P-IKKα/β
ND HFD SPI
250
150
100
70
ND HFD SPI
250
150
100
70
β-actin
ND HFD SPI
50
40
35
ND HFD SPI
50
40
35

Fig.2C

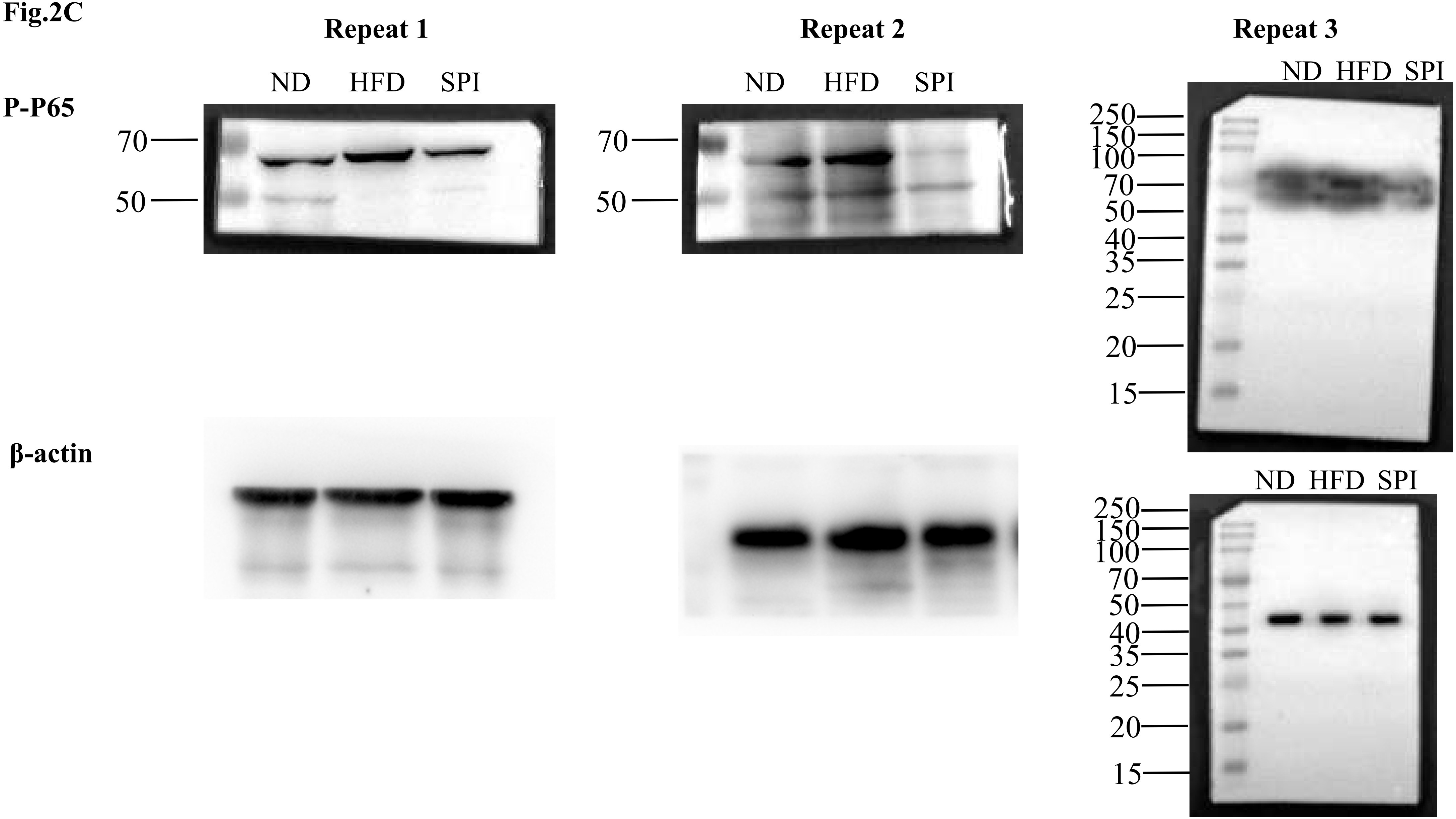
Repeat 1
Repeat 2
Repeat 3
P-P65
ND
HFD
SPI
70
50
250
150
100
40
35
25
20
15
β-actin

Fig.2C

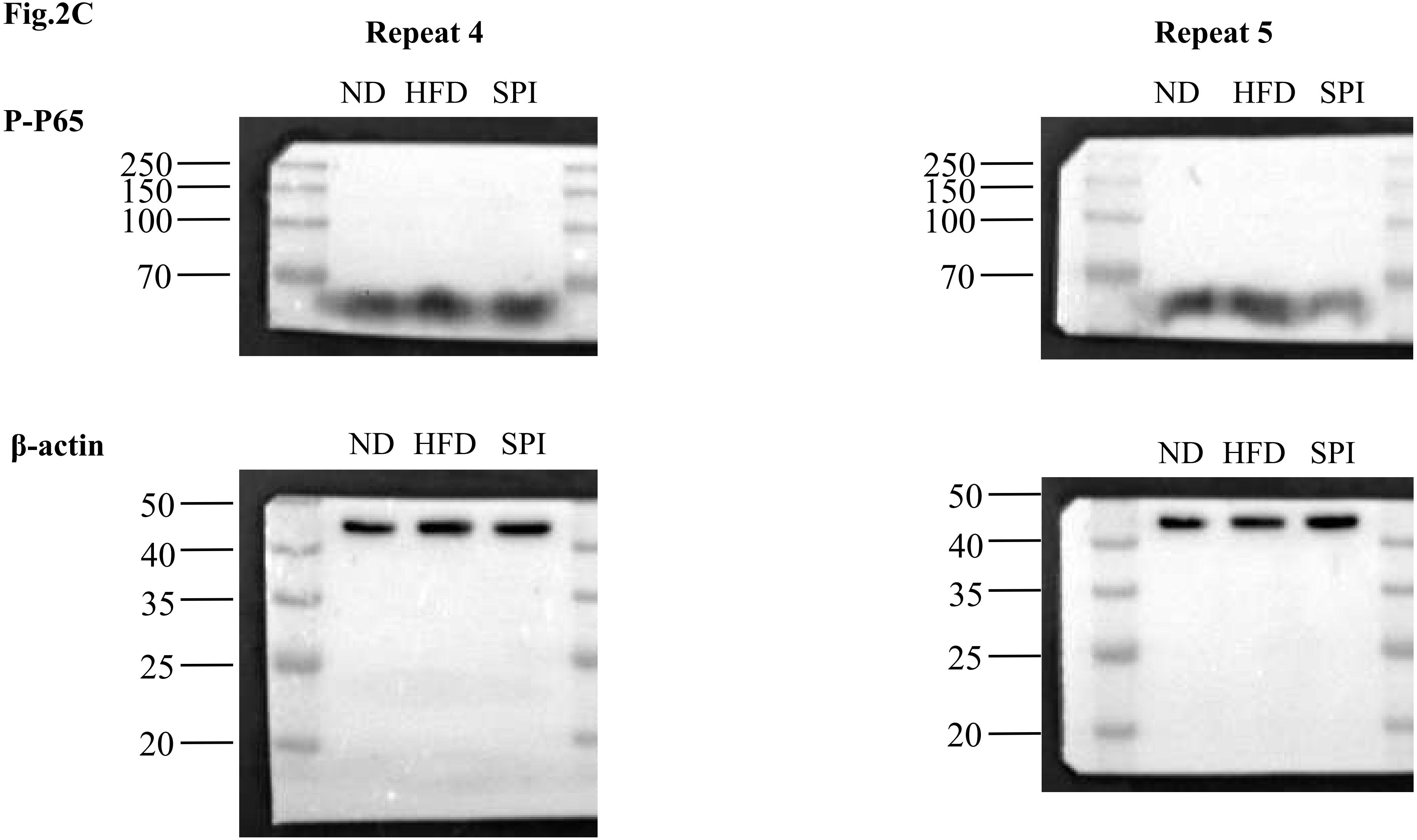
Repeat 4
Repeat 5
P-P65
ND HFD SPI
250
150
100
70
ND HFD SPI
250
150
100
70
β-actin
ND HFD SPI
50
40
35
25
20
ND HFD SPI
50
40
35
25
20

Fig.2C

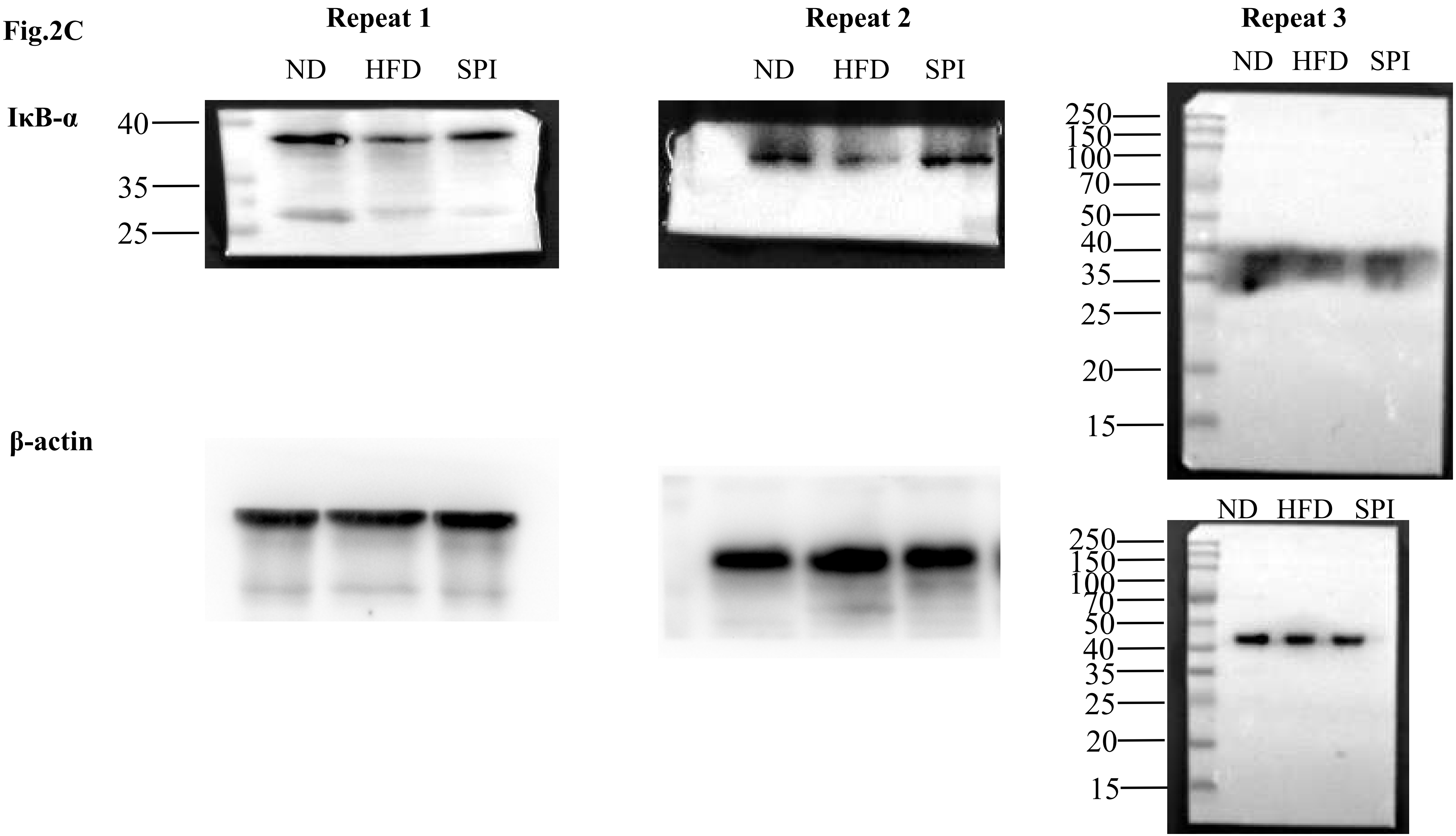
Repeat 1
Repeat 2
Repeat 3
IκB-α
β-actin
ND HFD SPI
ND HFD SPI
ND HFD SPI
ND HFD SPI
40
35
25
250
150
100
70
50
40
35
25
20
15
250
150
100
70
50
40
35
25
20
15

Fig.2C

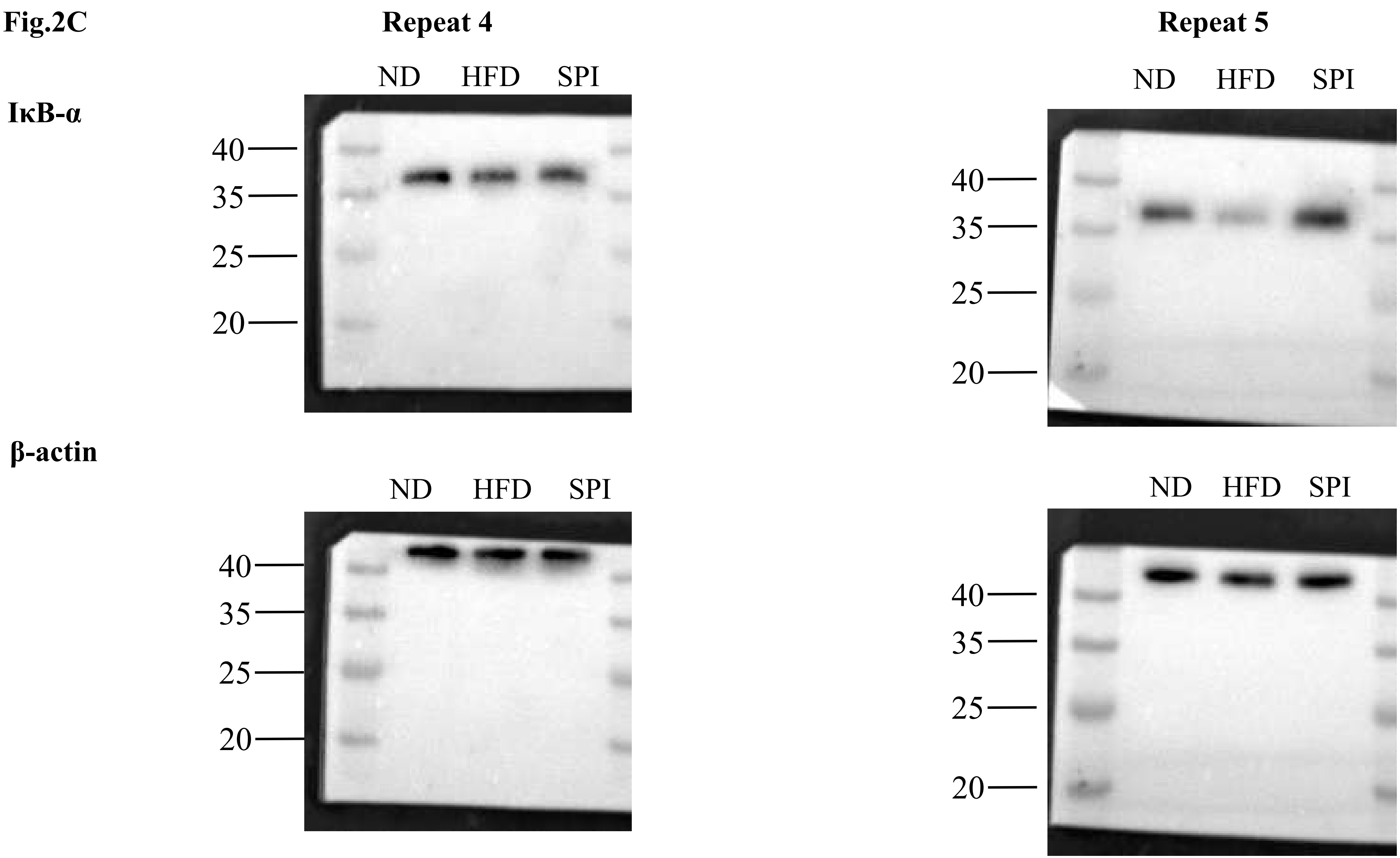
Repeat 4
Repeat 5
IκB-α
ND HFD SPI
40
35
25
20
ND HFD SPI
40
35
25
20
β-actin
ND HFD SPI
40
35
25
20
ND HFD SPI
40
35
25
20

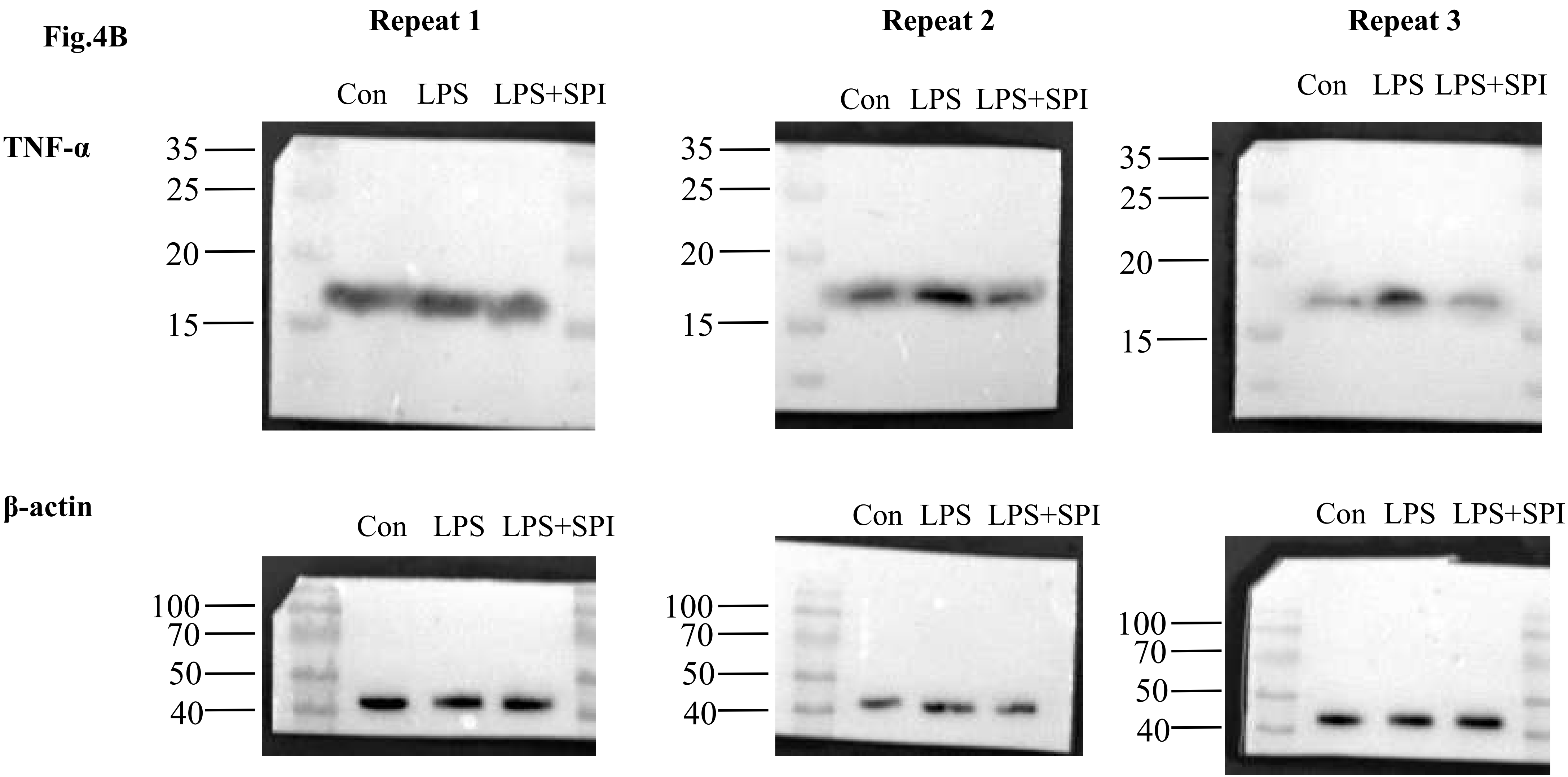
Fig.4B
Repeat 1
Repeat 2
Repeat 3
TNF-α
β-actin
Con LPS LPS+SPI
35
25
20
15
100
70
50
40

Fig.4B

Repeat 4

Repeat 5

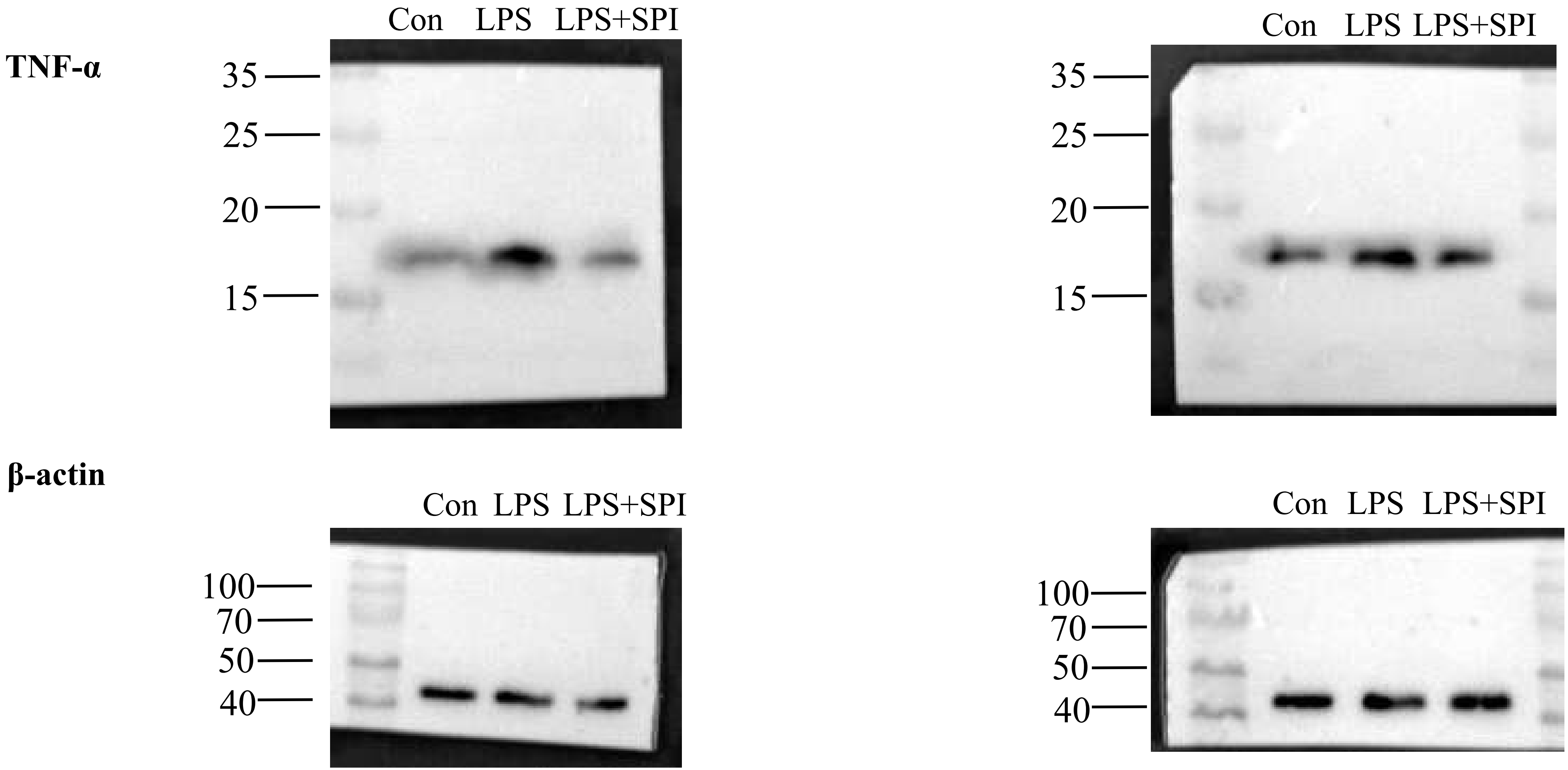

Fig.4B

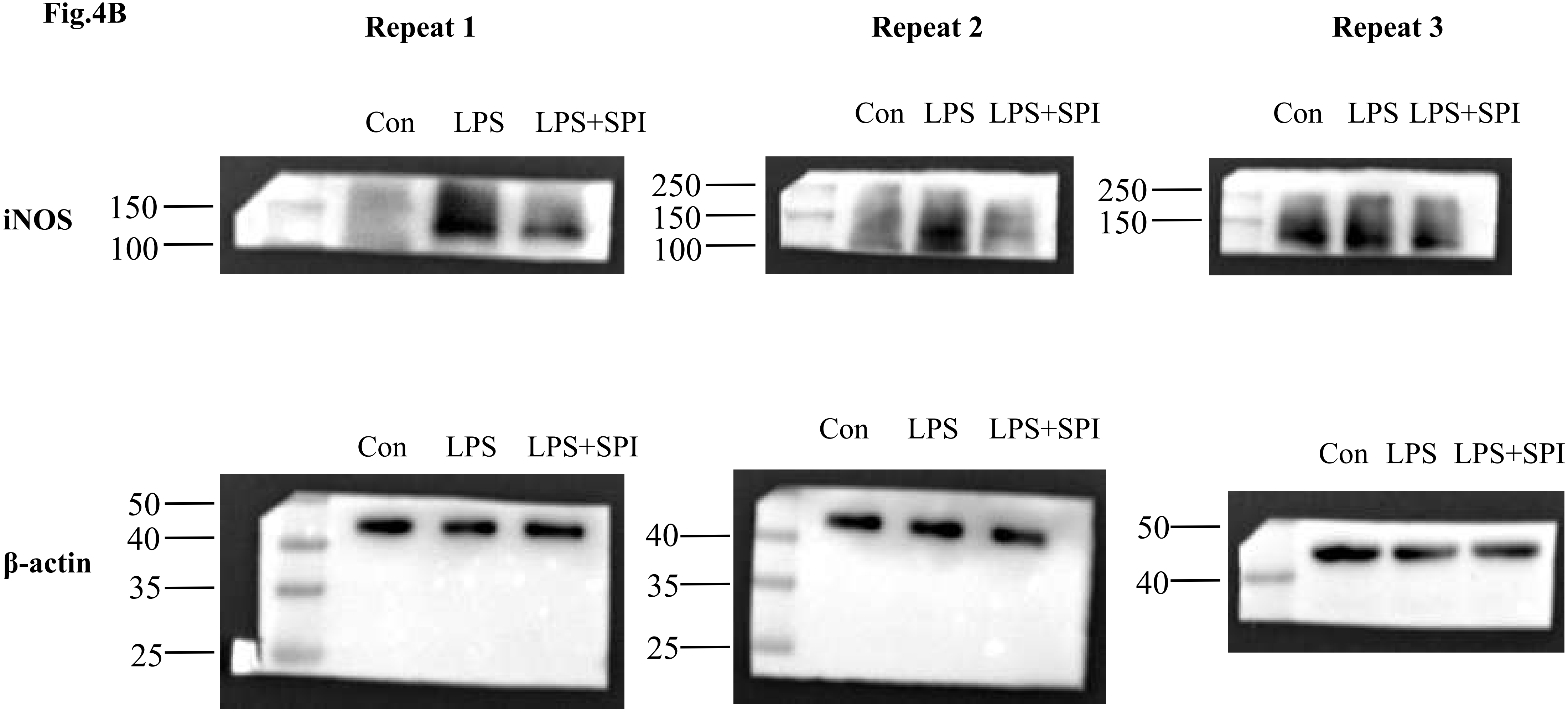
Repeat 1
Repeat 2
Repeat 3
Con
LPS
LPS+SPI
iNOS
250
150
100
β-actin
50
40
35
25

Fig.4B

Repeat 4 Repeat 5

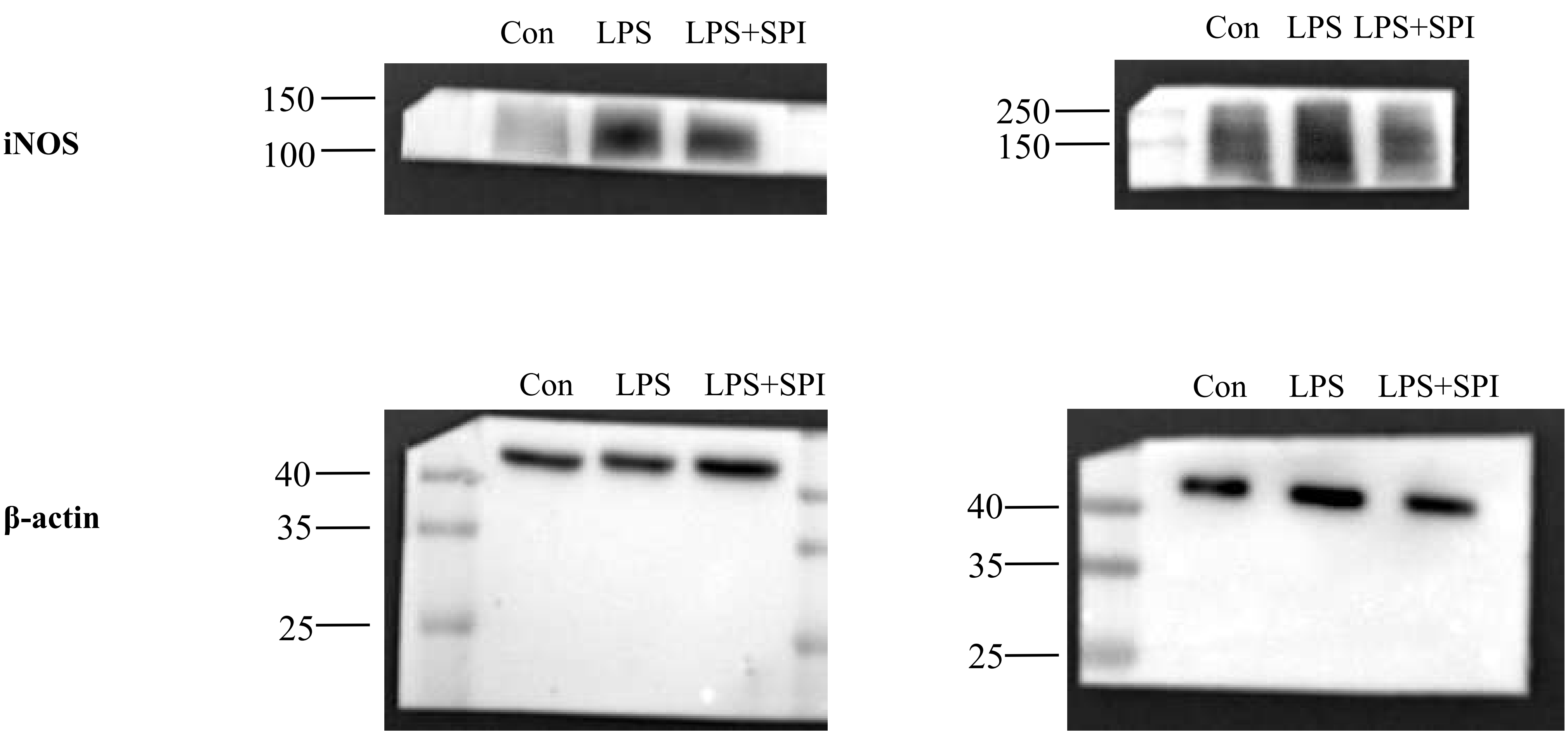

Fig.4B

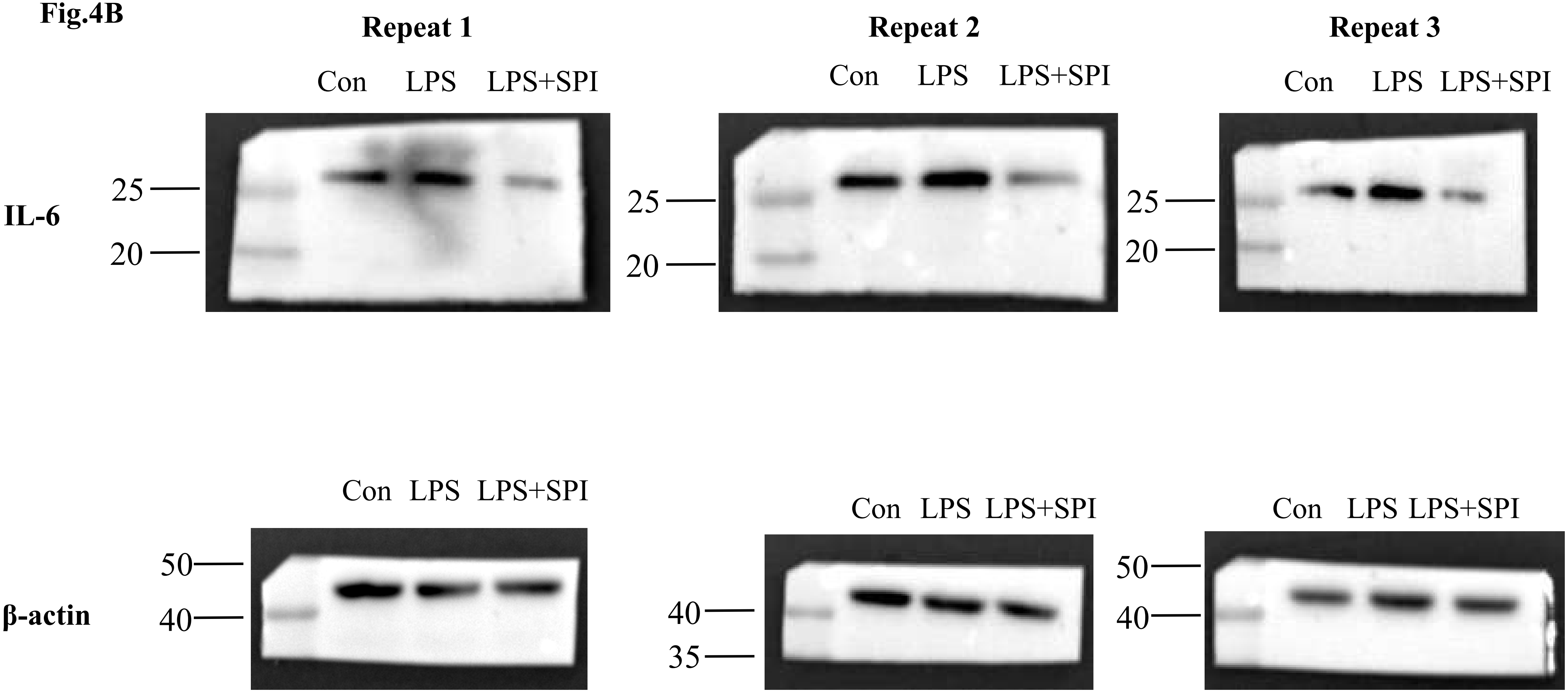

Repeat 1
Repeat 2
Repeat 3
Con
LPS
LPS+SPI
IL-6
25
20
β-actin
50
40
35

Fig.4B

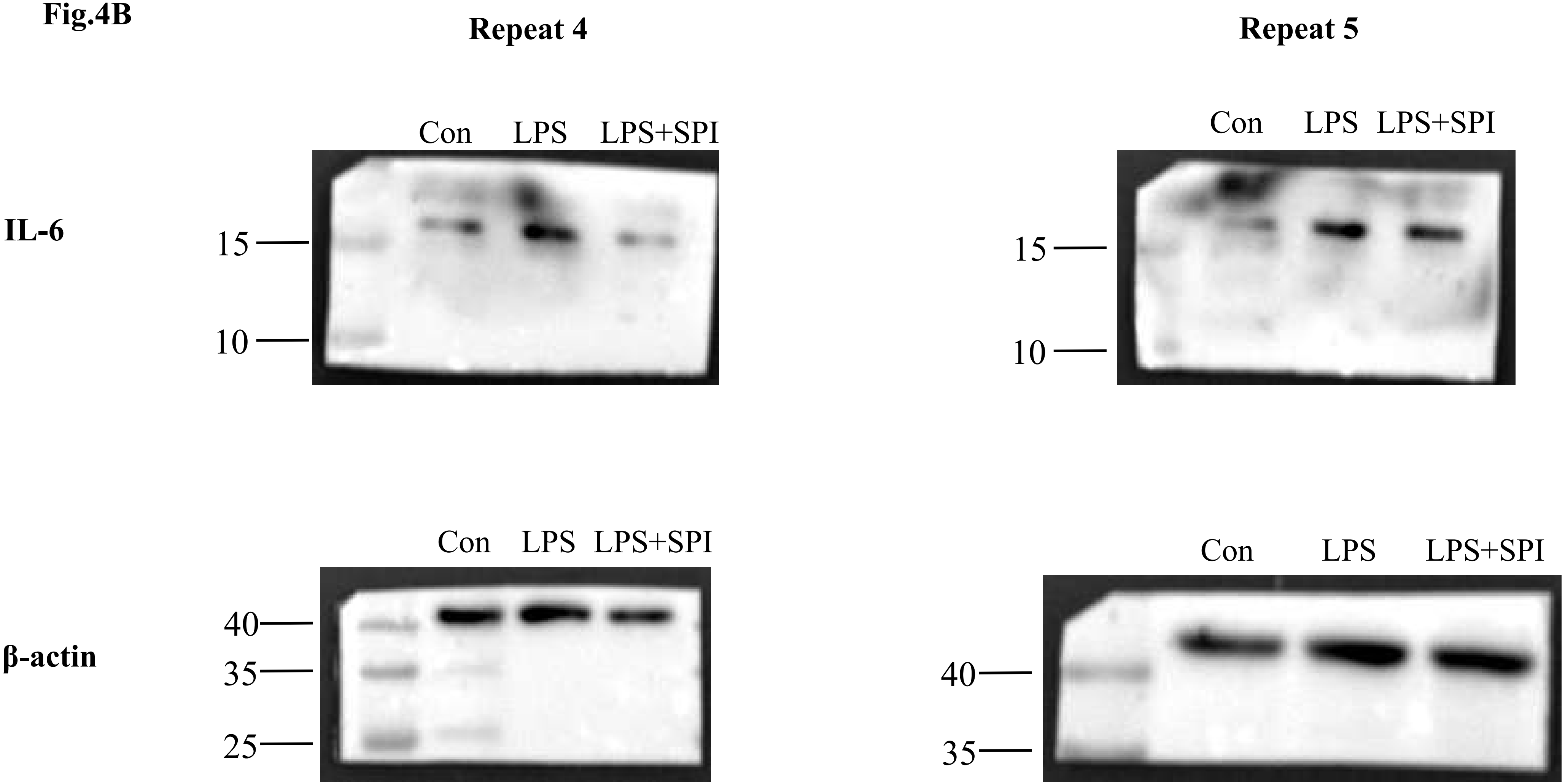
Repeat 4
Repeat 5
IL-6
Con
LPS
LPS+SPI
15
10
Con
LPS
LPS+SPI
15
10
β-actin
Con
LPS
LPS+SPI
40
35
25
Con
LPS
LPS+SPI
40
35

Fig.4B

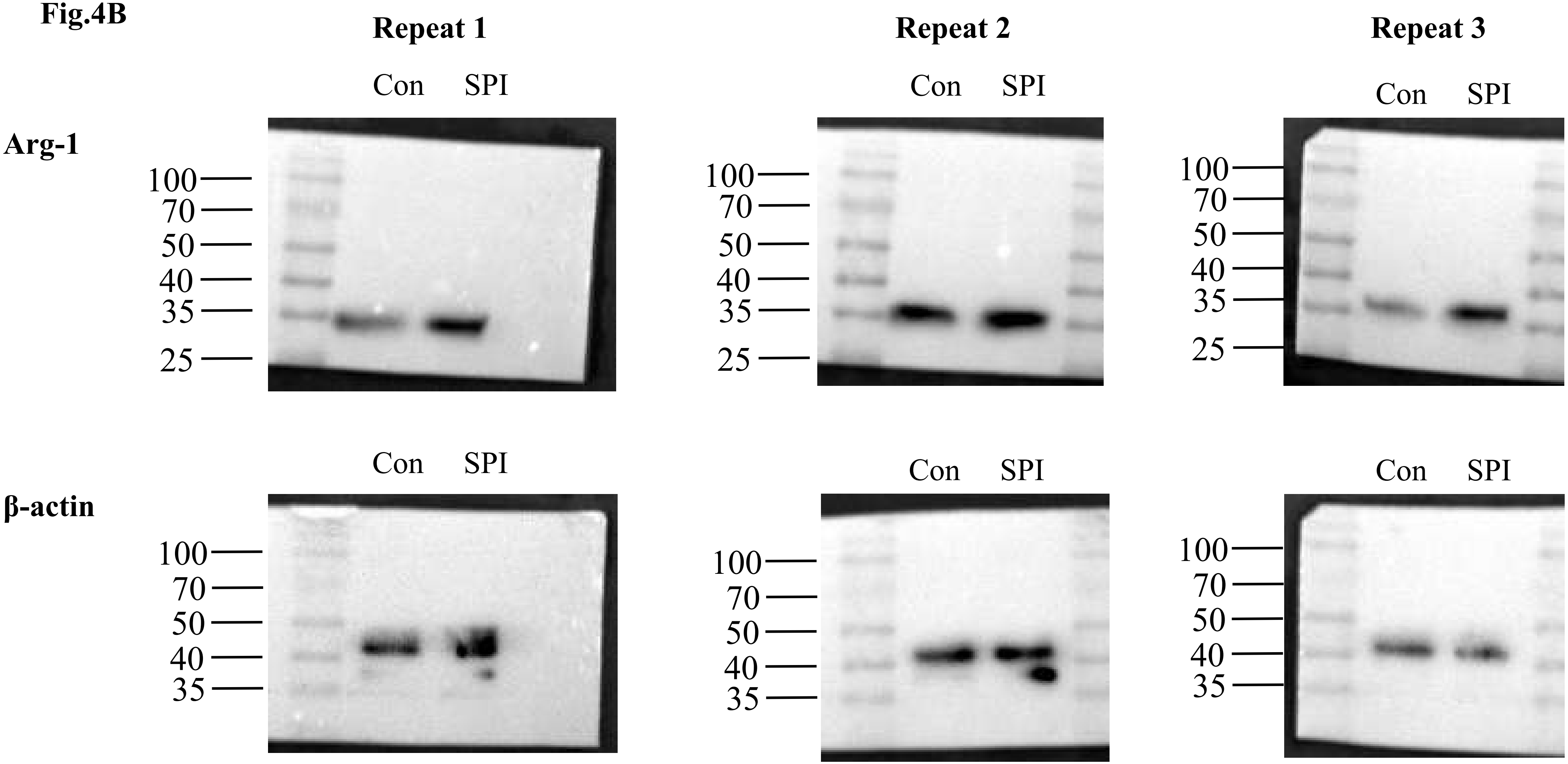
Repeat 1
Repeat 2
Repeat 3
Arg-1
β-actin
Con
SPI
100
70
50
40
35
25

Fig.4B

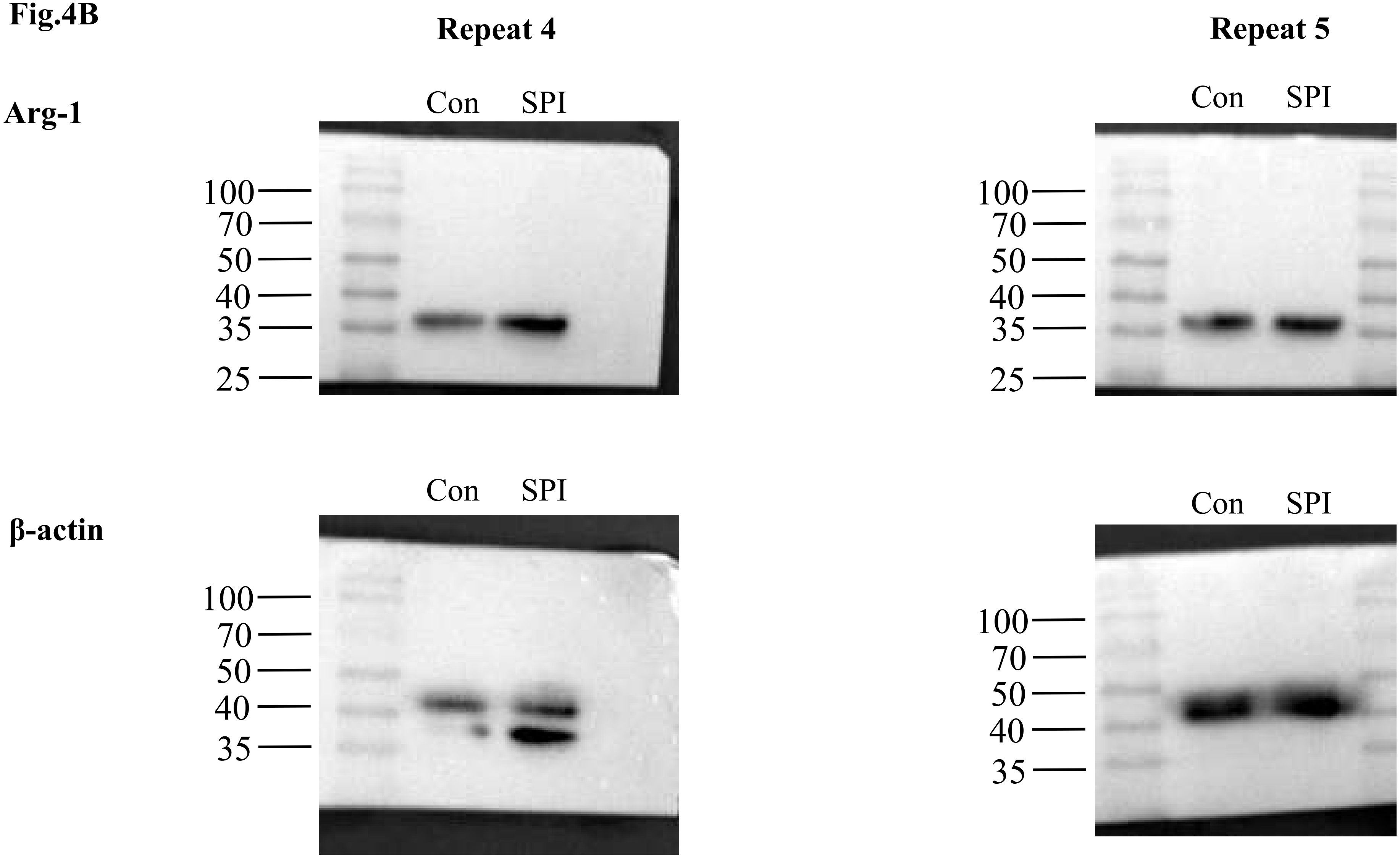

Fig.4B

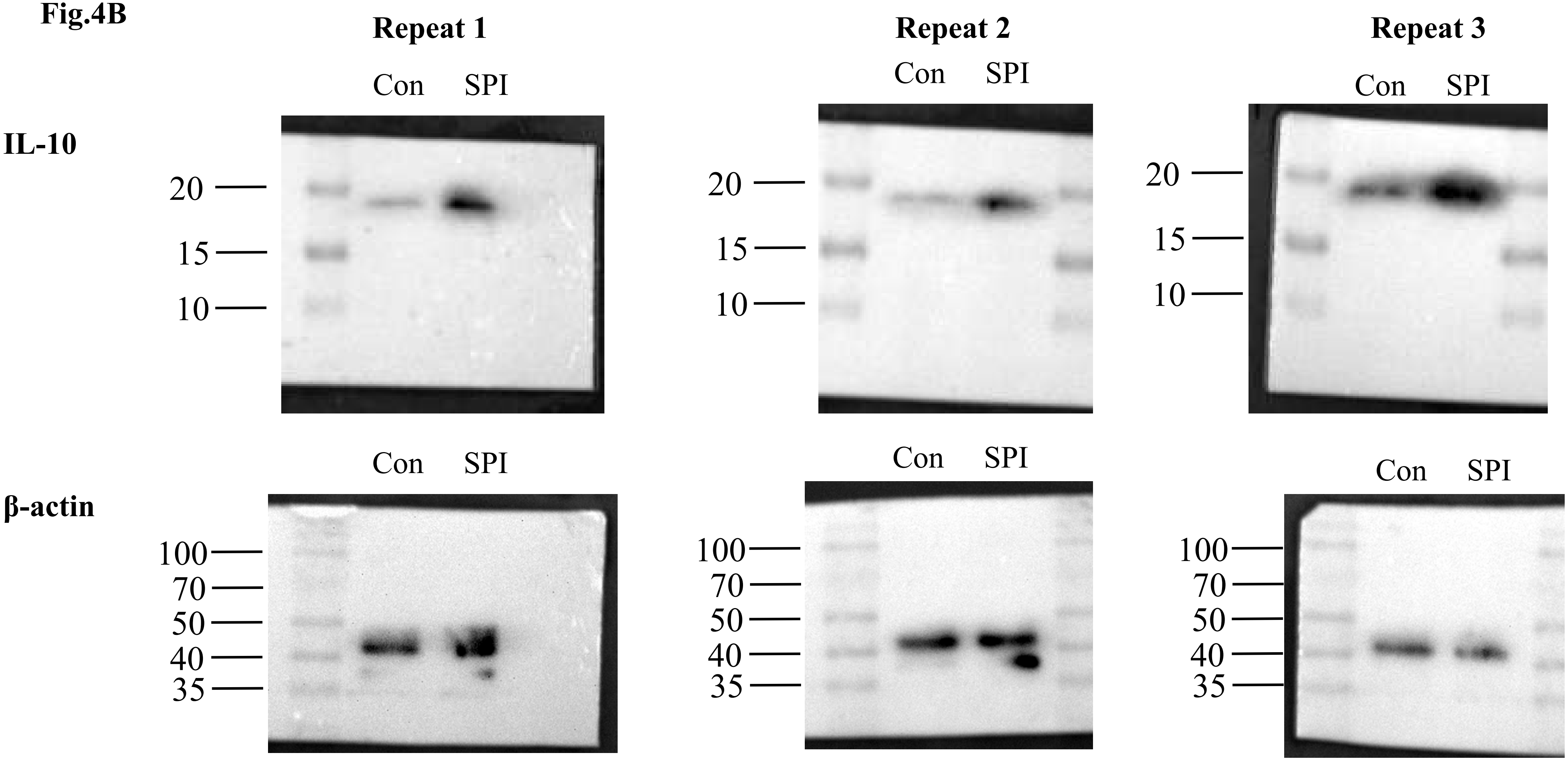

Repeat 1
Repeat 2
Repeat 3
Con
SPI
IL-10
20
15
10
β-actin
100
70
50
40
35

Fig.4B

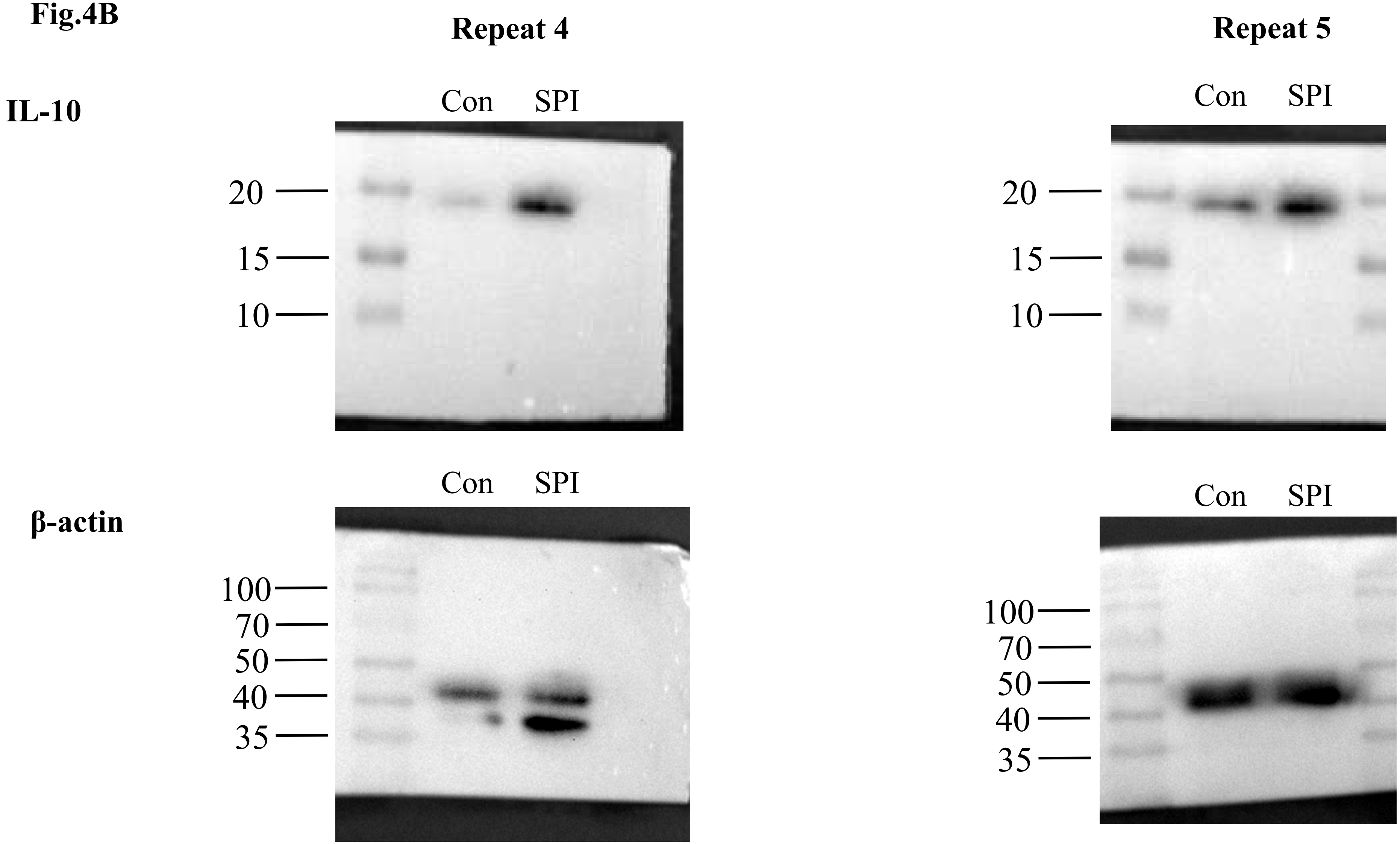

Repeat 4
Repeat 5
IL-10
Con
SPI
20
15
10
Con
SPI
20
15
10
β-actin
Con
SPI
100
70
50
40
35
Con
SPI
100
70
50
40
35

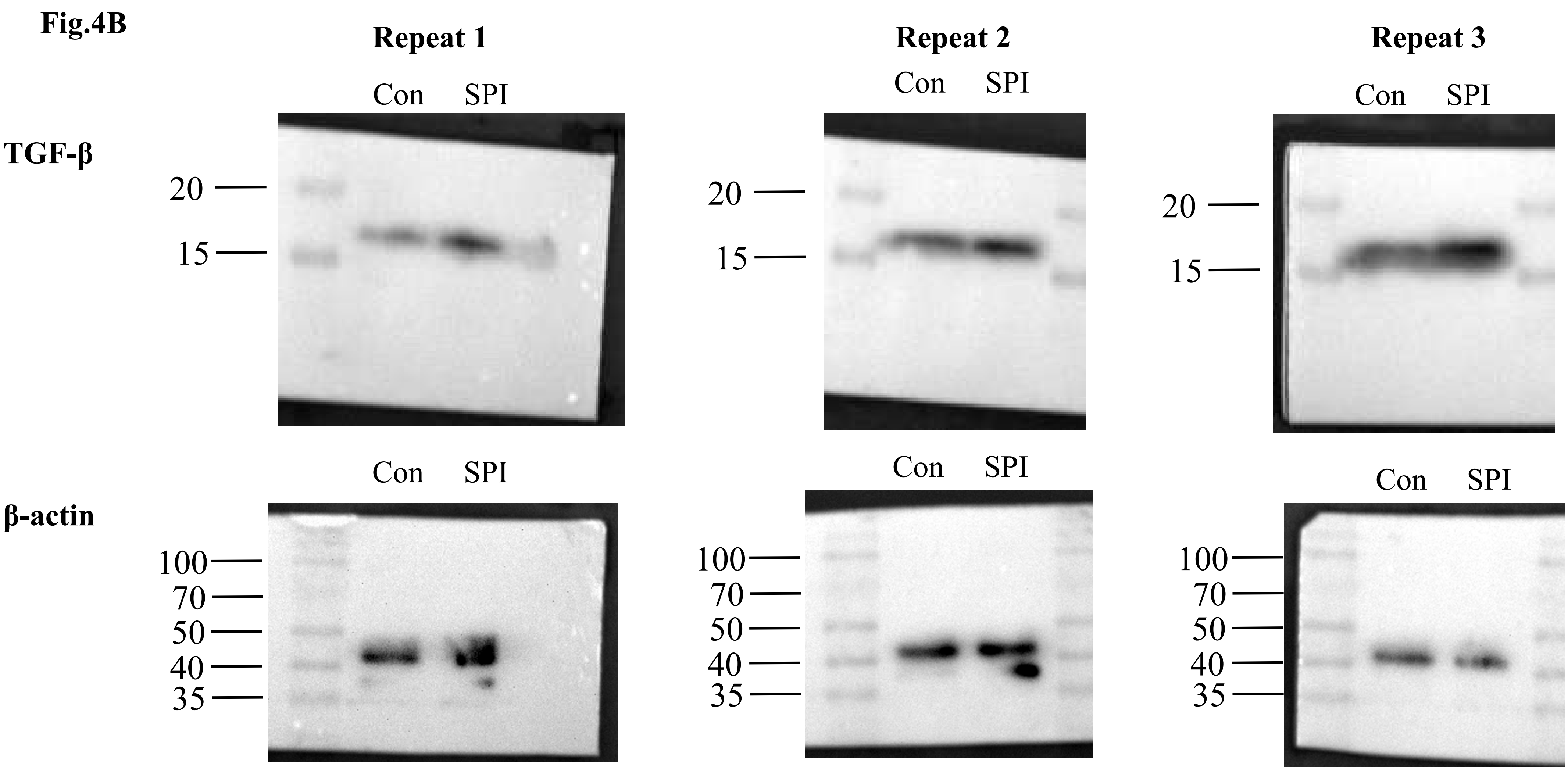
Fig.4B
Repeat 1
Repeat 2
Repeat 3
TGF-β
β-actin
Con
SPI
20
15
100
70
50
40
35

Fig.4B

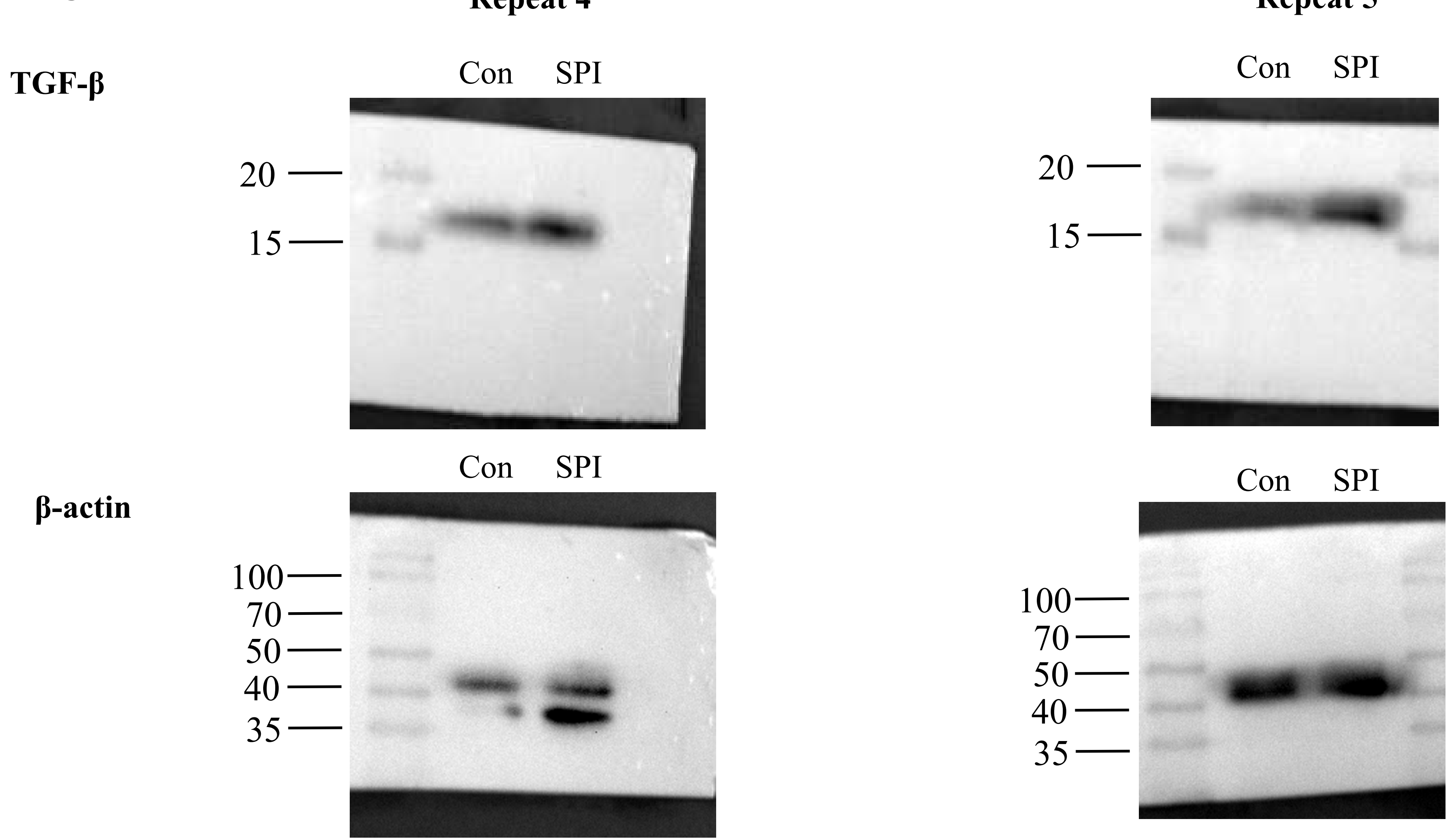
Repeat 4
Repeat 5
TGF-β
Con
SPI
20
15
Con
SPI
20
15
β-actin
Con
SPI
100
70
50
40
35
Con
SPI
100
70
50
40
35

Fig.6G

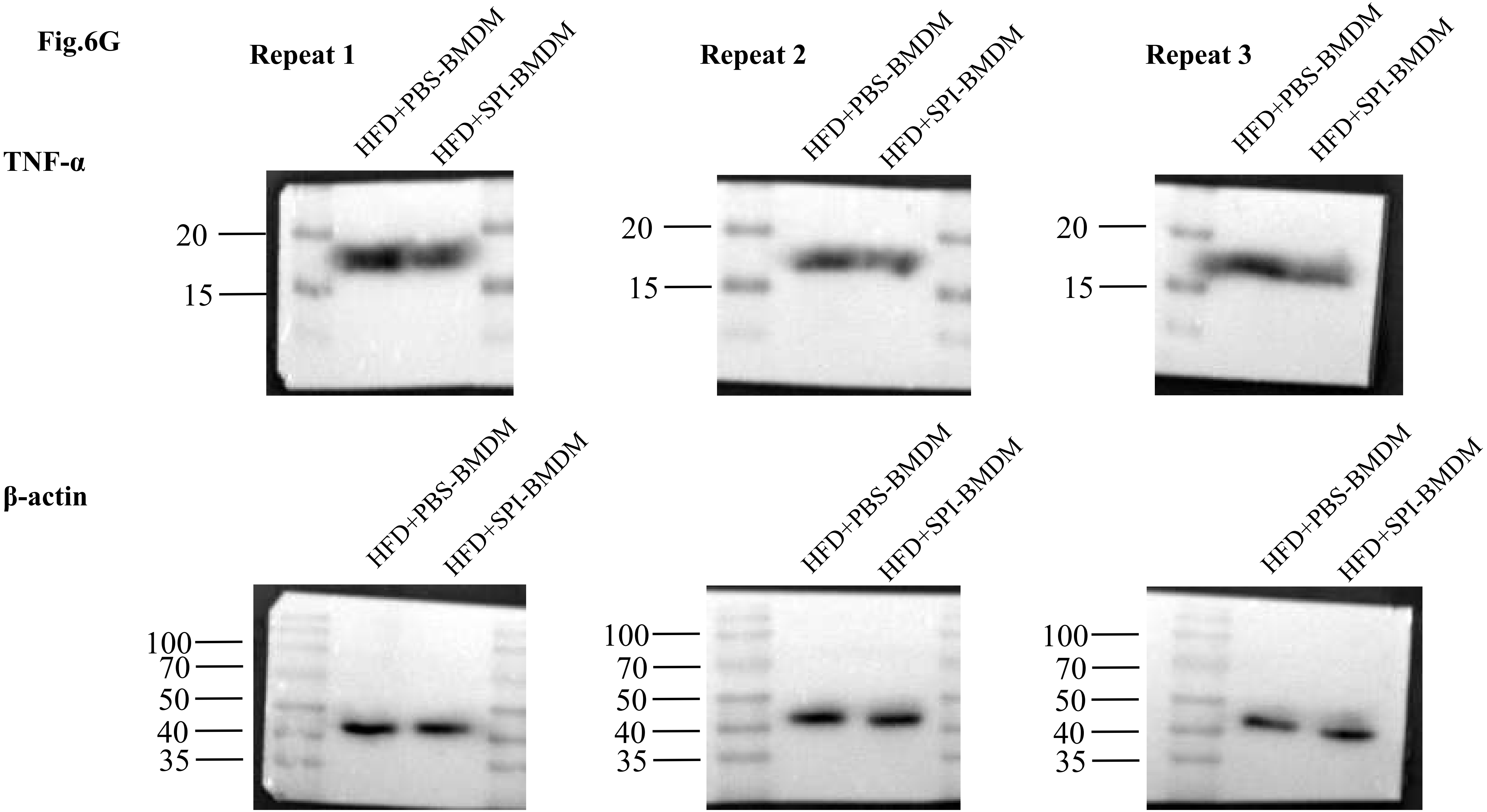
Repeat 1
Repeat 2
Repeat 3
TNF-α
β-actin
HFD+PBS-BMDM
HFD+SPI-BMDM
20
15
100
70
50
40
35

Fig.6G

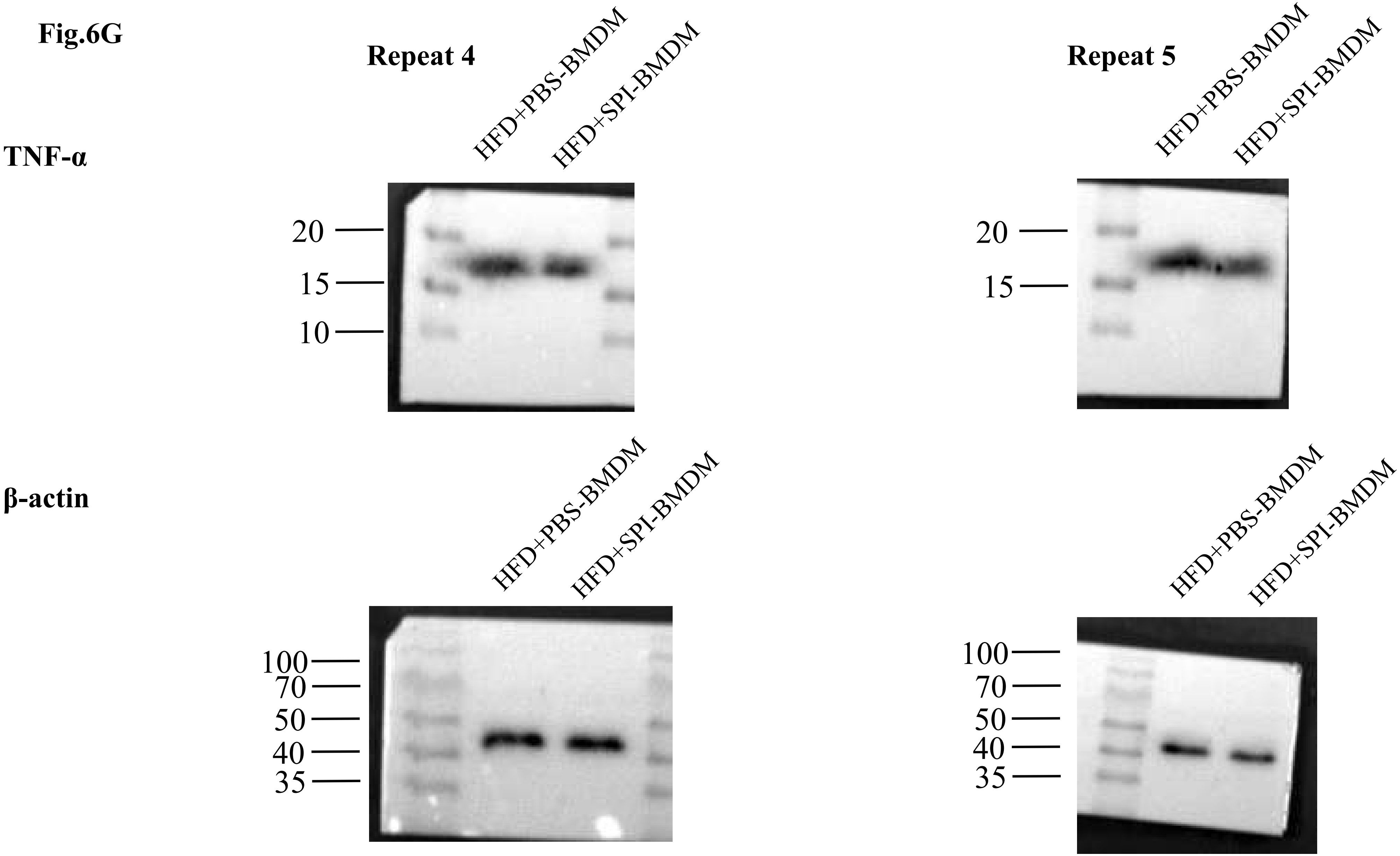

Repeat 4
Repeat 5
TNF-α
β-actin
HFD+PBS-BMDM
HFD+SPI-BMDM
HFD+PBS-BMDM
HFD+SPI-BMDM
HFD+PBS-BMDM
HFD+SPI-BMDM
HFD+PBS-BMDM
HFD+SPI-BMDM
20
15
10
20
15
100
70
50
40
35
100
70
50
40
35

Fig.6G

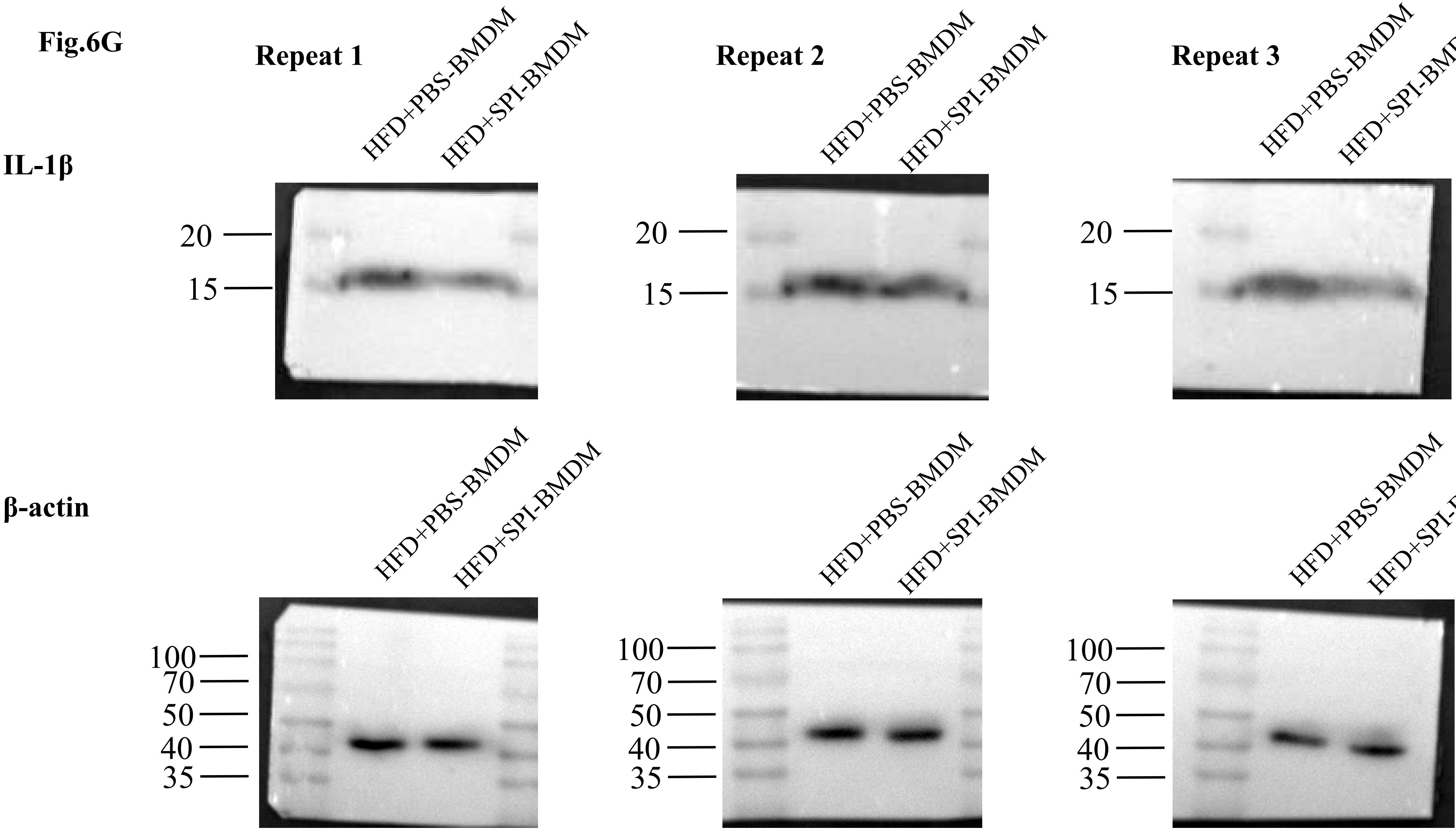

Repeat 1
Repeat 2
Repeat 3
IL-1β
β-actin
HFD+PBS-BMDM
HFD+SPI-BMDM
20
15
100
70
50
40
35

Fig.6G

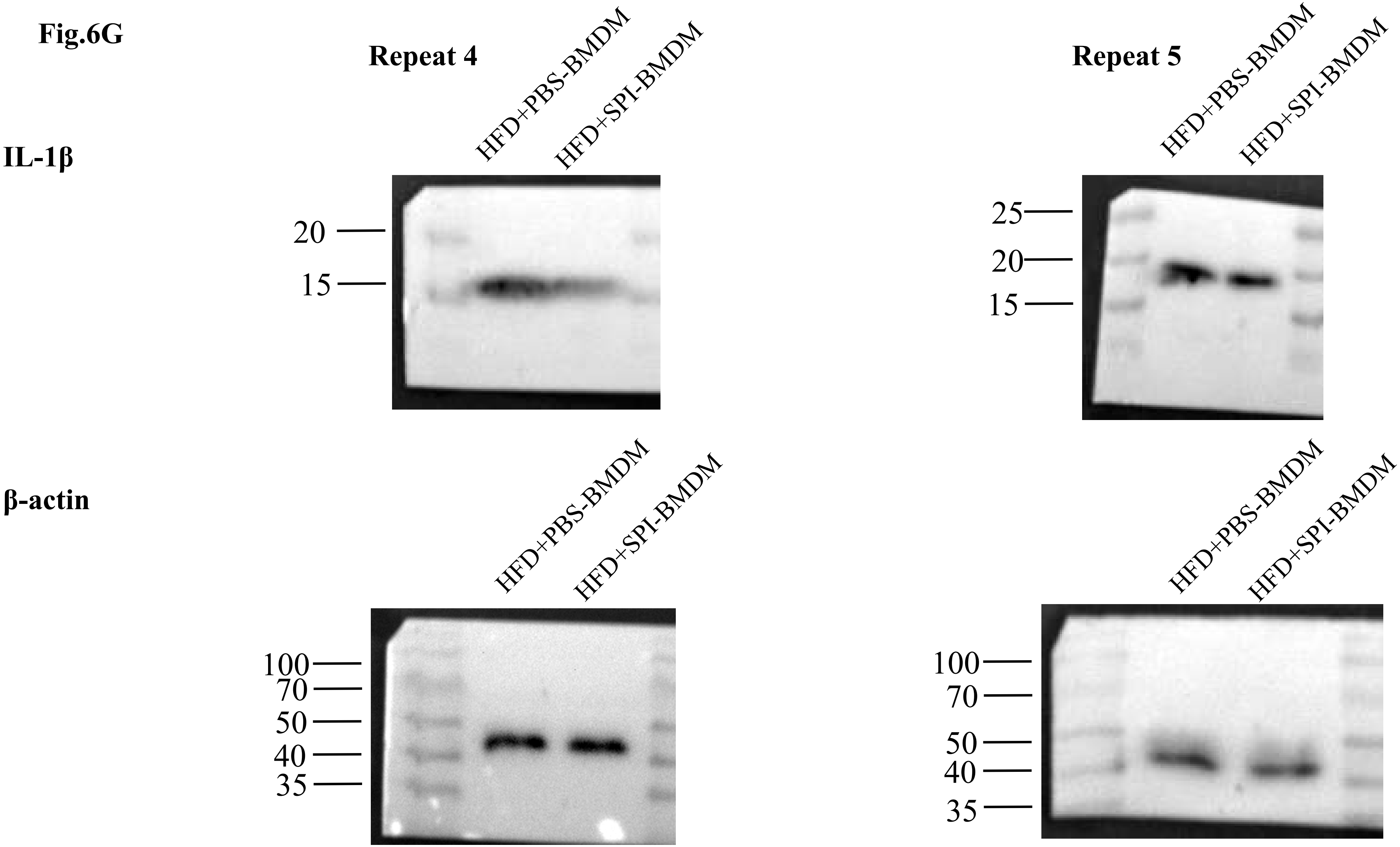
Repeat 4
Repeat 5
IL-1β
β-actin
HFD+PBS-BMDM
HFD+SPI-BMDM
20
15
25
20
15
100
70
50
40
35
100
70
50
40
35

Fig.6G

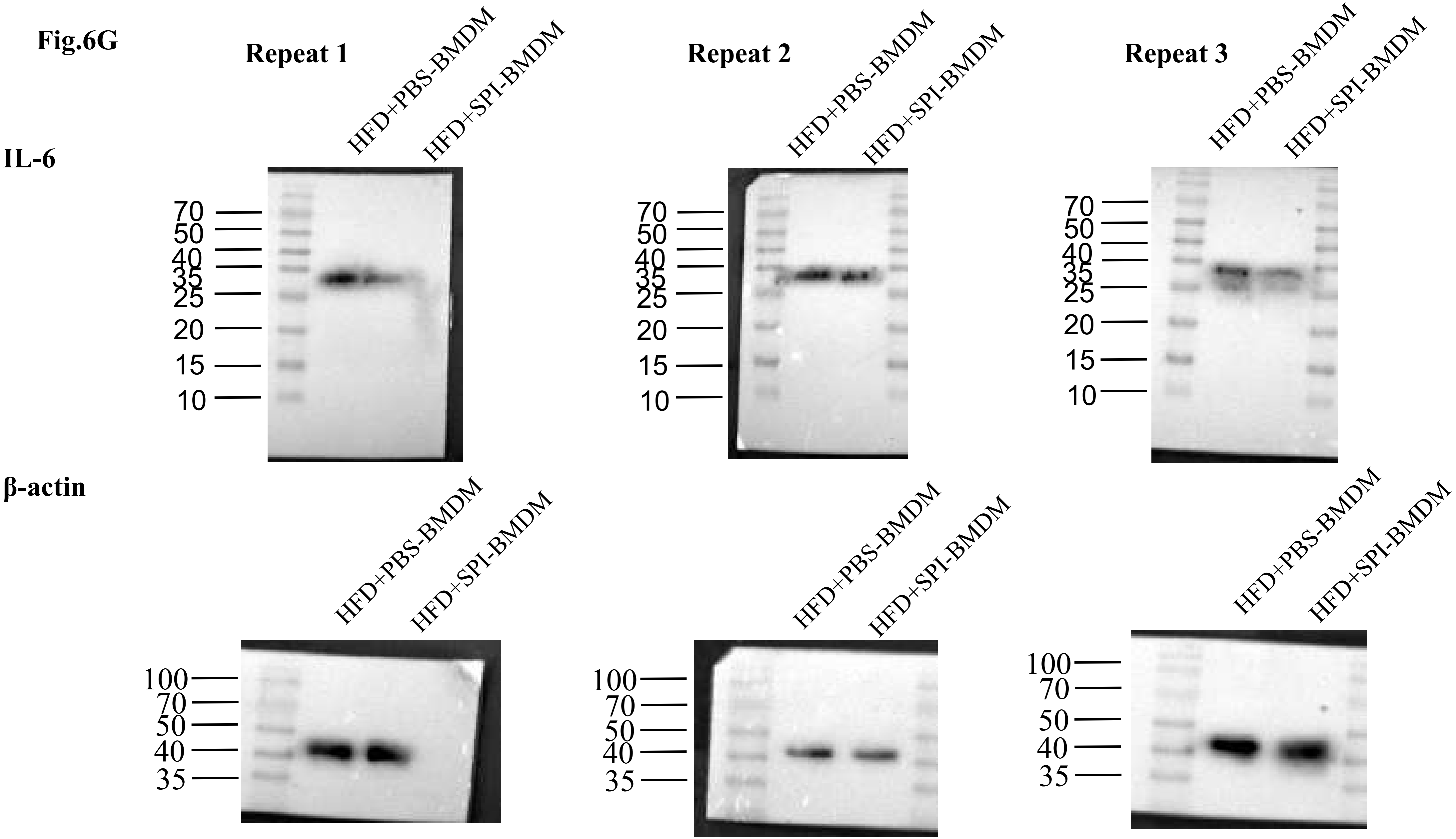
Repeat 1
Repeat 2
Repeat 3
IL-6
β-actin
HFD+PBS-BMDM
HFD+SPI-BMDM
70
50
40
35
25
20
15
10
100
70
50
40
35

Fig.6G

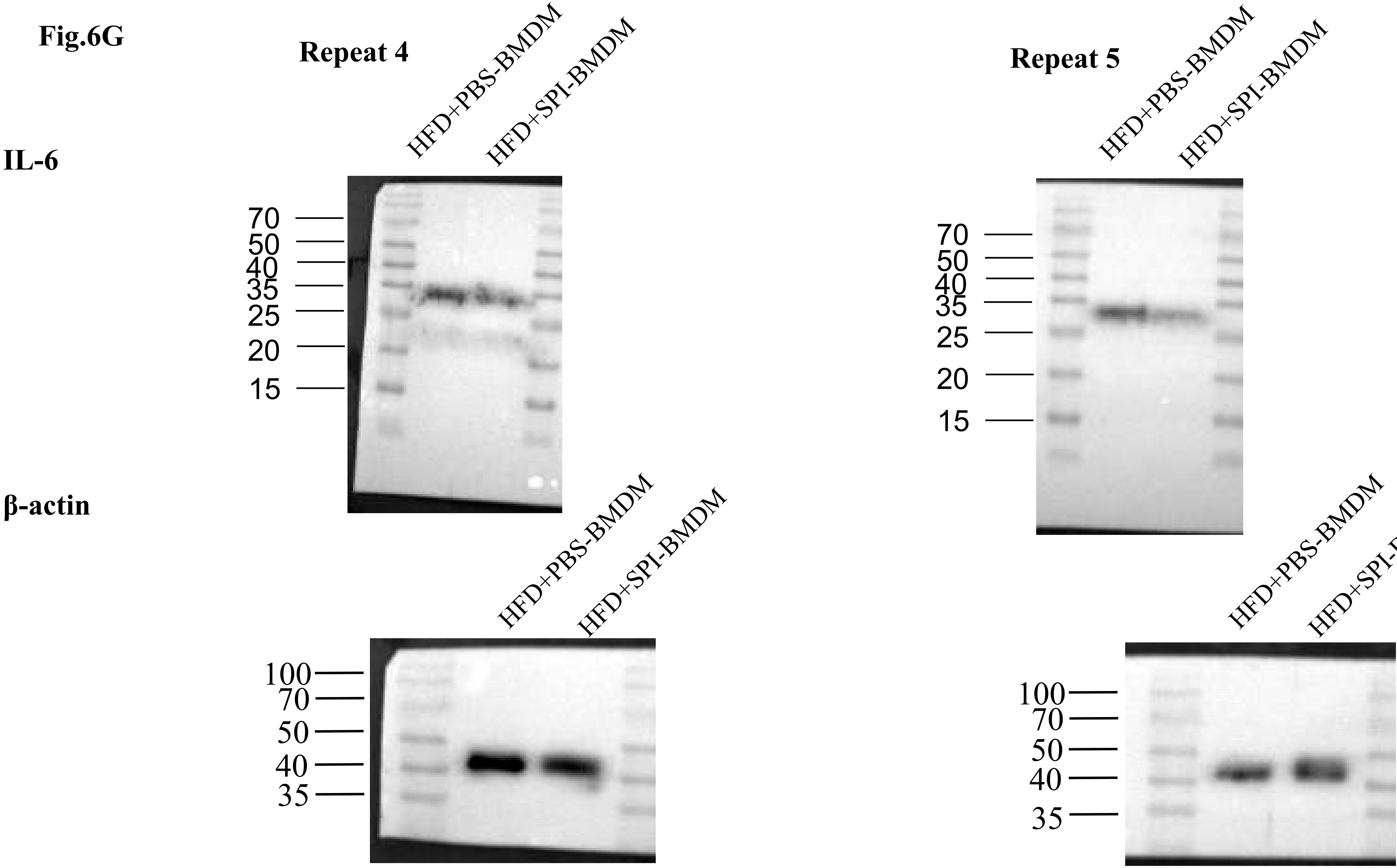

Repeat 4
Repeat 5
IL-6
β-actin
HFD+PBS-BMDM
HFD+SPI-BMDM
HFD+PBS-BMDM
HFD+SPI-BMDM
HFD+PBS-BMDM
HFD+SPI-BMDM
HFD+PBS-BMDM
HFD+SPI-BMDM
70
50
40
35
25
20
15
70
50
40
35
25
20
15
100
70
50
40
35
100
70
50
40
35

Fig.6G

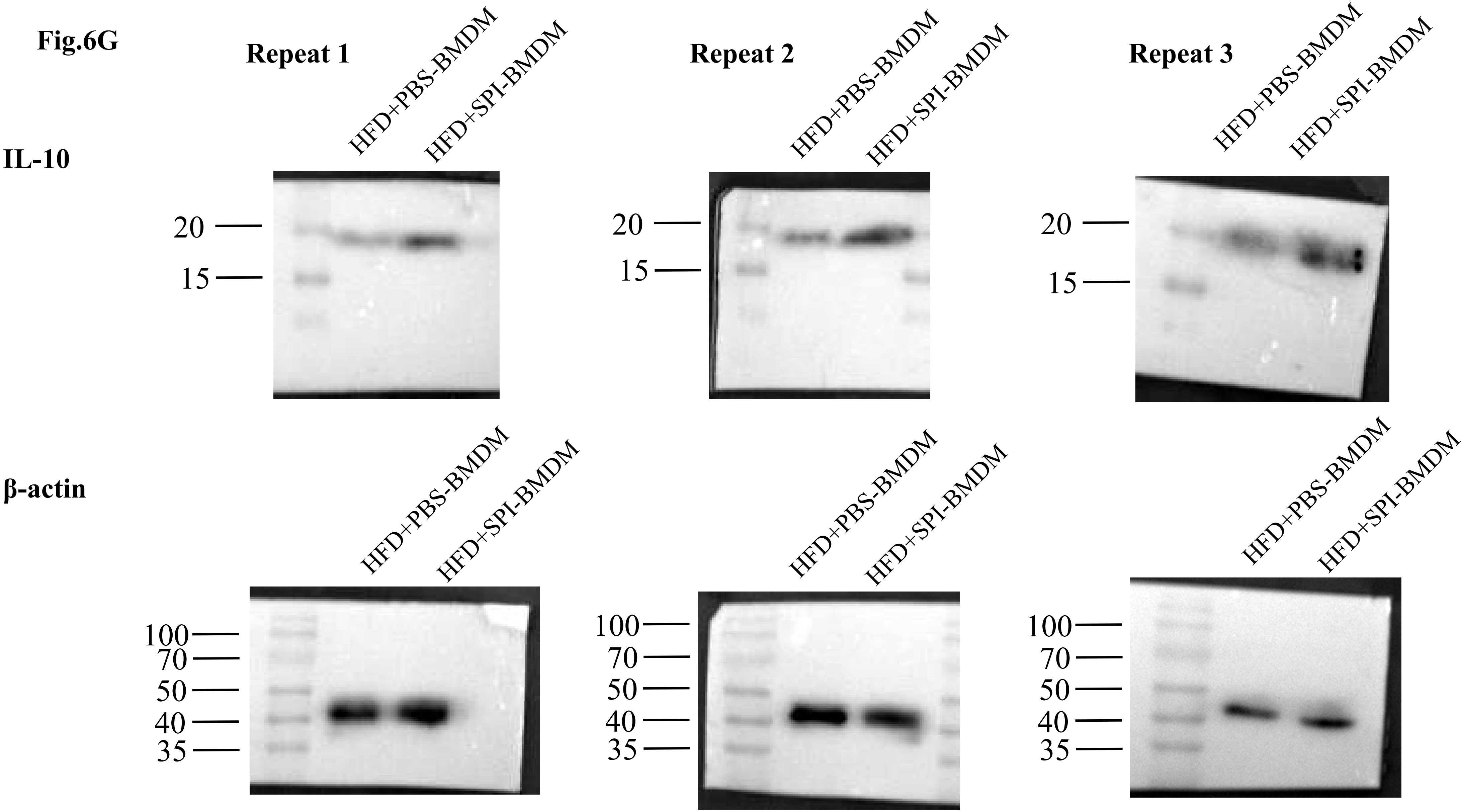
Repeat 1
Repeat 2
Repeat 3
IL-10
HFD+PBS-BMDM
HFD+SPI-BMDM
20
15
β-actin
100
70
50
40
35

Fig.6G

IL-10

β-actin

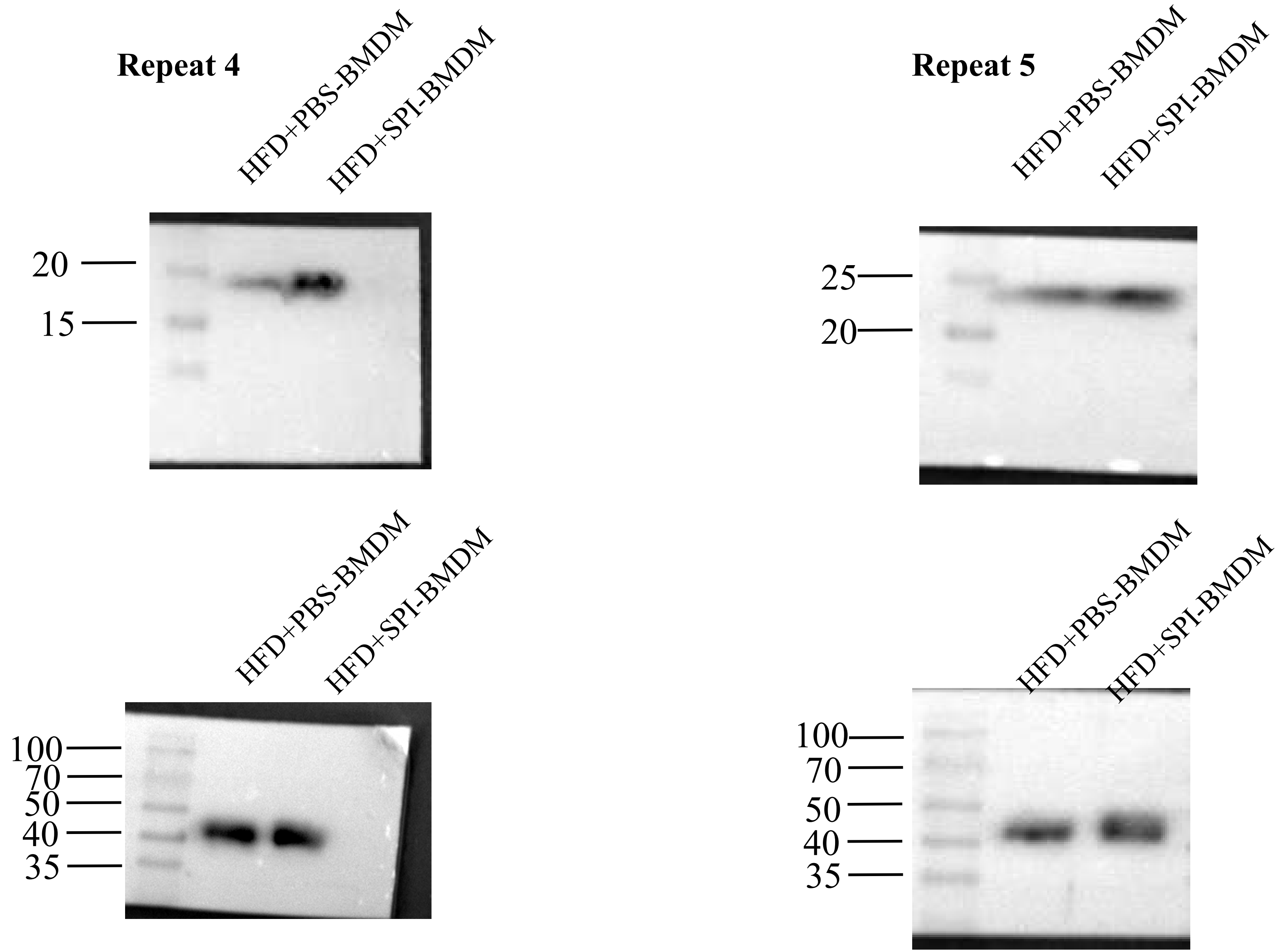

Fig.6H

P-P65

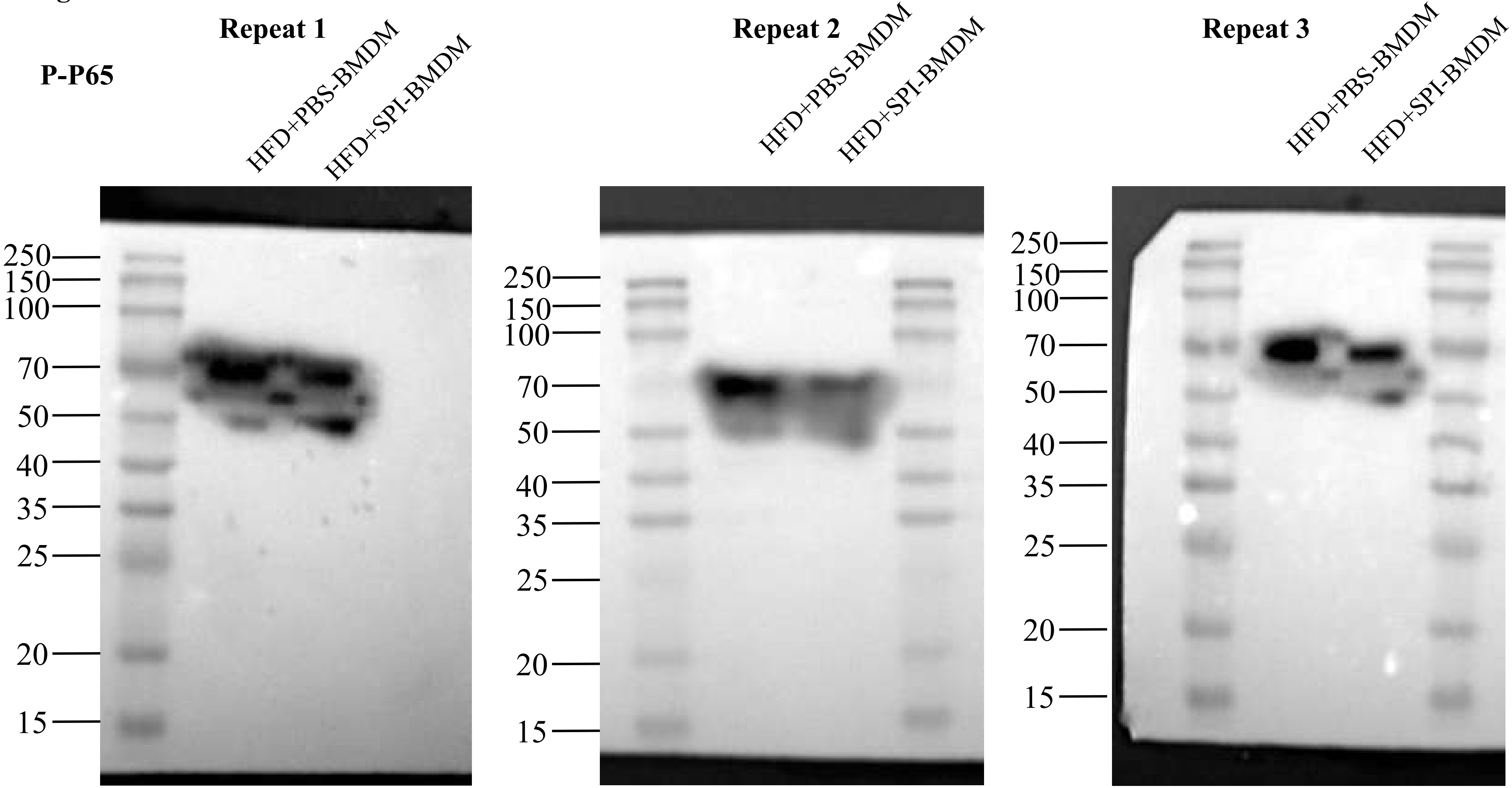
Repeat 1
HFD+PBS-BMDM
HFD+SPI-BMDM
250
150
100
70
50
40
35
25
20
15
Repeat 2
HFD+PBS-BMDM
HFD+SPI-BMDM
250
150
100
70
50
40
35
25
20
15
Repeat 3
HFD+PBS-BMDM
HFD+SPI-BMDM
250
150
100
70
50
40
35
25
20
15

Fig.6H

β-actin

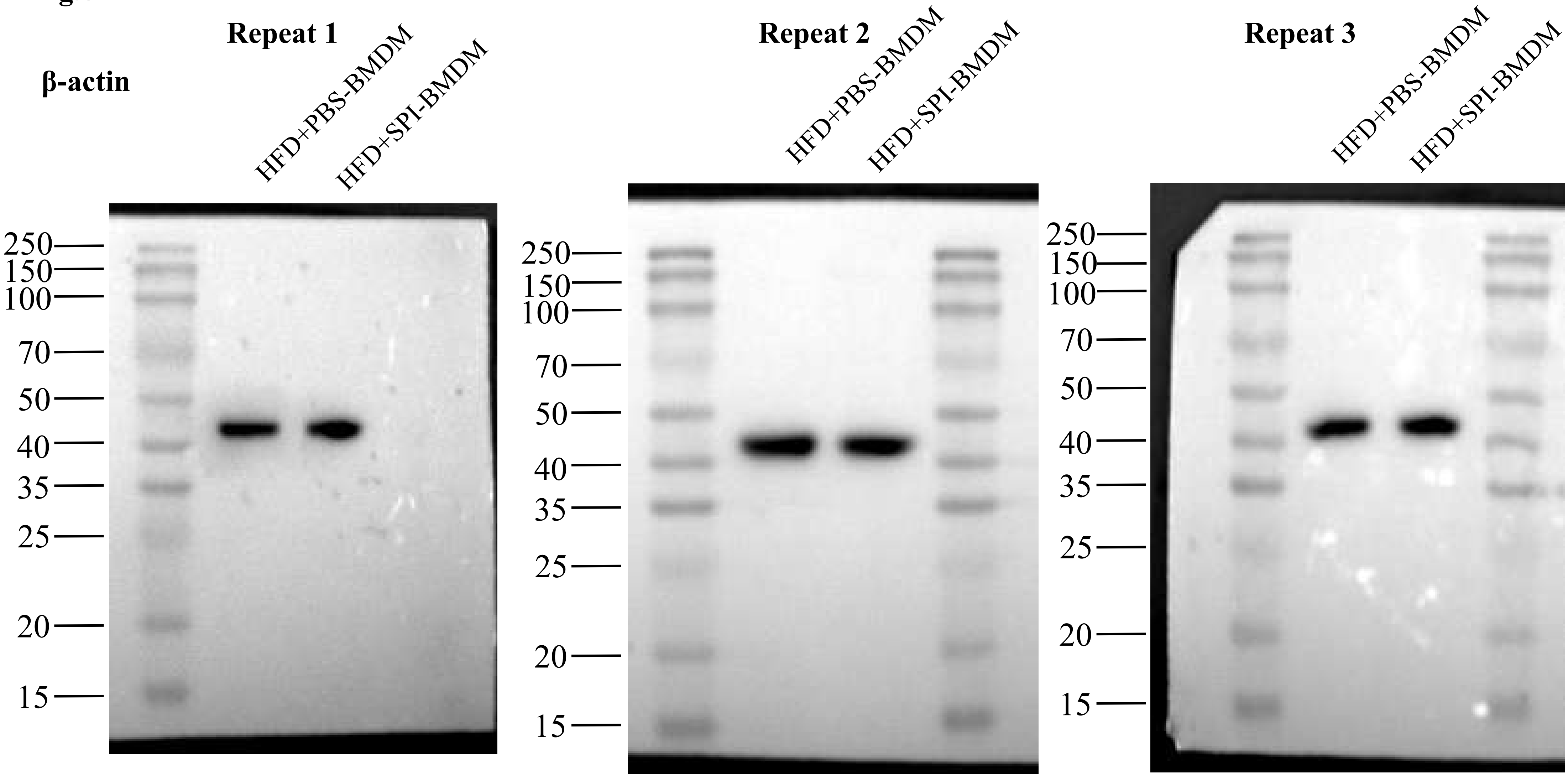

Fig.6H

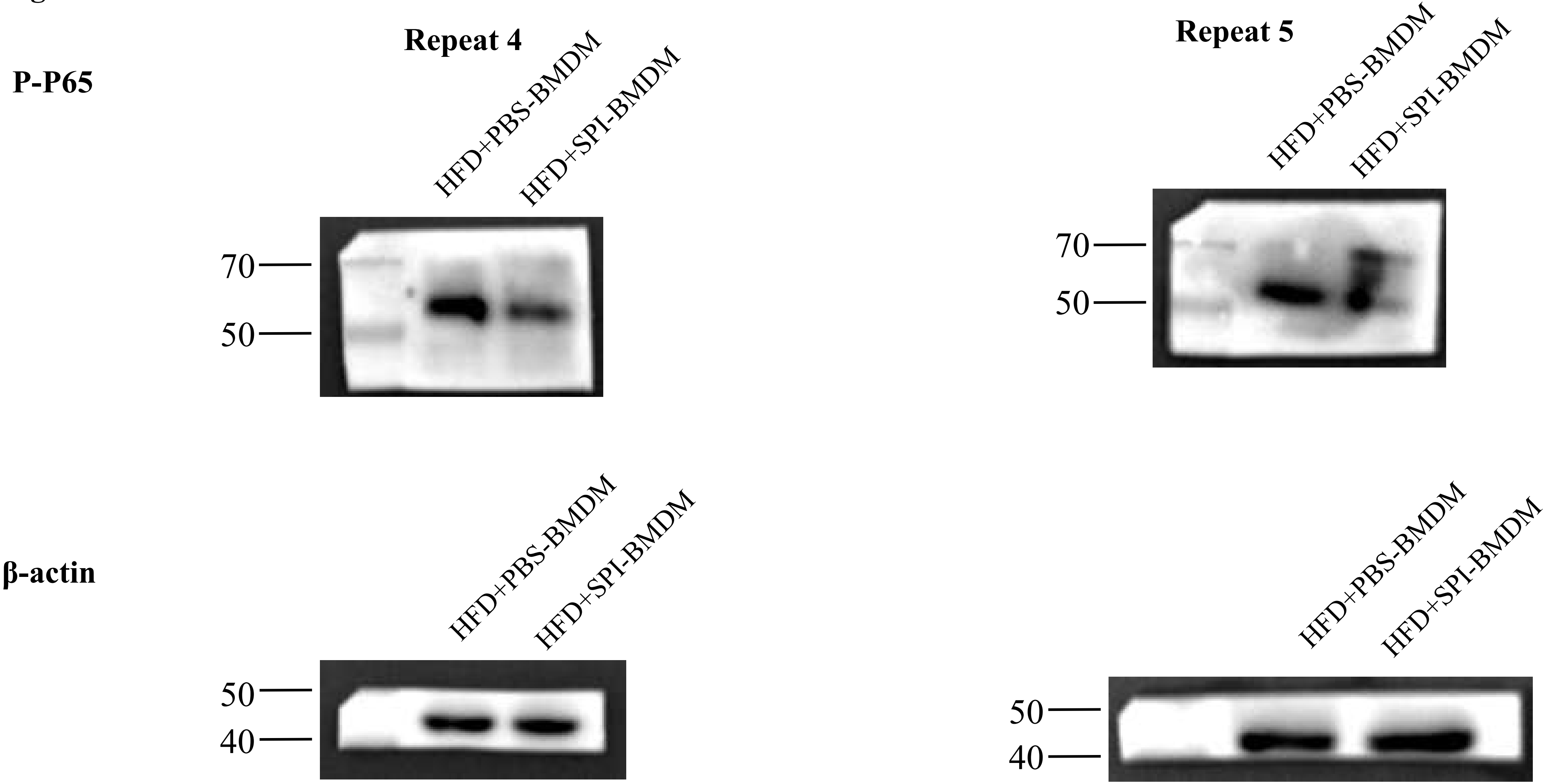
Repeat 4
Repeat 5
P-P65
β-actin
HFD+PBS-BMDM
HFD+SPI-BMDM
HFD+PBS-BMDM
HFD+SPI-BMDM
HFD+PBS-BMDM
HFD+SPI-BMDM
HFD+PBS-BMDM
HFD+SPI-BMDM
70
50
70
50
50
40
50
40

Fig.6H

IκB-α

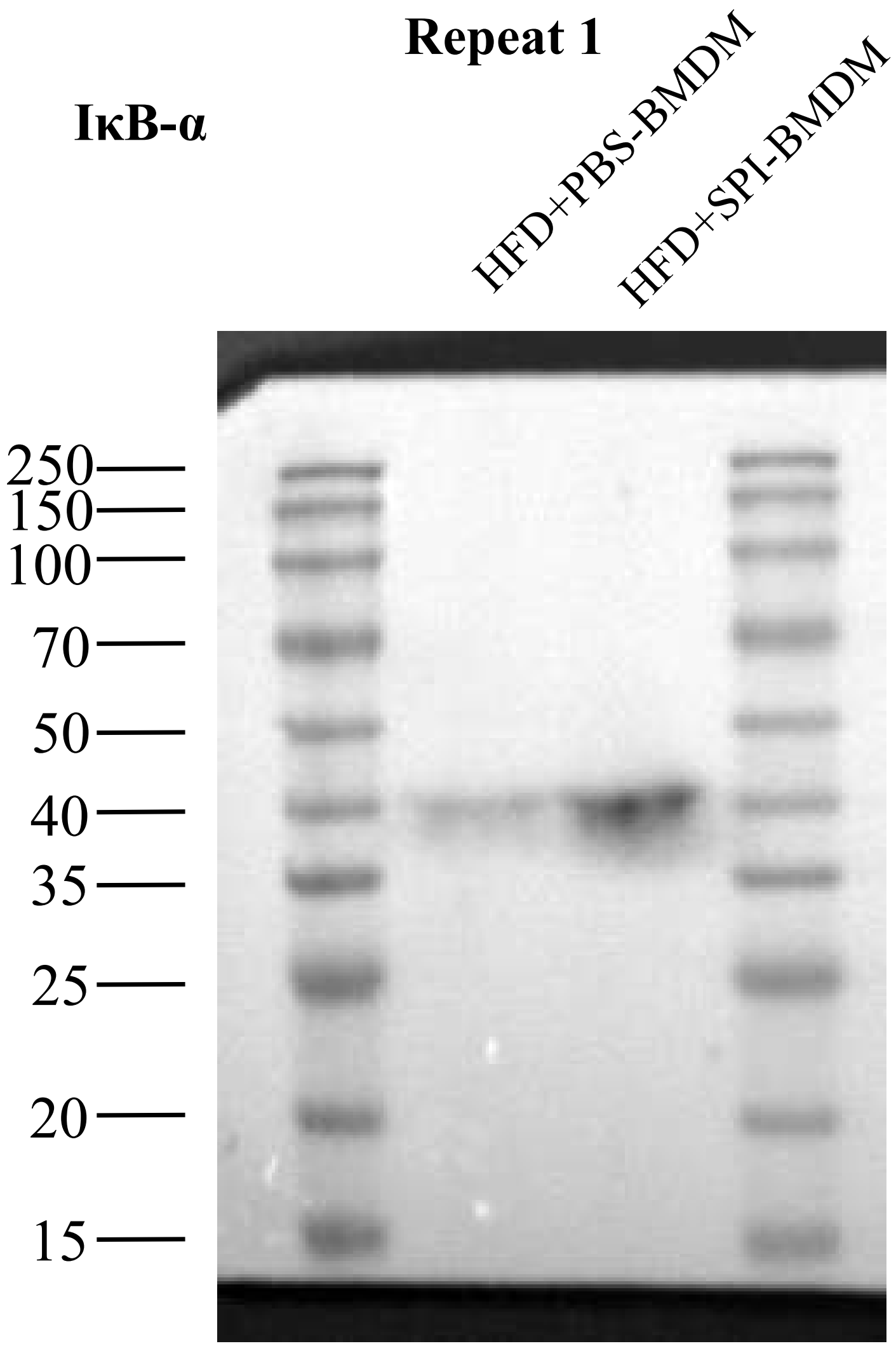
Repeat 1
HFD+PBS-BMDM
HFD+SPI-BMDM
250
150
100
70
50
40
35
25
20
15

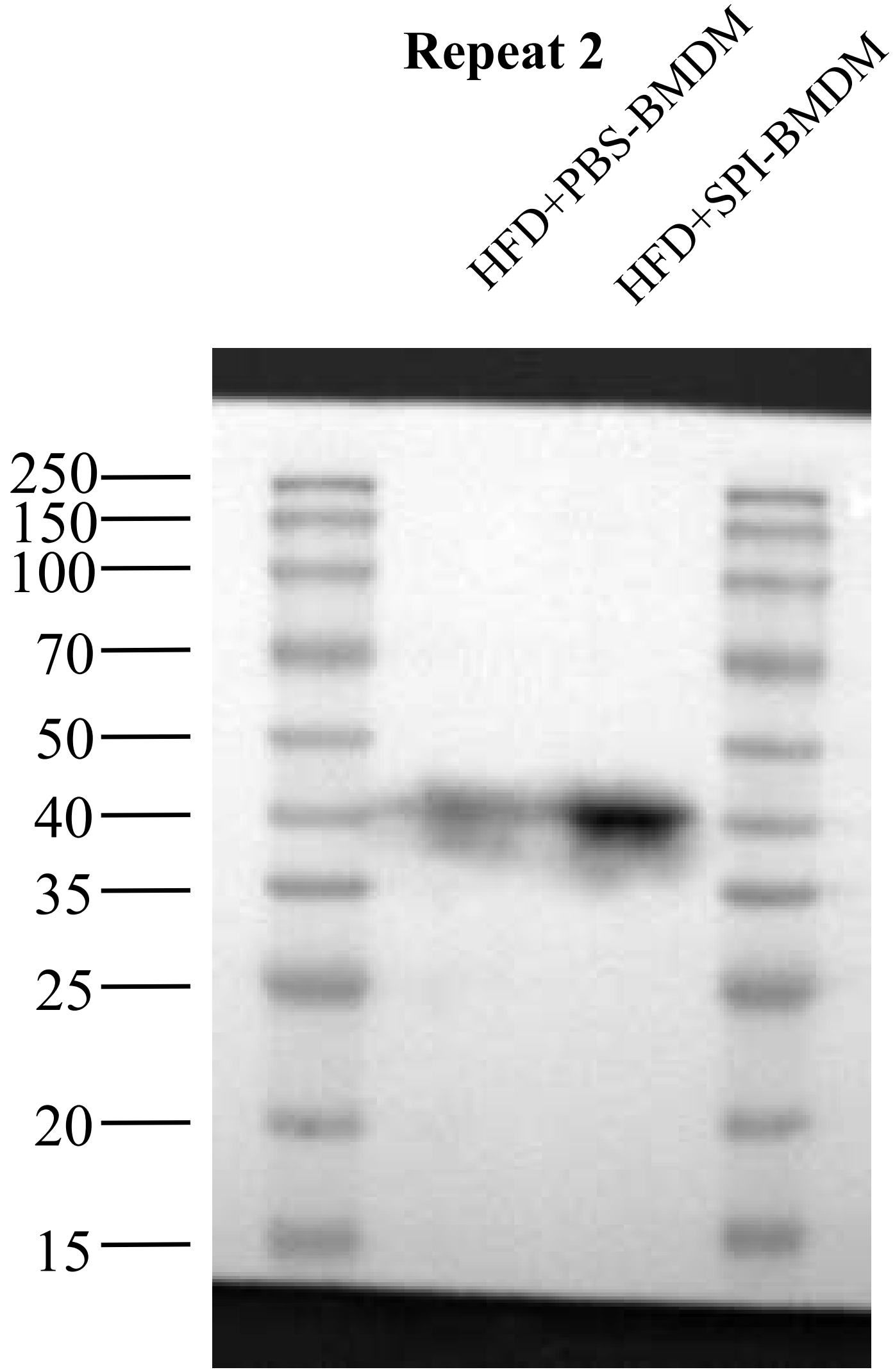
Repeat 2
HFD+PBS-BMDM
HFD+SPI-BMDM
250
150
100
70
50
40
35
25
20
15

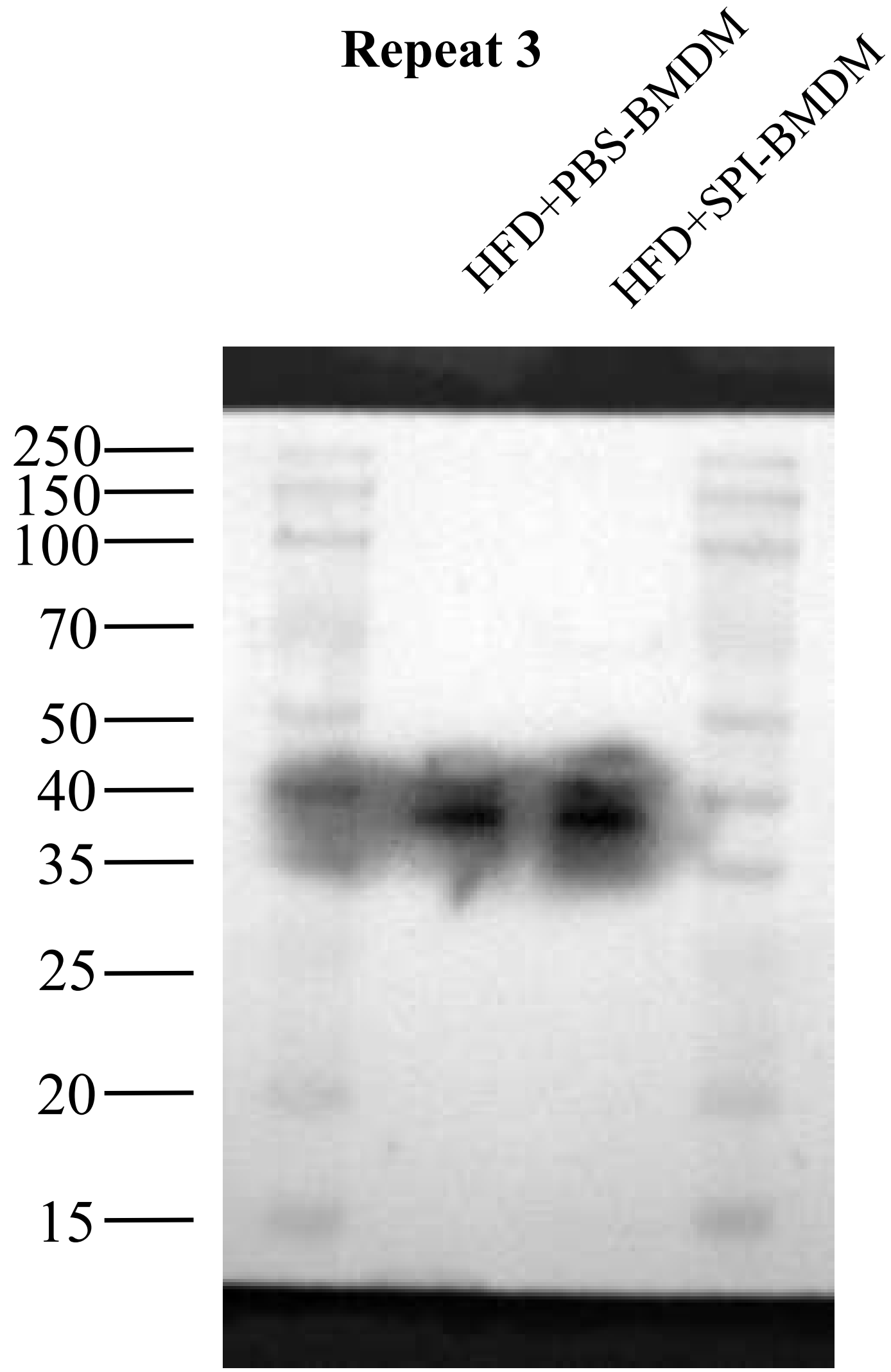
Repeat 3
HFD+PBS-BMDM
HFD+SPI-BMDM
250
150
100
70
50
40
35
25
20
15

Fig.6H

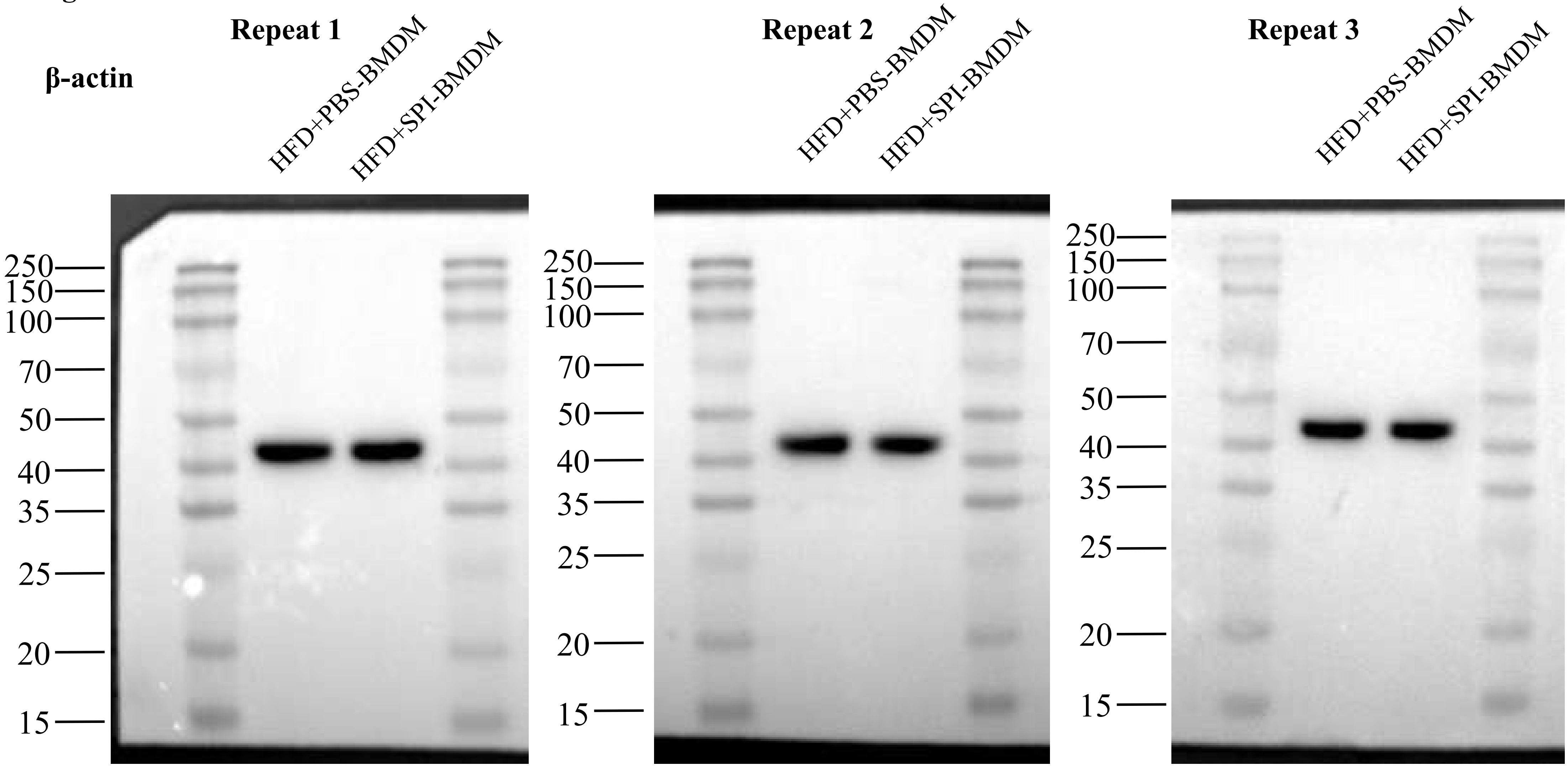

β-actin
Repeat 1
HFD+PBS-BMDM
HFD+SPI-BMDM
Repeat 2
HFD+PBS-BMDM
HFD+SPI-BMDM
Repeat 3
HFD+PBS-BMDM
HFD+SPI-BMDM
250
150
100
70
50
40
35
25
20
15

Fig.6H

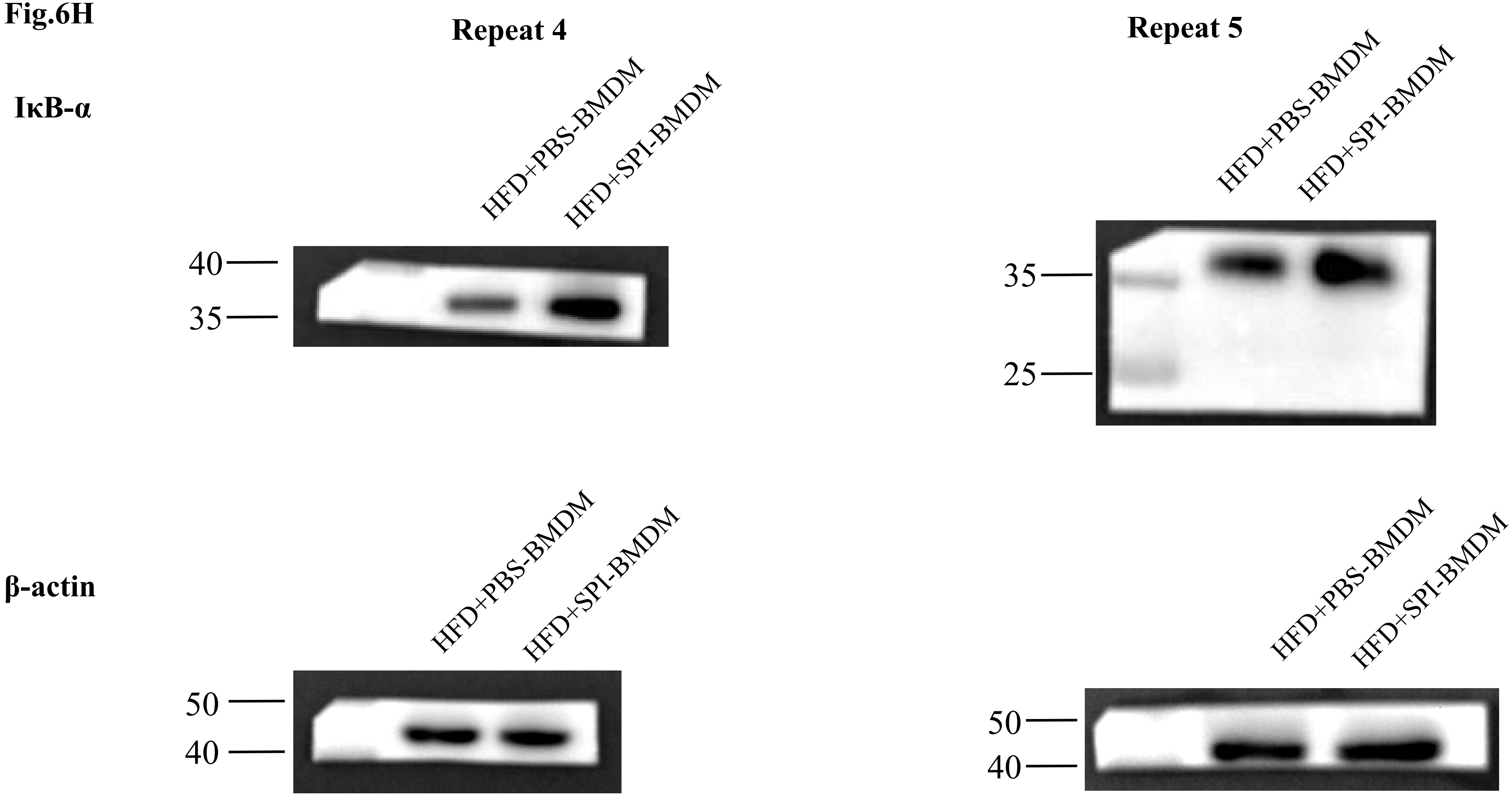

Repeat 4
IκB-α
HFD+PBS-BMDM
HFD+SPI-BMDM
40
35
β-actin
HFD+PBS-BMDM
HFD+SPI-BMDM
50
40
Repeat 5
HFD+PBS-BMDM
HFD+SPI-BMDM
35
25
HFD+PBS-BMDM
HFD+SPI-BMDM
50
40

Fig.7H

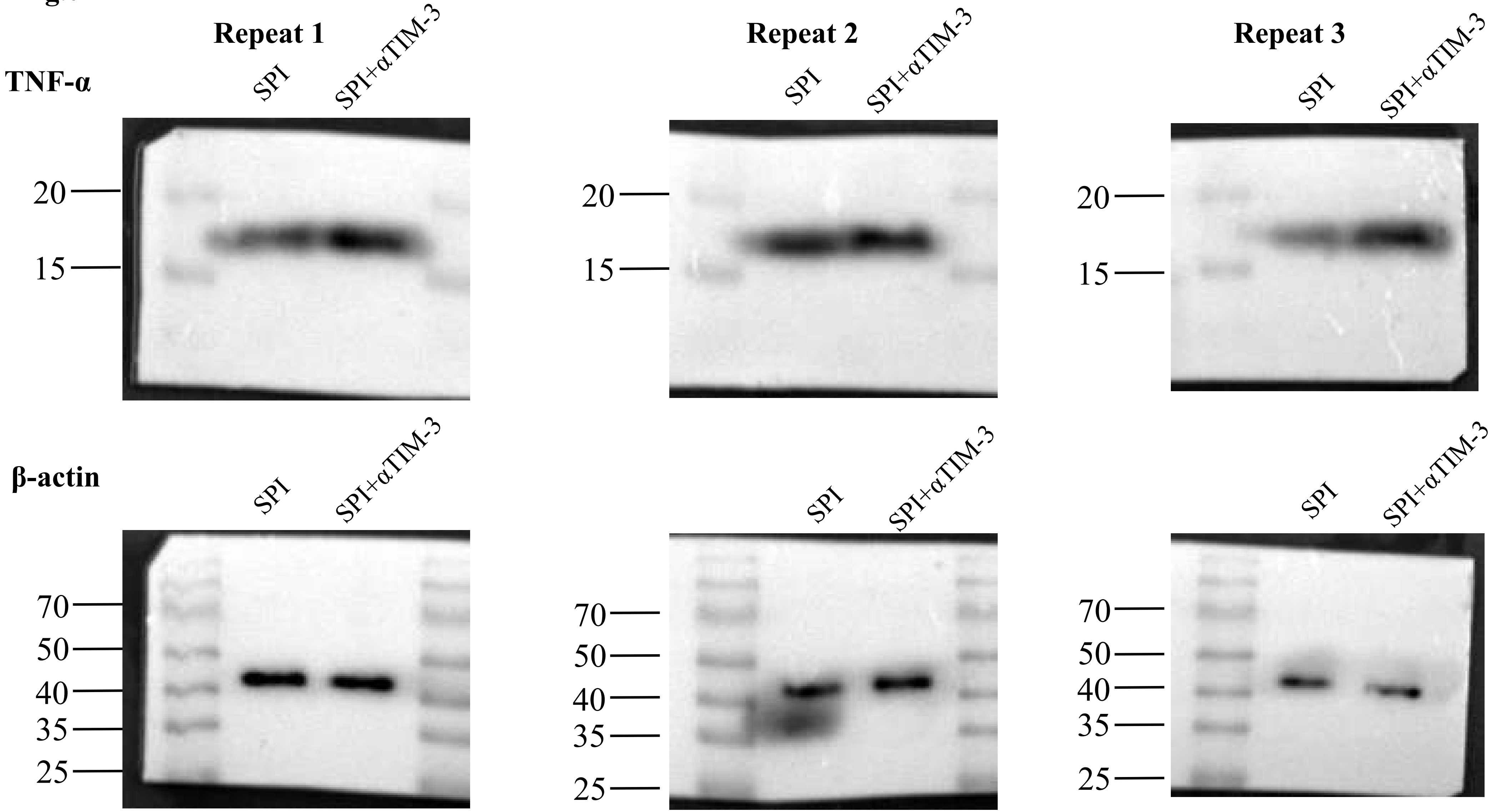

Repeat 1
Repeat 2
Repeat 3
TNF-α
SPI
SPI+αTIM-3
20
15
β-actin
70
50
40
35
25

Fig.7H

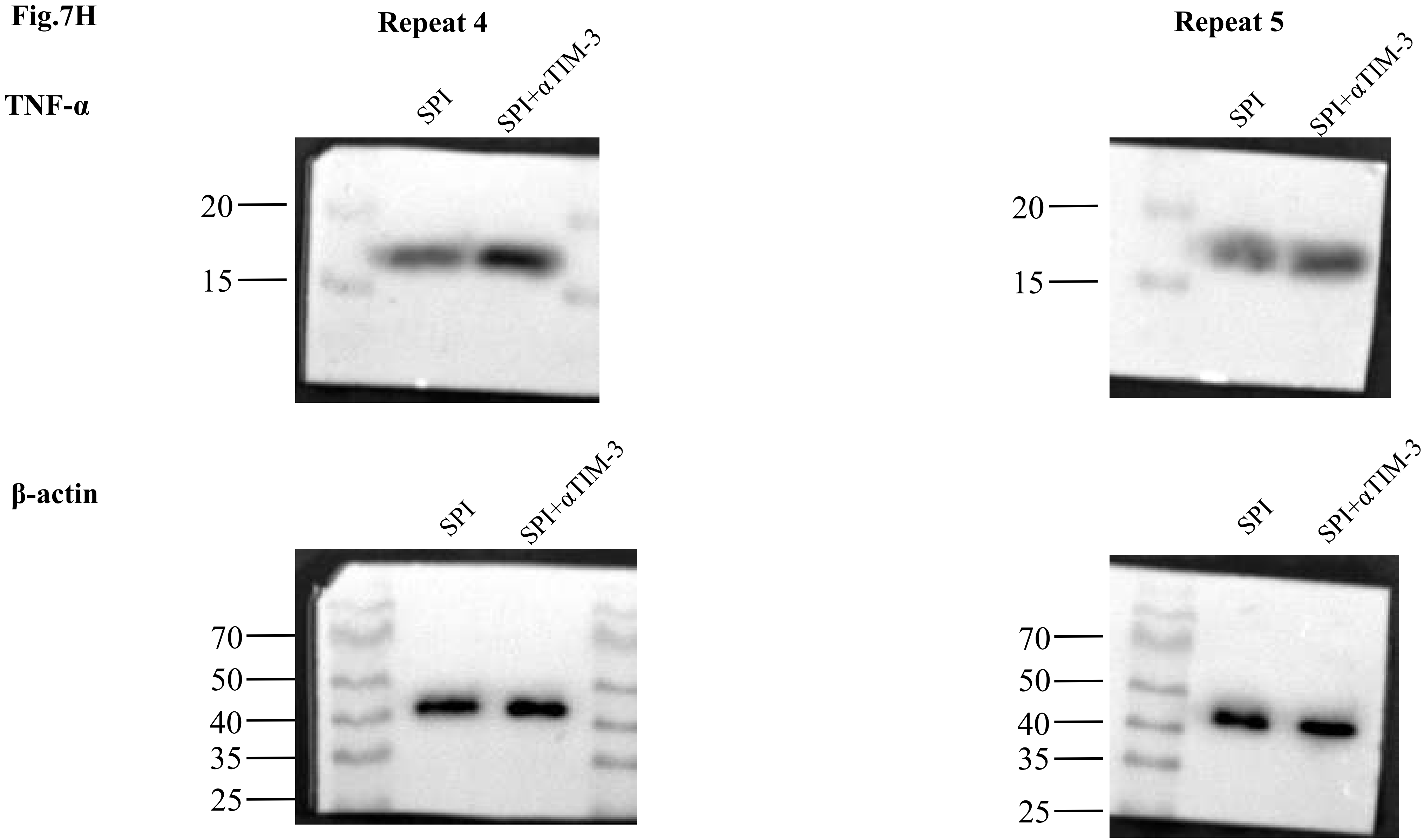

Fig.7H

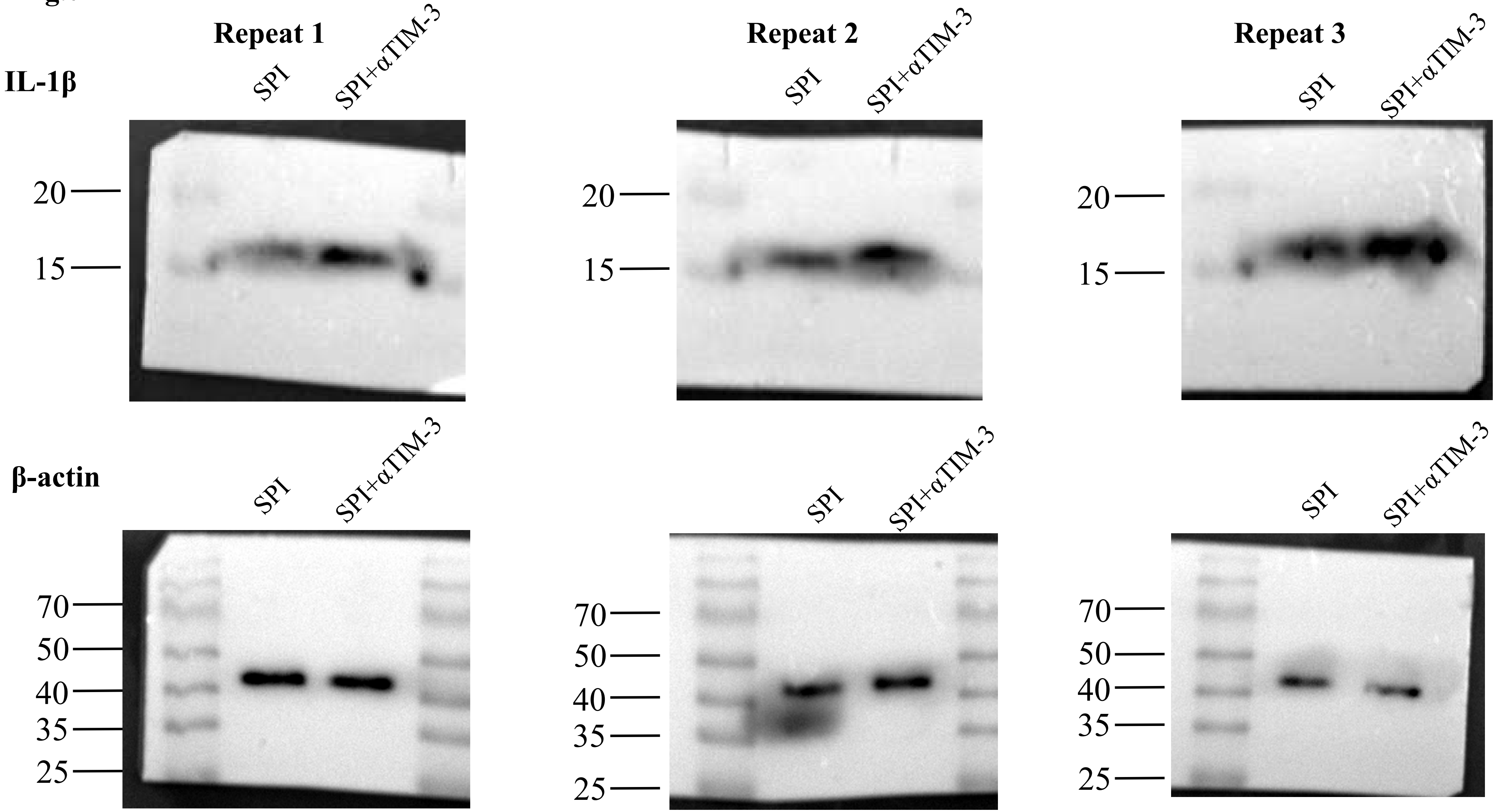
Repeat 1
Repeat 2
Repeat 3
IL-1β
SPI
SPI+αTIM-3
20
15
β-actin
70
50
40
35
25

Fig.7H

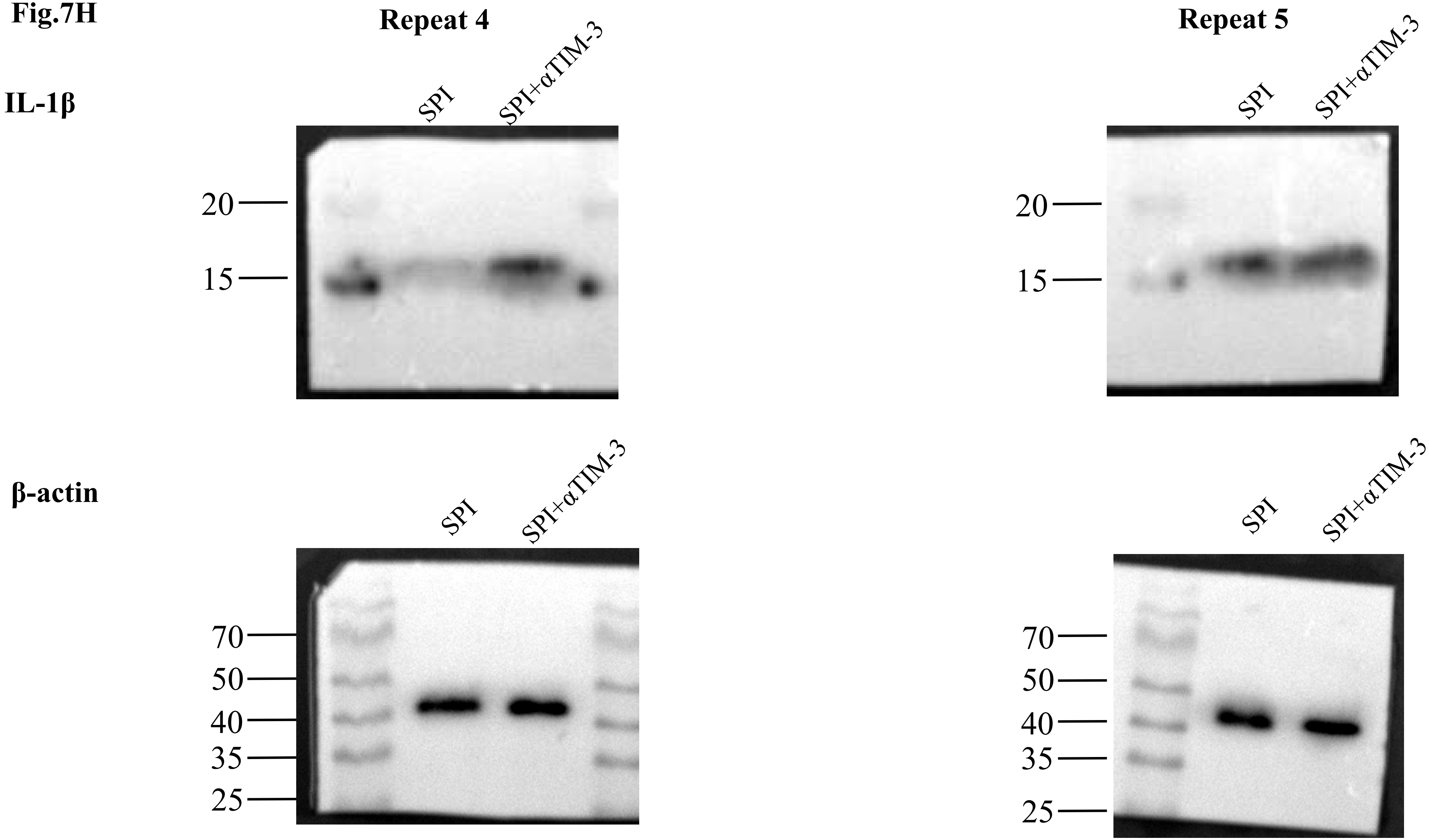

Fig.7H

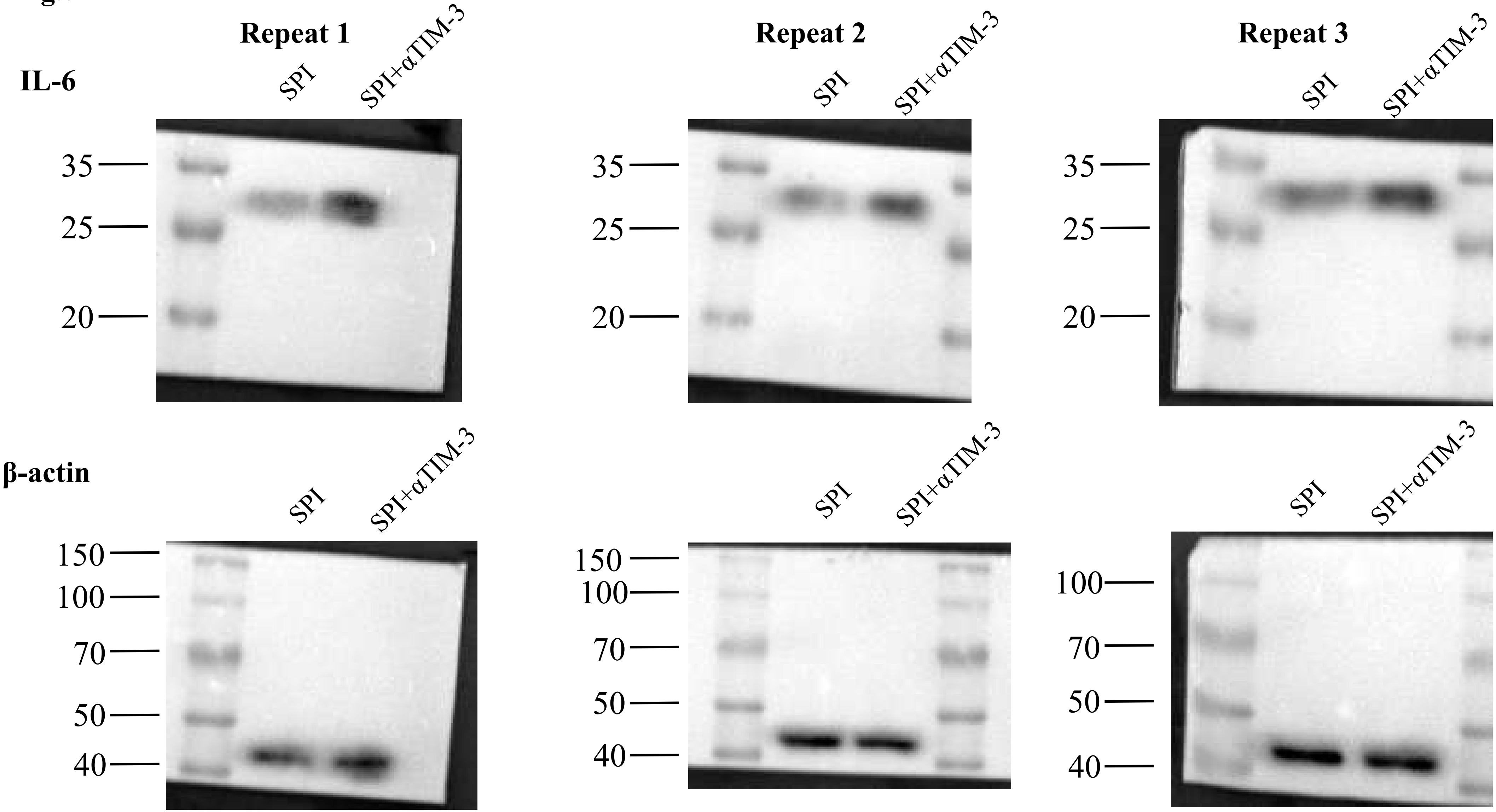
Repeat 1
Repeat 2
Repeat 3
IL-6
SPI
SPI+αTIM-3
35
25
20
β-actin
150
100
70
50
40

Fig.7H

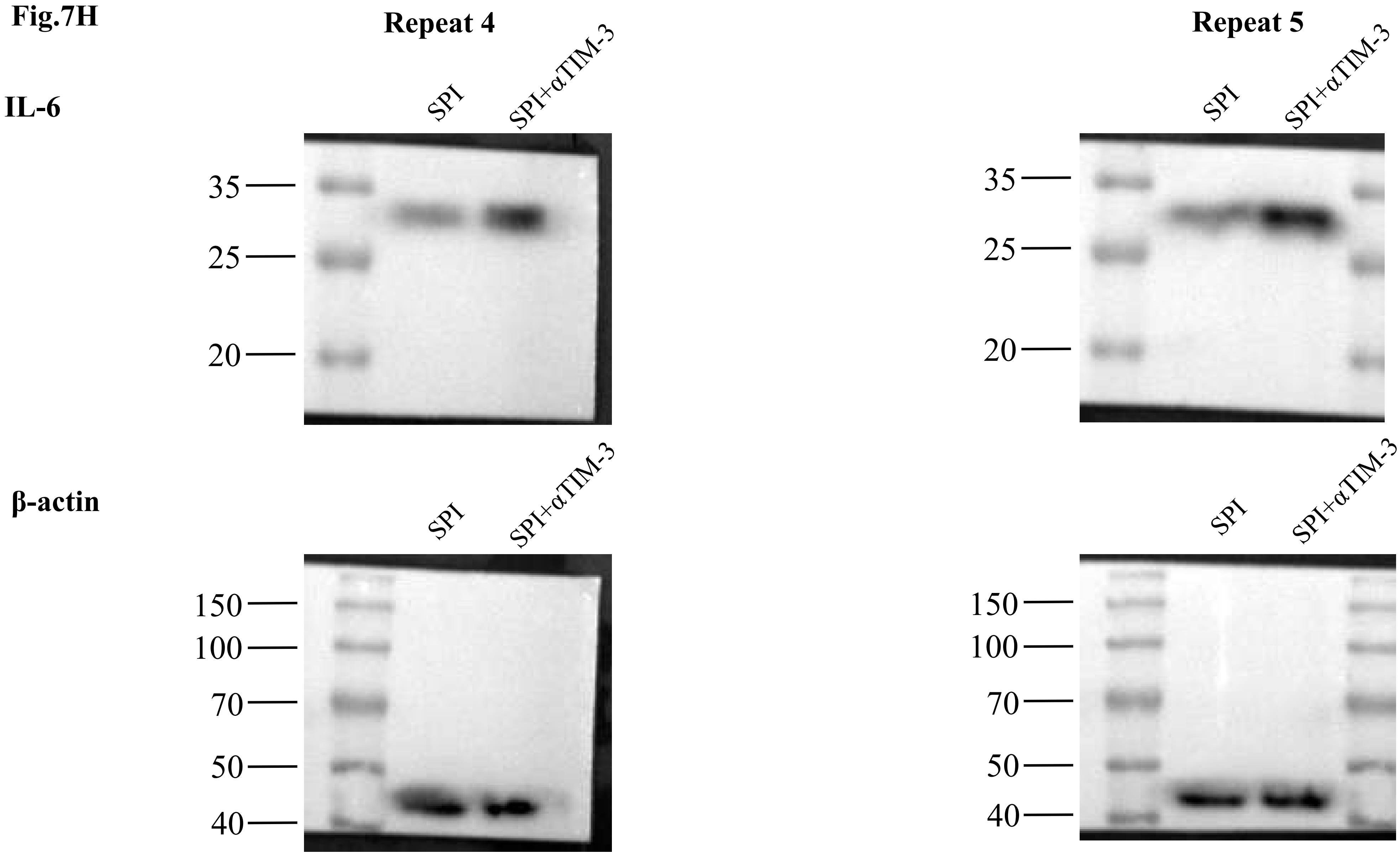
Repeat 4
Repeat 5
IL-6
SPI
SPI+αTIM-3
35
25
20
β-actin
150
100
70
50
40

Fig.7H

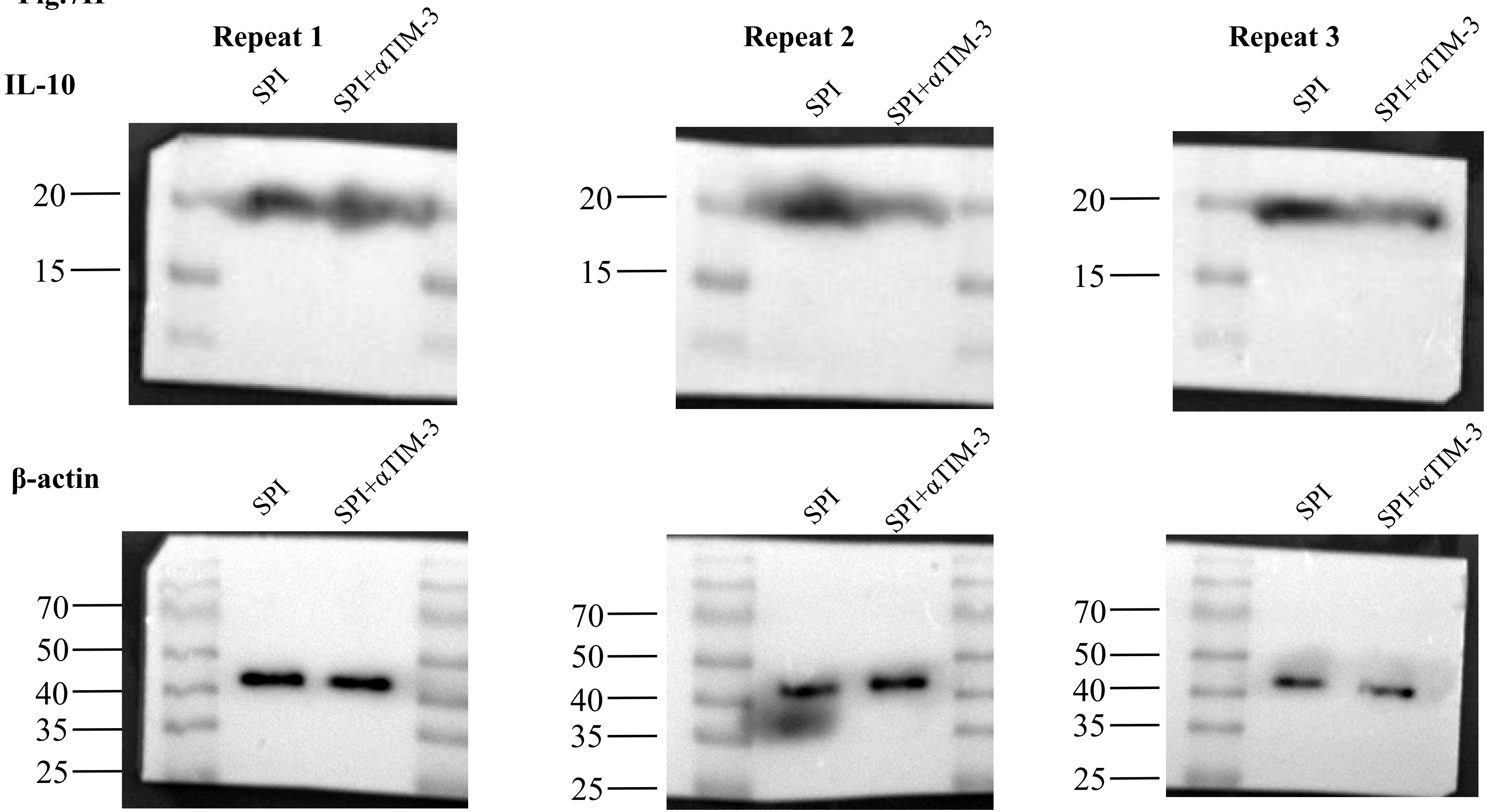
Repeat 1
Repeat 2
Repeat 3
IL-10
SPI
SPI+αTIM-3
20
15
β-actin
70
50
40
35
25

Fig.7H

IL-10

β-actin

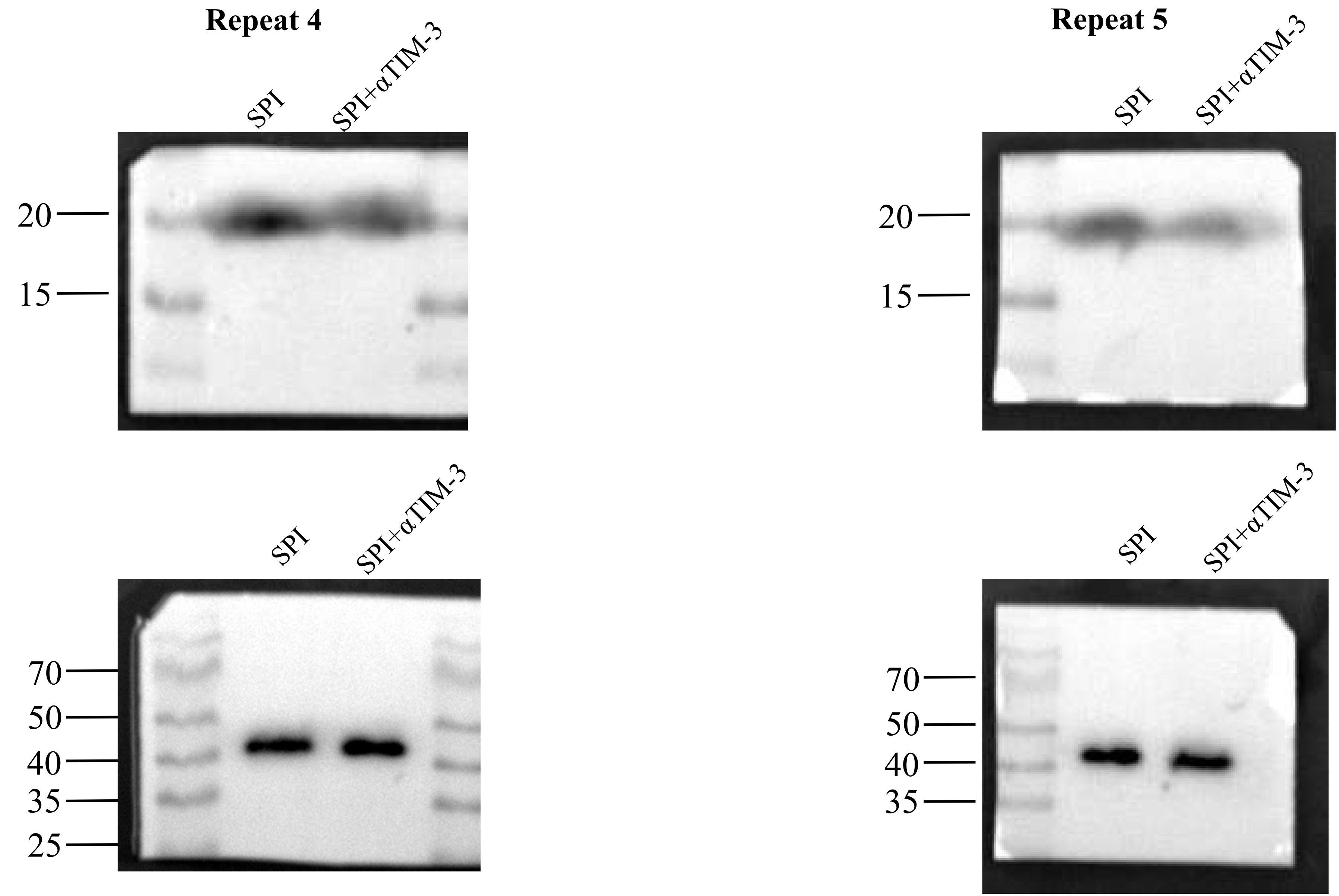

Fig.7I

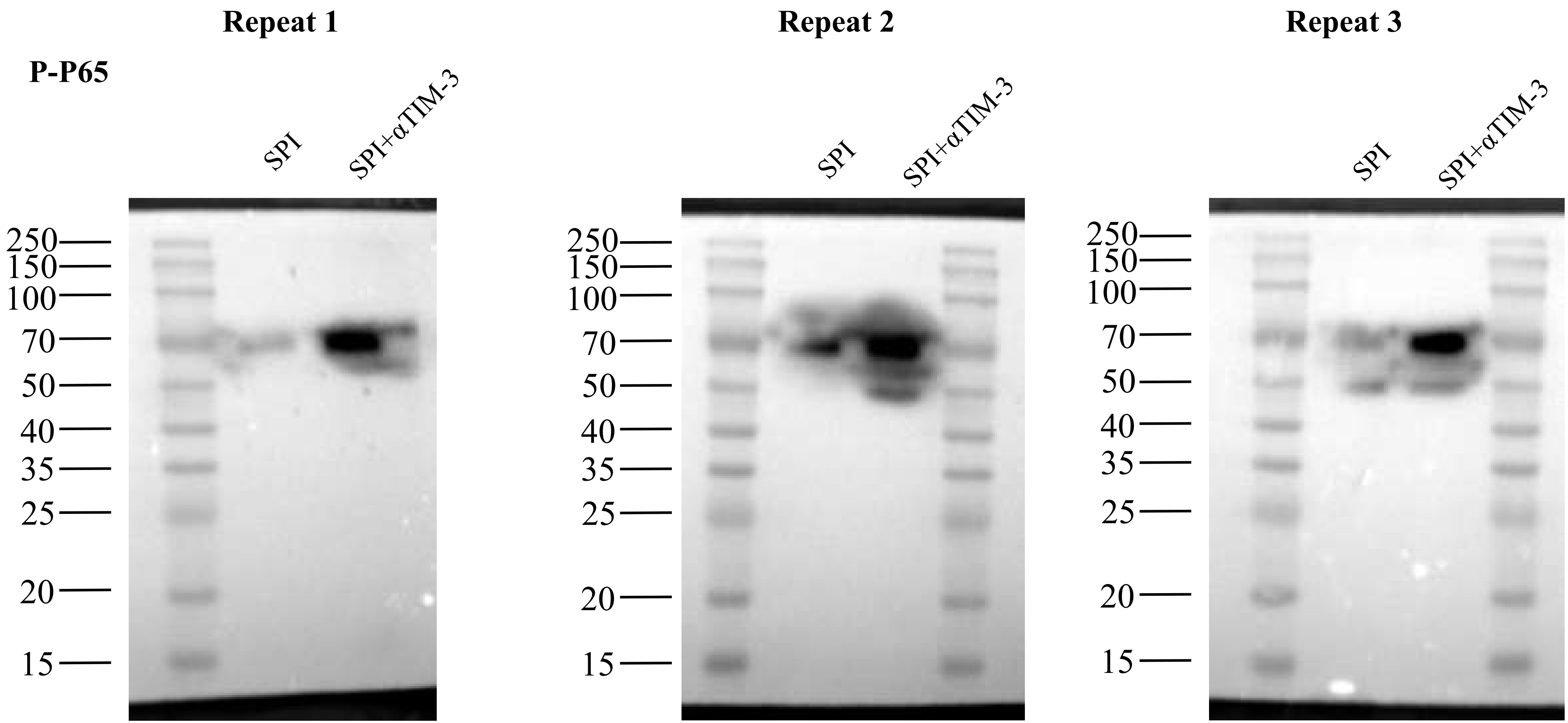
Repeat 1
Repeat 2
Repeat 3
P-P65
SPI
SPI+αTIM-3
250
150
100
70
50
40
35
25
20
15

Fig.7I

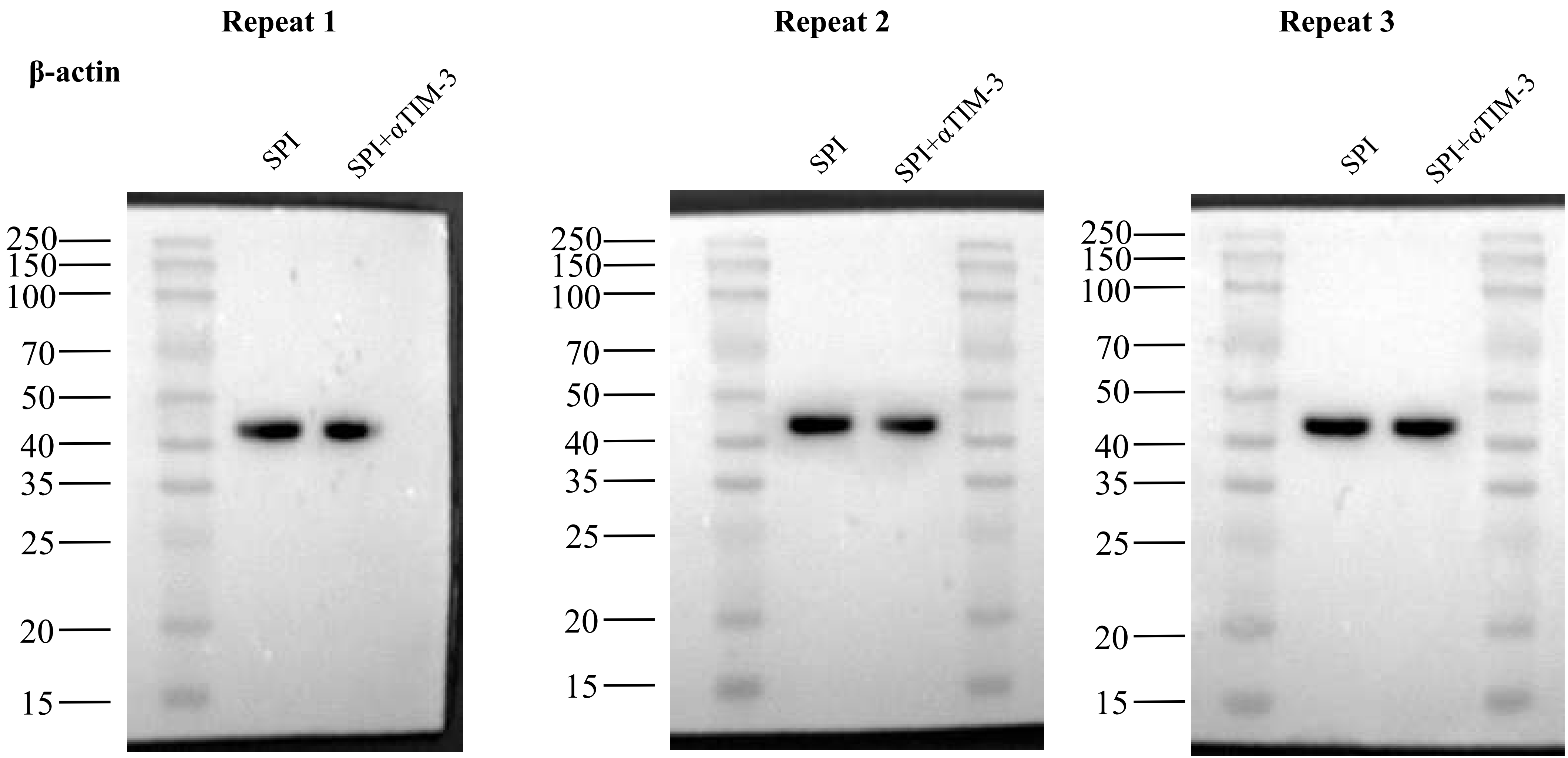
Repeat 1
Repeat 2
Repeat 3
β-actin
SPI
SPI+αTIM-3
250
150
100
70
50
40
35
25
20
15

Fig.7I

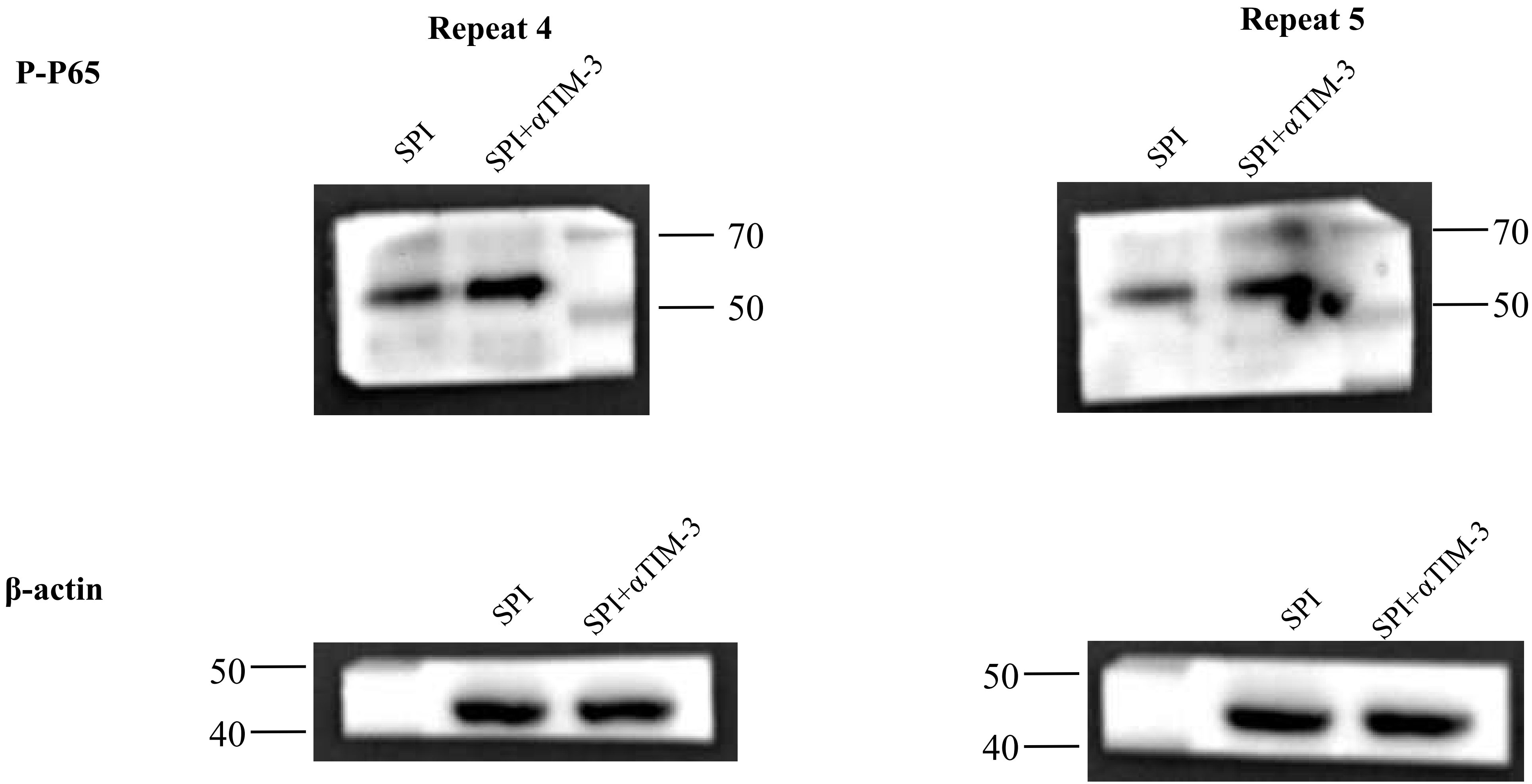

Repeat 4
Repeat 5
P-P65
SPI
SPI+αTIM-3
SPI
SPI+αTIM-3
70
50
70
50
β-actin
SPI
SPI+αTIM-3
SPI
SPI+αTIM-3
50
40
50
40

Fig.7I

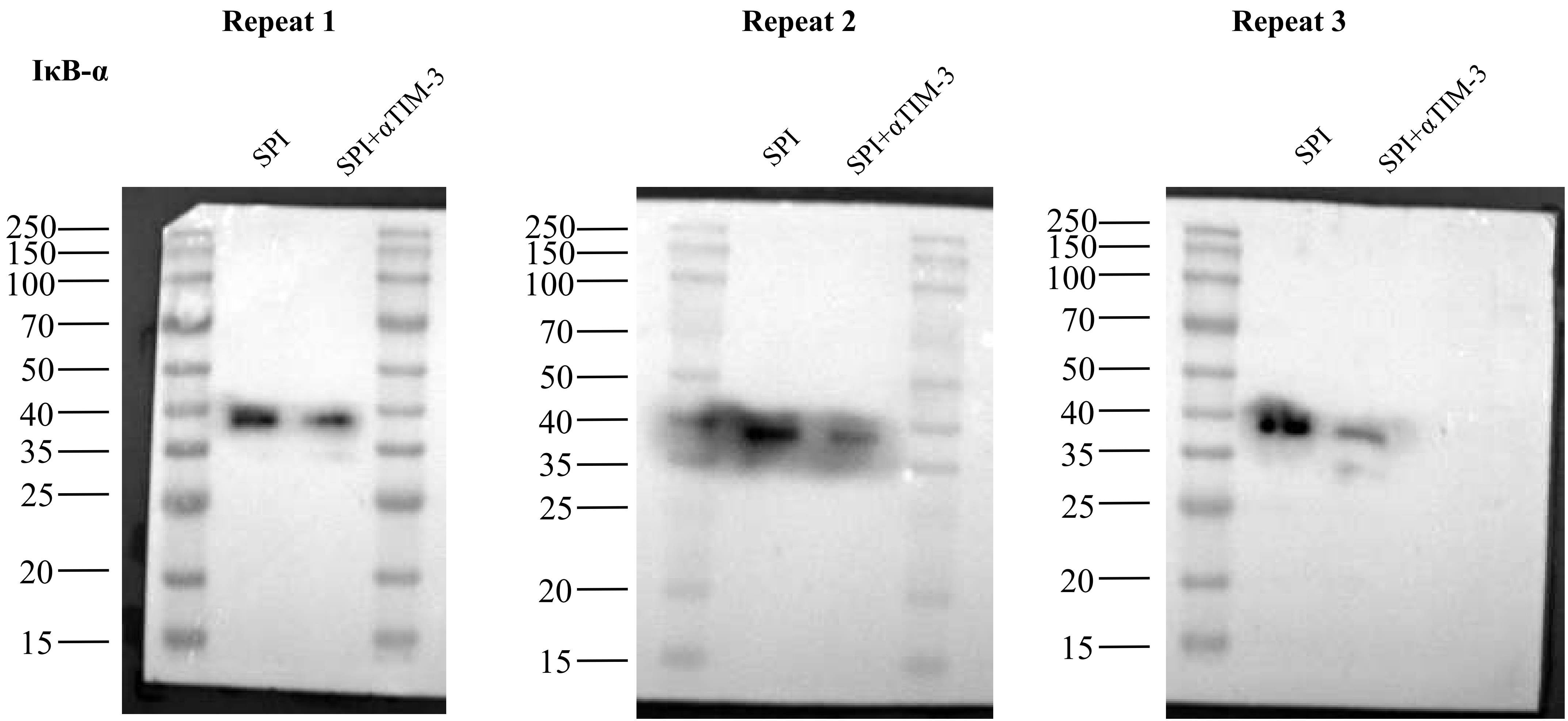
Repeat 1
Repeat 2
Repeat 3
IκB-α
SPI
SPI+αTIM-3
250
150
100
70
50
40
35
25
20
15

Fig.7I

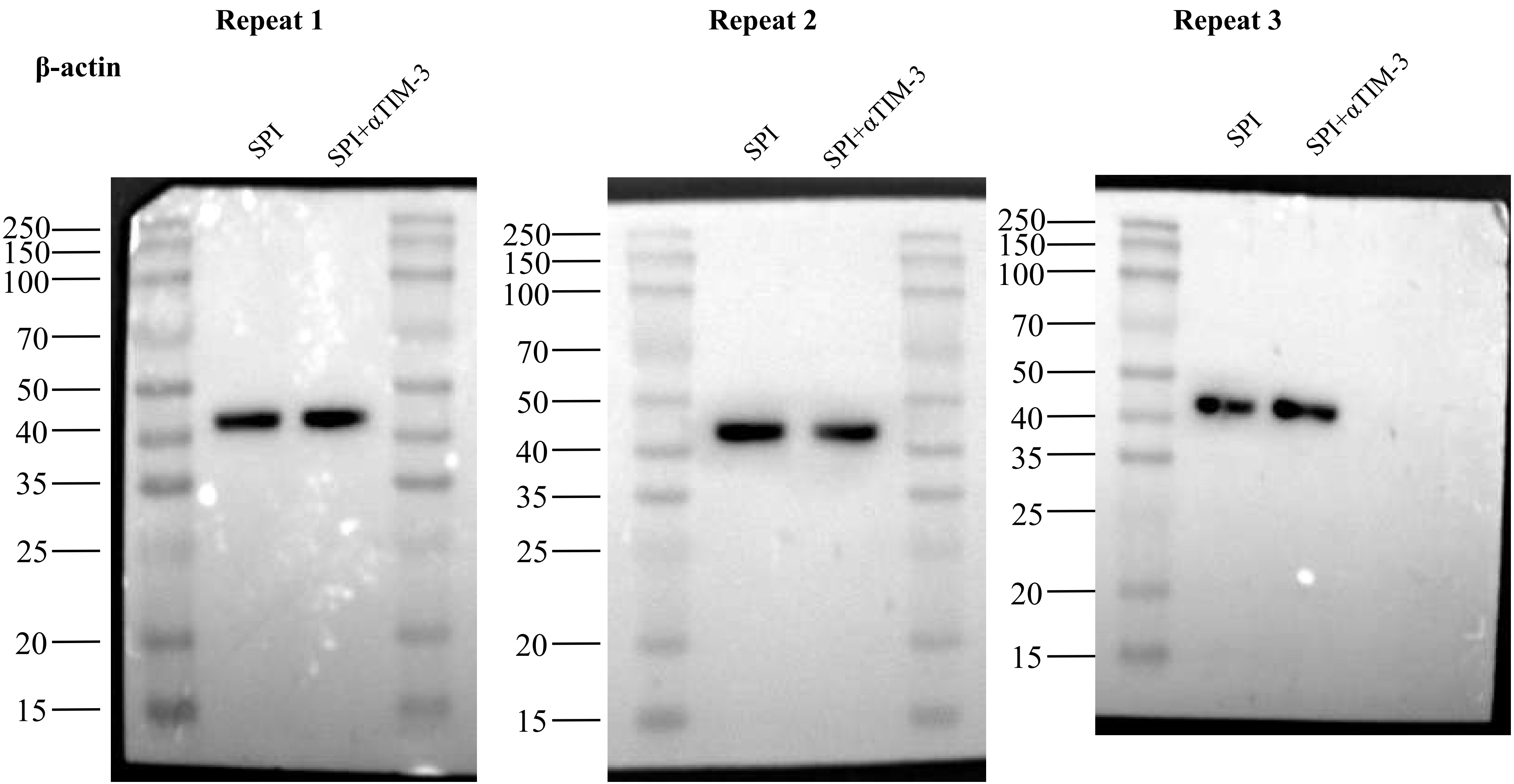
Repeat 1
Repeat 2
Repeat 3
β-actin
SPI
SPI+αTIM-3
250
150
100
70
50
40
35
25
20
15

Fig.7I

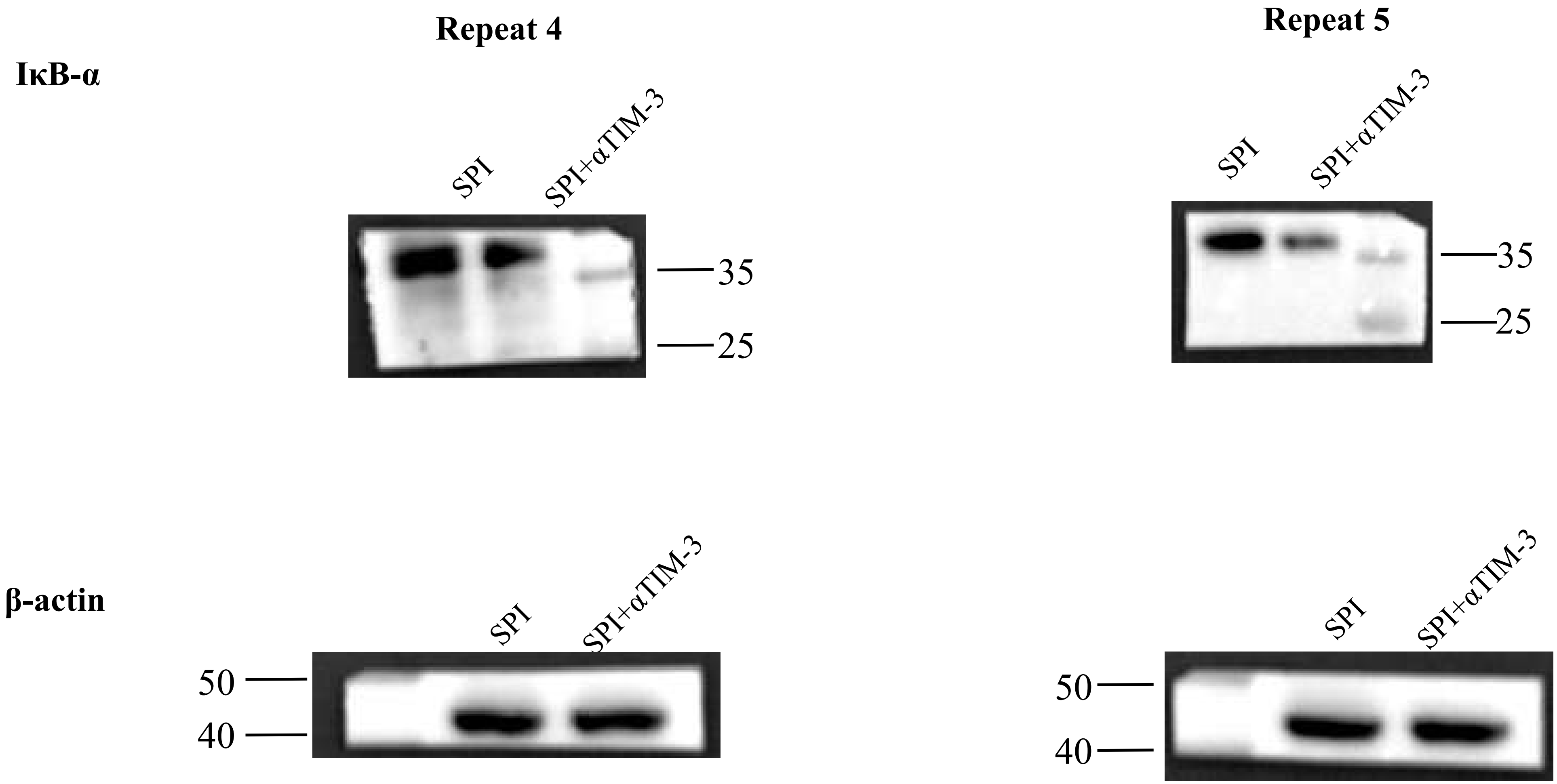

Fig.8A

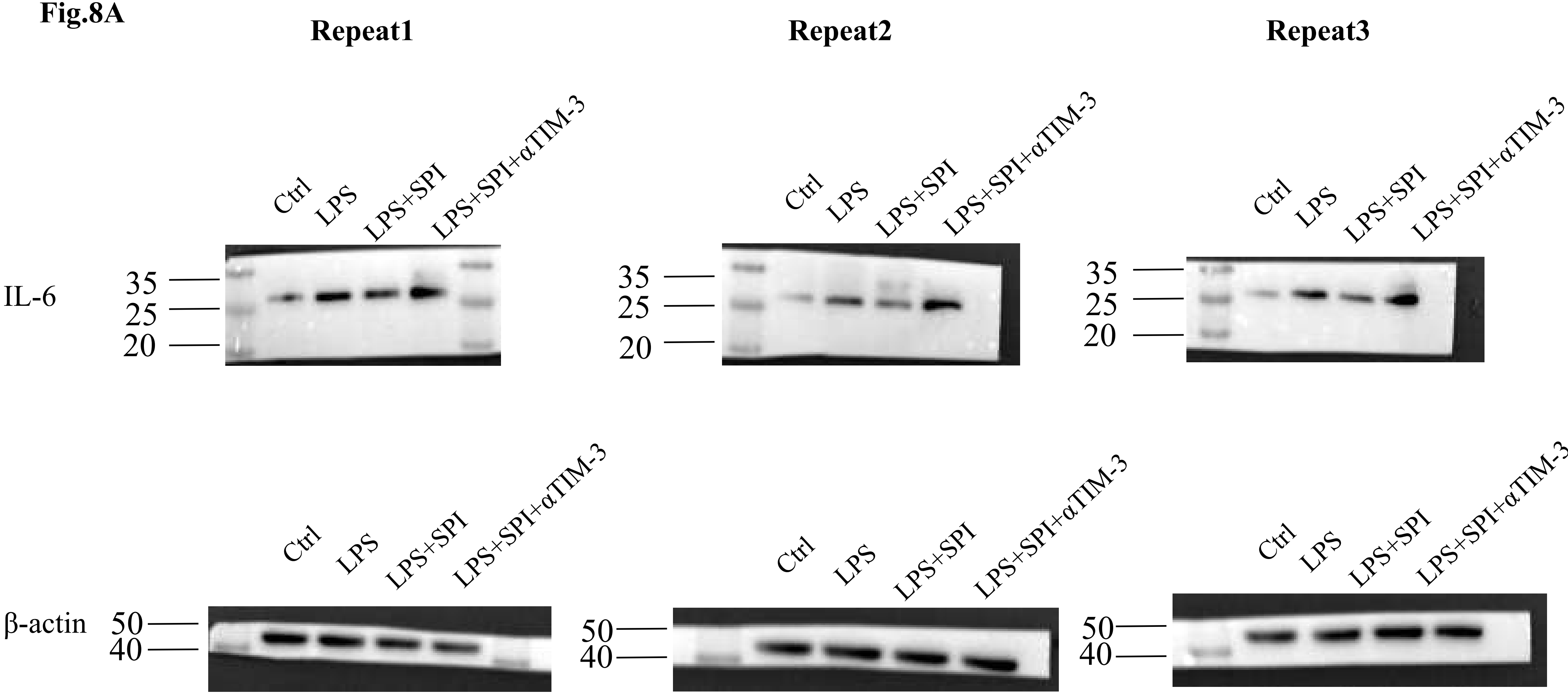
Repeat1
Repeat2
Repeat3
Ctrl
LPS
LPS+SPI
LPS+SPI+αTIM-3
IL-6
35
25
20
β-actin
50
40

Fig.8A

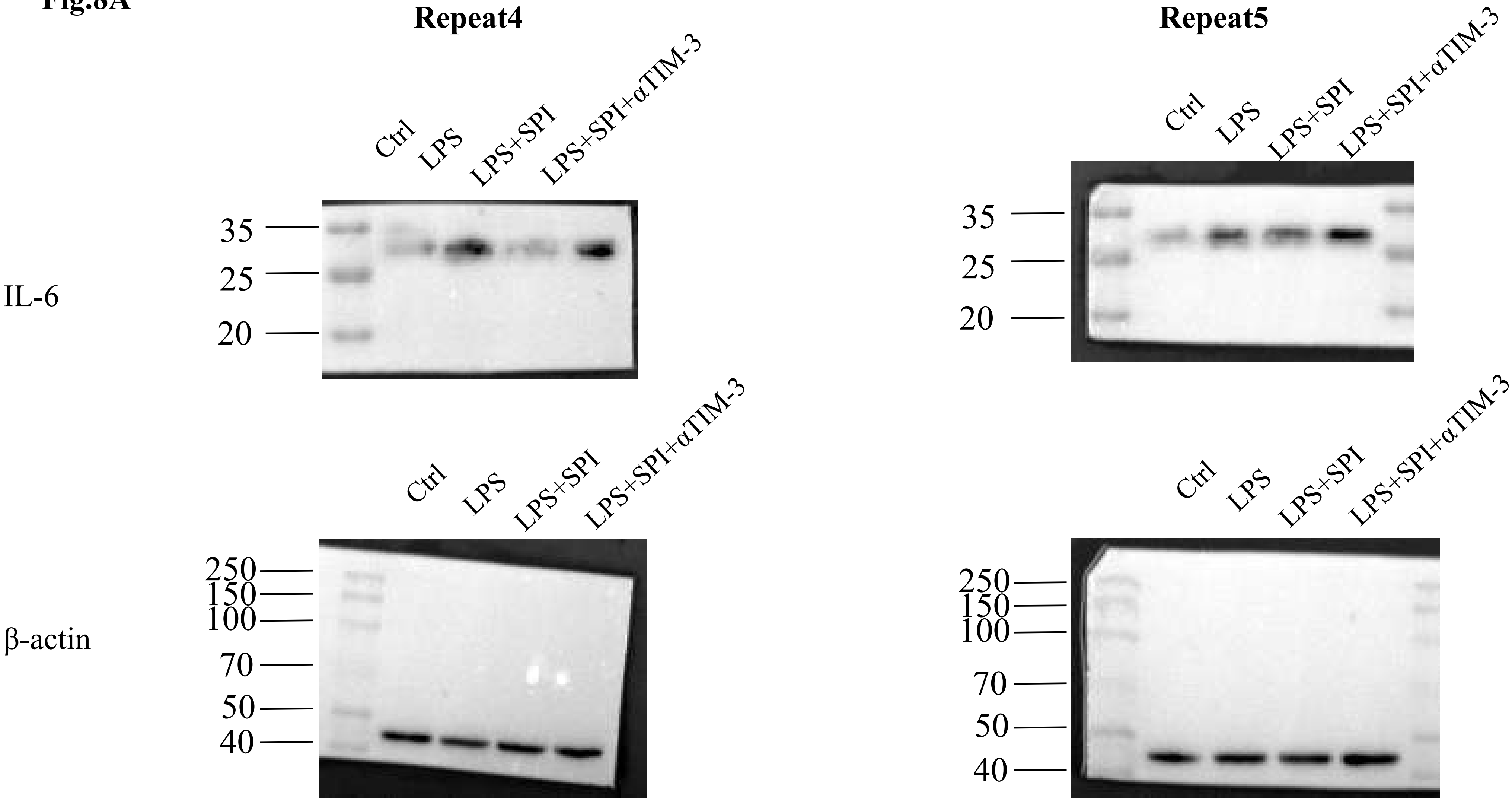

Repeat4
Repeat5
Ctrl
LPS
LPS+SPI
LPS+SPI+αTIM-3
IL-6
35
25
20
β-actin
250
150
100
70
50
40

Fig.8A

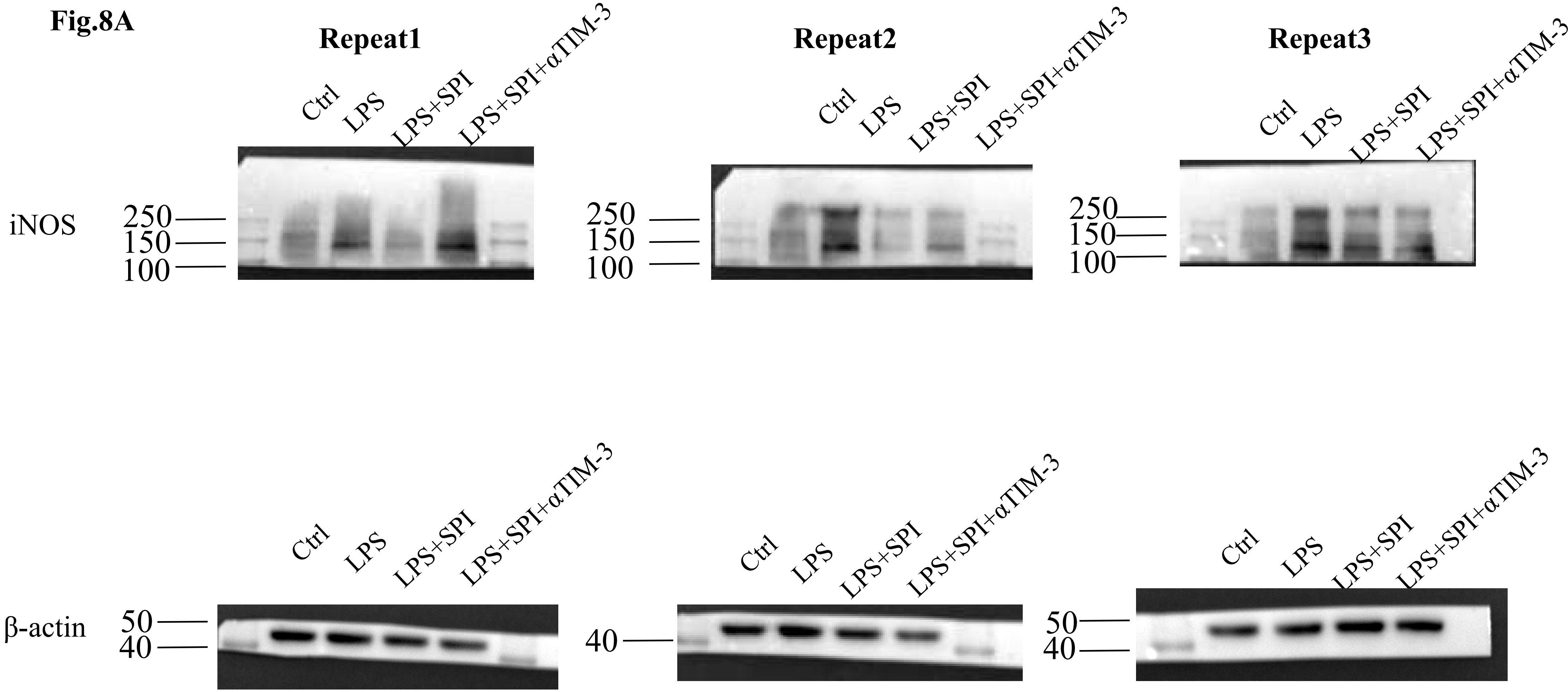

Repeat1
Repeat2
Repeat3
Ctrl
LPS
LPS+SPI
LPS+SPI+αTIM-3
iNOS
250
150
100
β-actin
50
40

Fig.8A

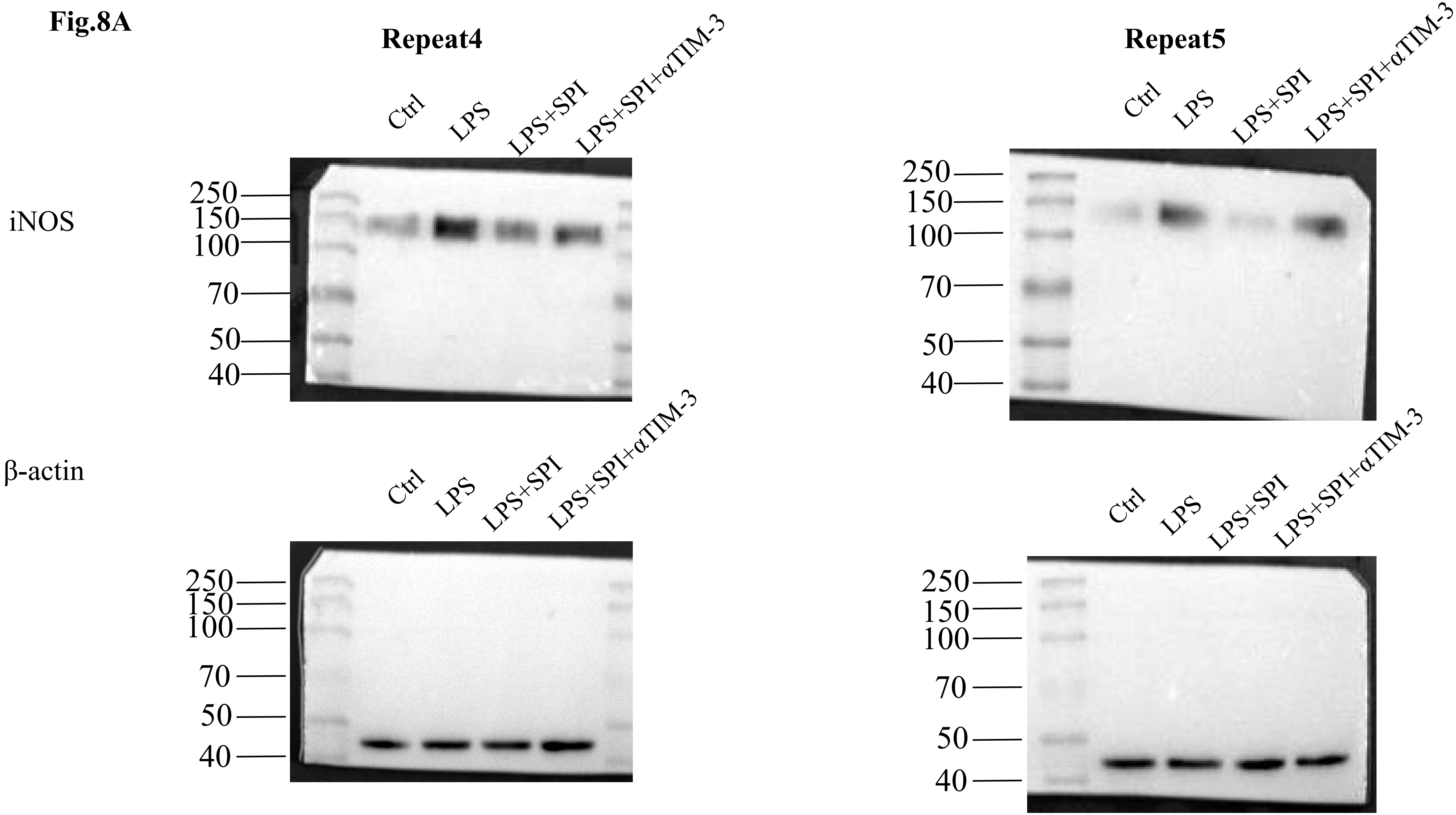
Repeat4
Repeat5
iNOS
β-actin
Ctrl
LPS
LPS+SPI
LPS+SPI+αTIM-3
250
150
100
70
50
40

Fig.8B

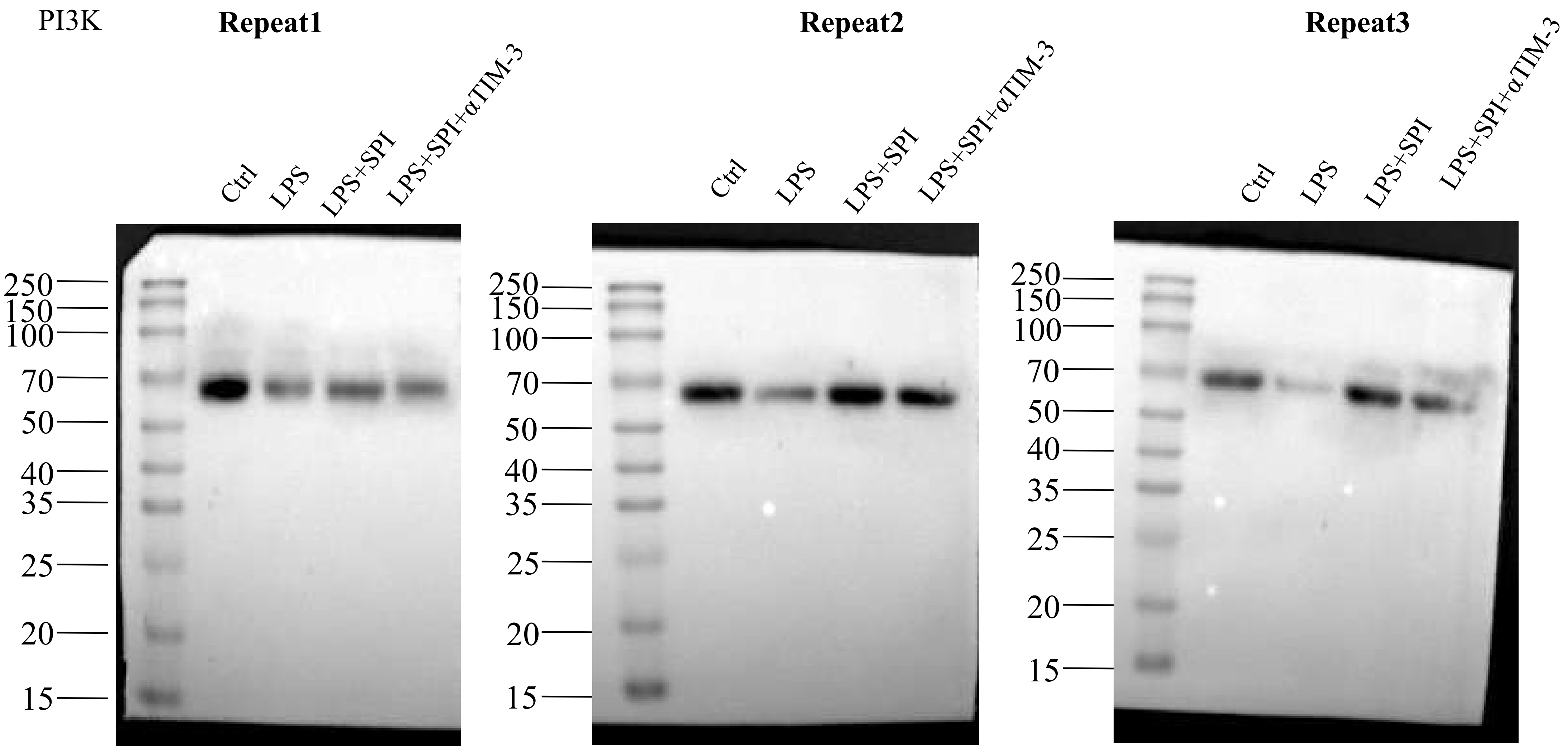

PI3K
Repeat1
Repeat2
Repeat3
Ctrl
LPS
LPS+SPI
LPS+SPI+αTIM-3
250
150
100
70
50
40
35
25
20
15

Fig.8B

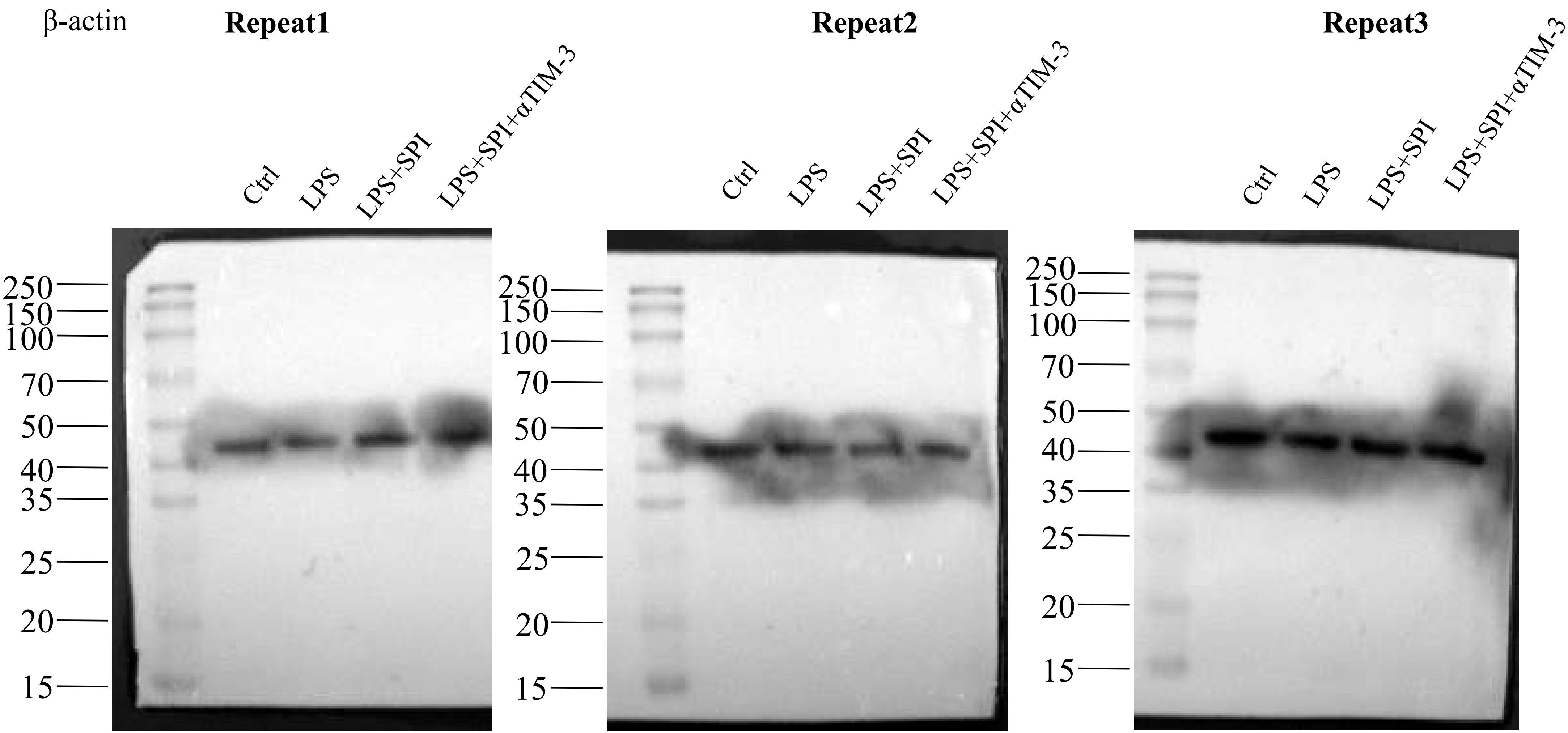
β-actin
Repeat1
Ctrl
LPS
LPS+SPI
LPS+SPI+αTIM-3
250
150
100
70
50
40
35
25
20
15
Repeat2
Ctrl
LPS
LPS+SPI
LPS+SPI+αTIM-3
250
150
100
70
50
40
35
25
20
15
Repeat3
Ctrl
LPS
LPS+SPI
LPS+SPI+αTIM-3
250
150
100
70
50
40
35
25
20
15

Fig.8A

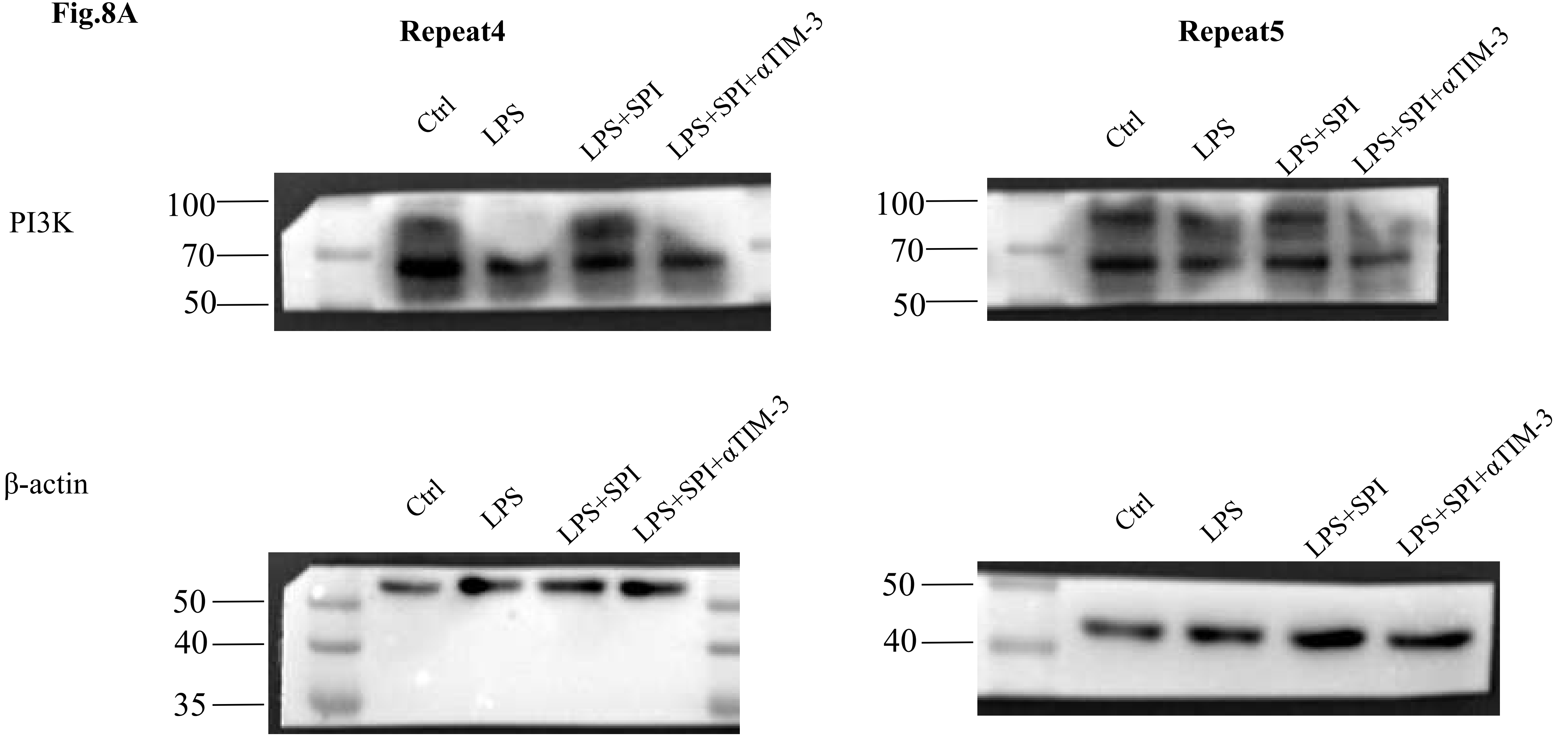
Repeat4
Repeat5
PI3K
Ctrl
LPS
LPS+SPI
LPS+SPI+αTIM-3
100
70
50
β-actin
40
35

Fig.8B

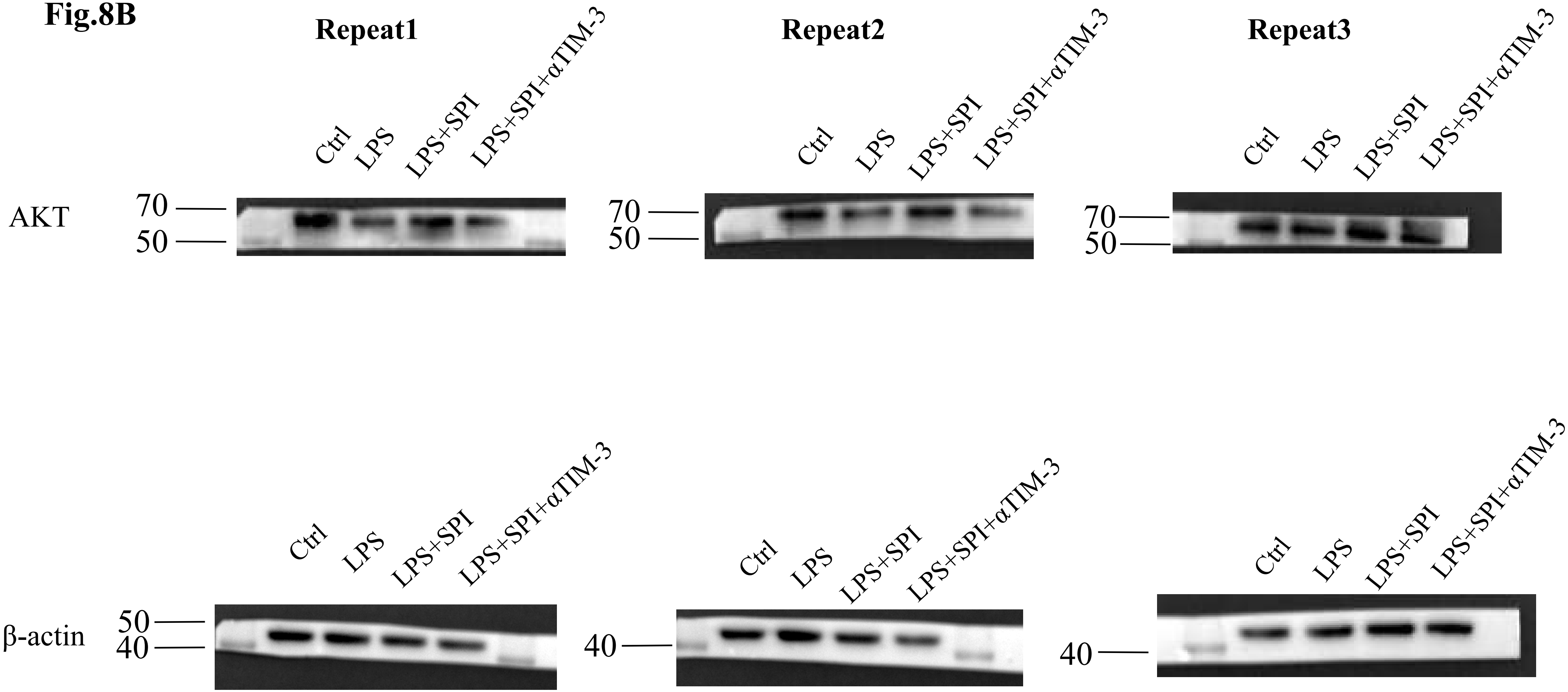

Repeat1
Repeat2
Repeat3
Ctrl
LPS
LPS+SPI
LPS+SPI+αTIM-3
AKT
70
50
β-actin
50
40

Fig.8B

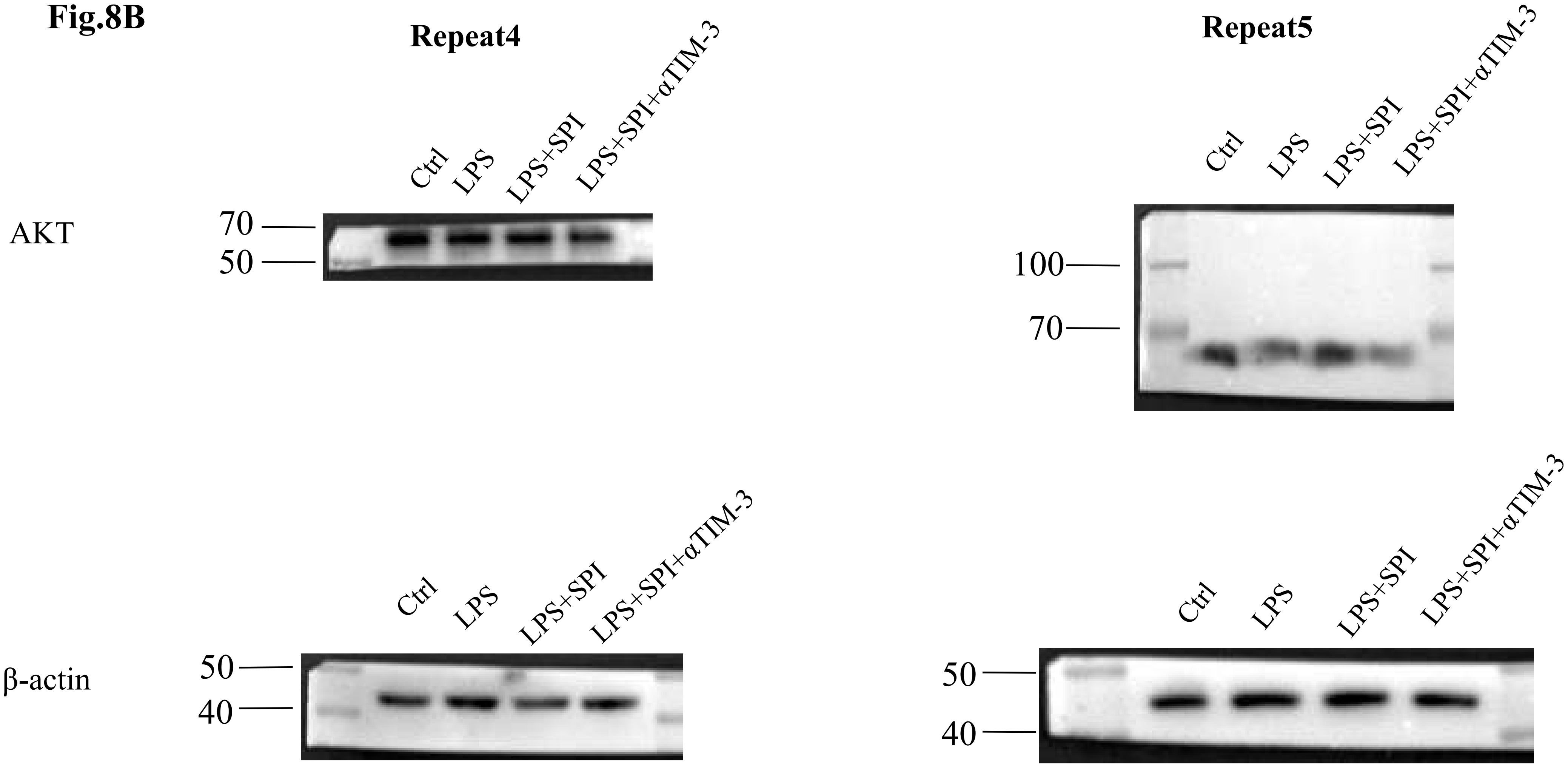

Repeat4
Repeat5
Ctrl
LPS
LPS+SPI
LPS+SPI+αTIM-3
AKT
70
50
100
70
Ctrl
LPS
LPS+SPI
LPS+SPI+αTIM-3
β-actin
50
40
50
40

Fig.8B

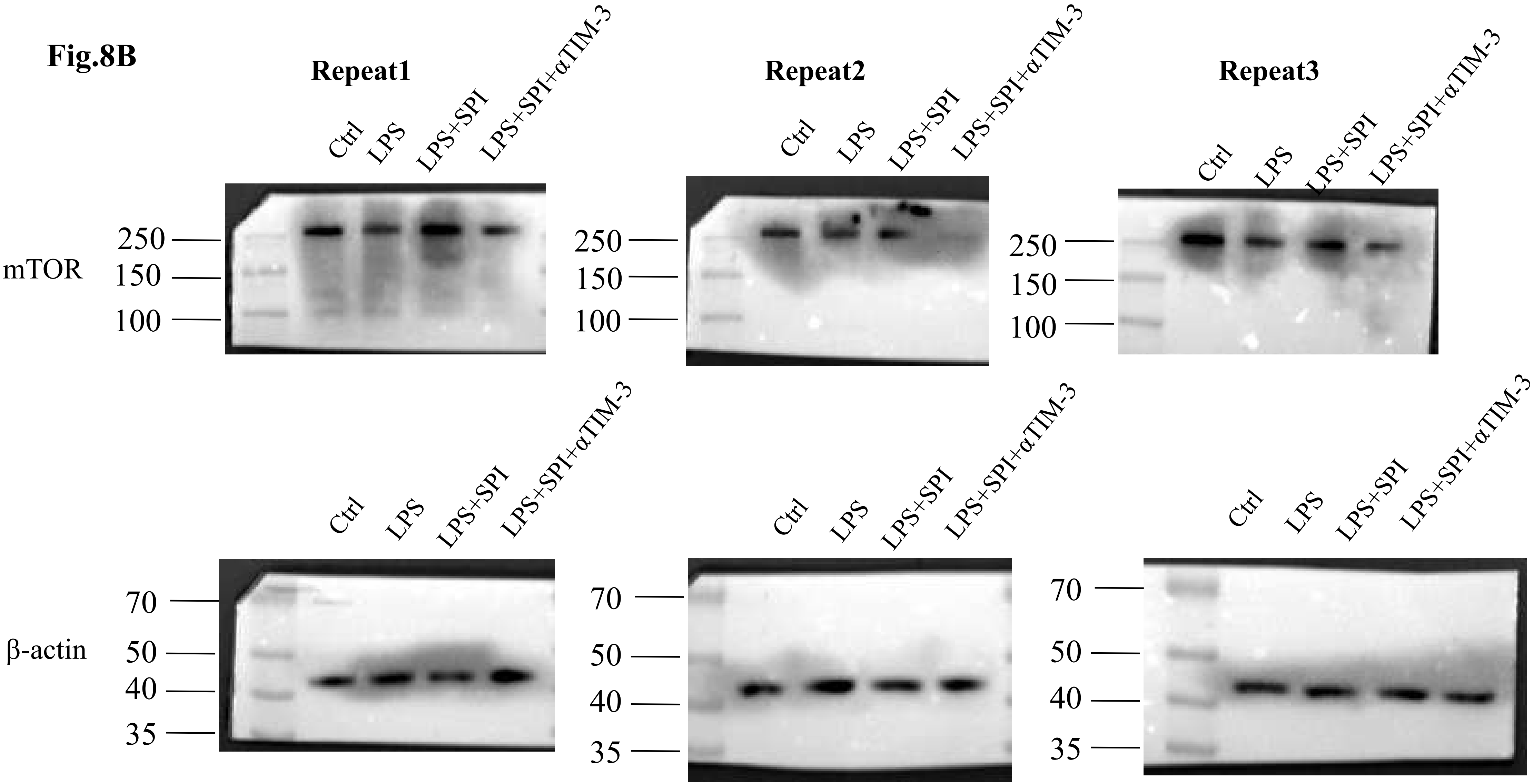

Repeat1
Repeat2
Repeat3
Ctrl
LPS
LPS+SPI
LPS+SPI+αTIM-3
mTOR
250
150
100
β-actin
70
50
40
35

Fig.8B

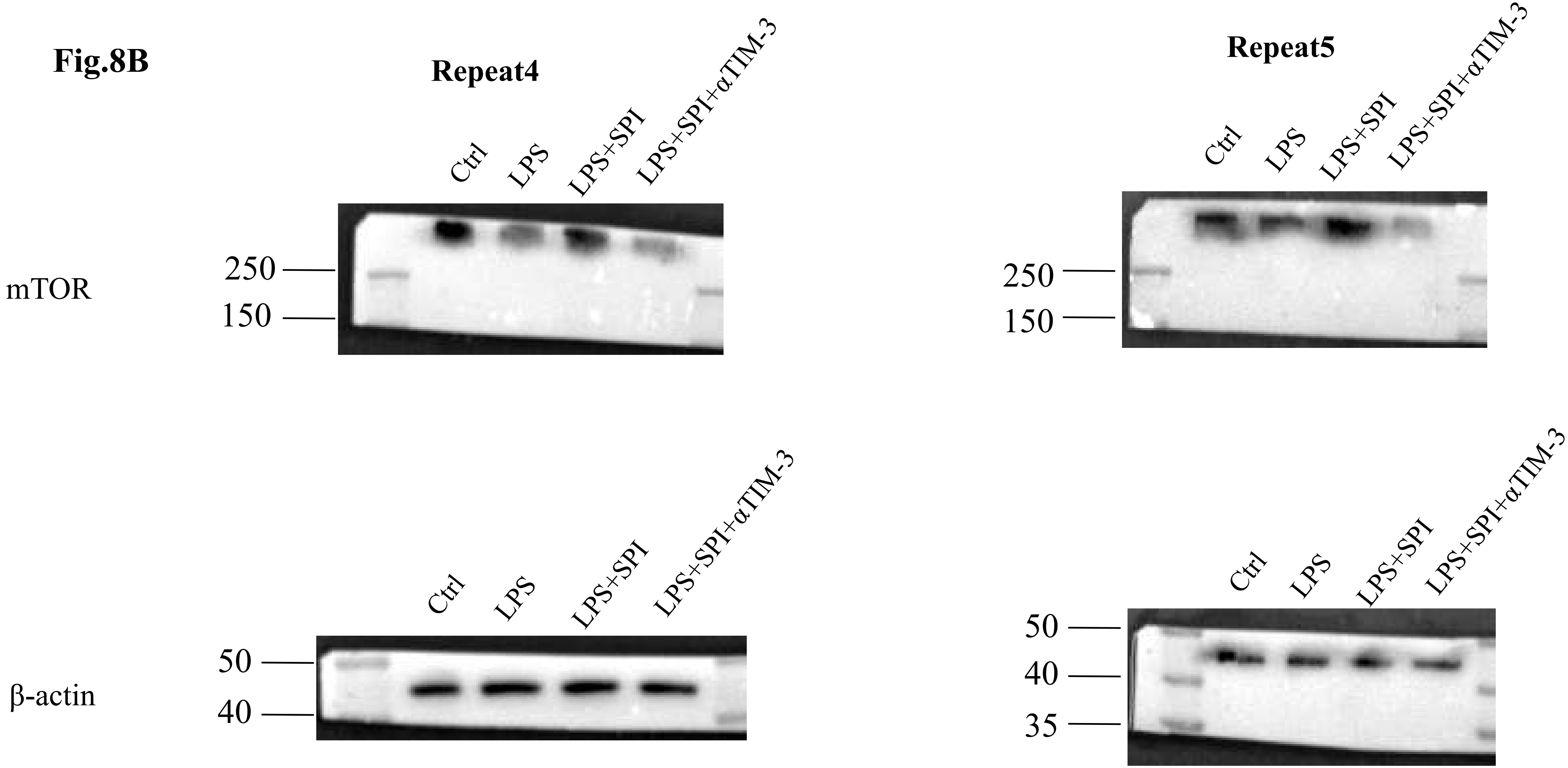
Repeat4
Repeat5
Ctrl
LPS
LPS+SPI
LPS+SPI+αTIM-3
mTOR
250
150
β-actin
50
40
35

Fig.8B

P-mTOR

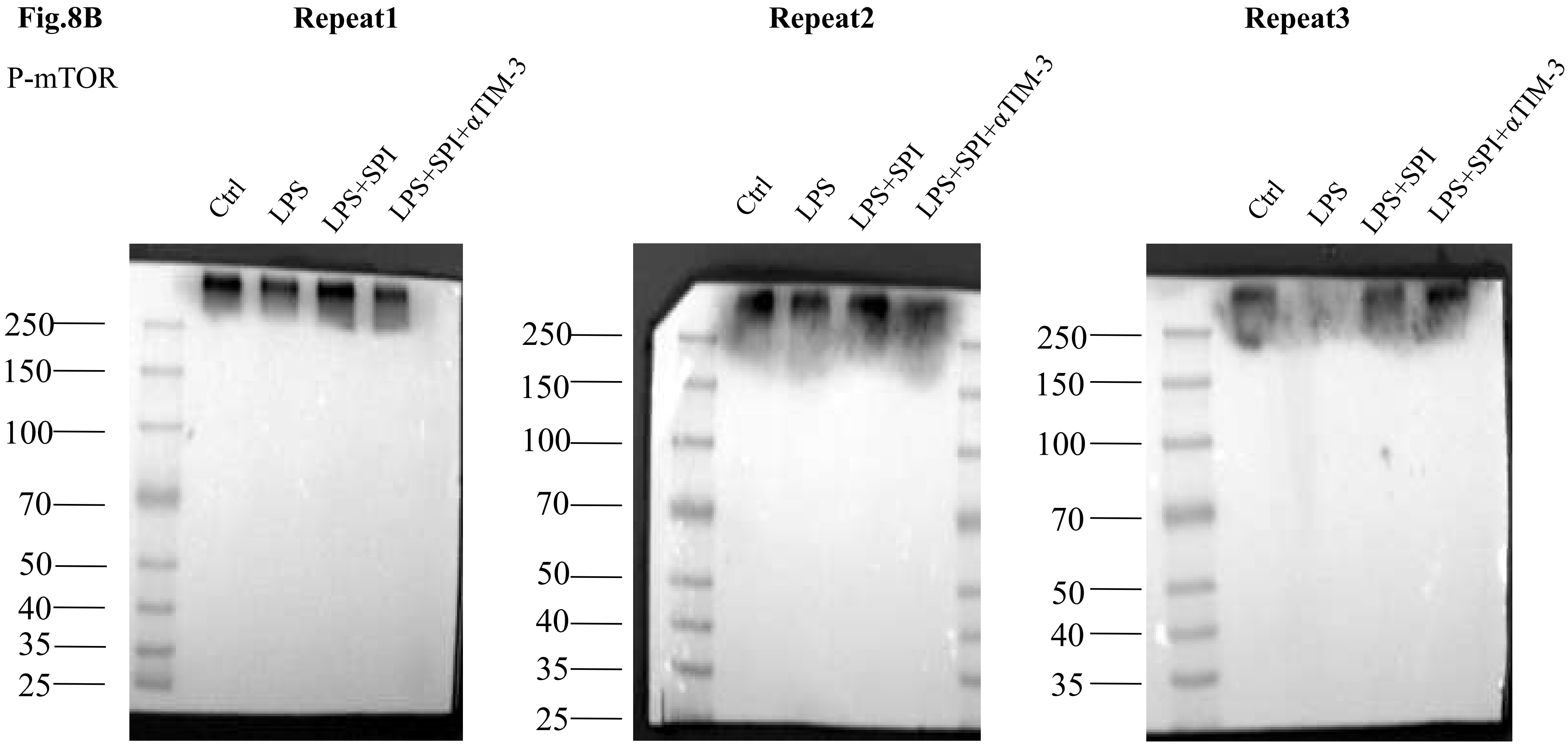
Repeat1
Repeat2
Repeat3
Ctrl
LPS
LPS+SPI
LPS+SPI+αTIM-3
250
150
100
70
50
40
35
25

Fig.8B

β-actin

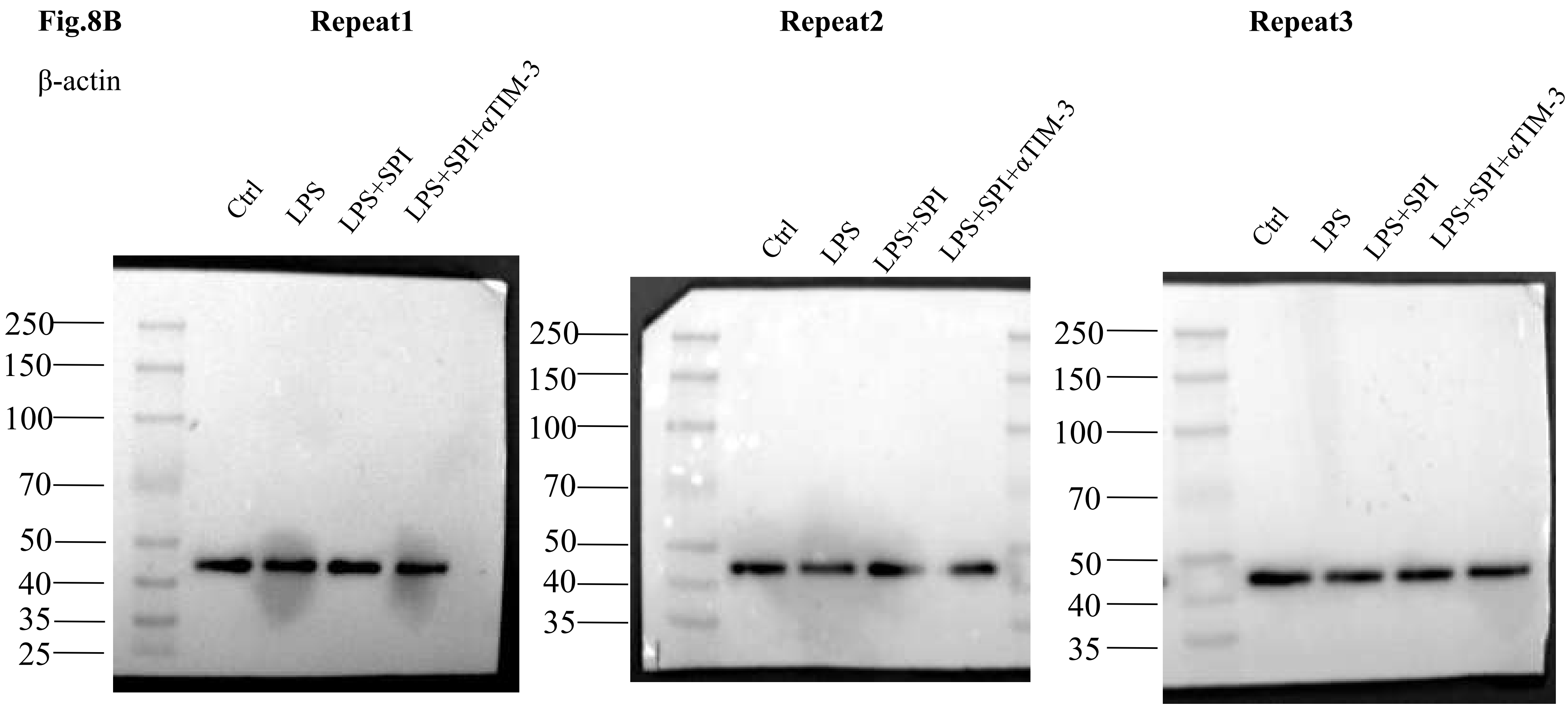

Fig.8B

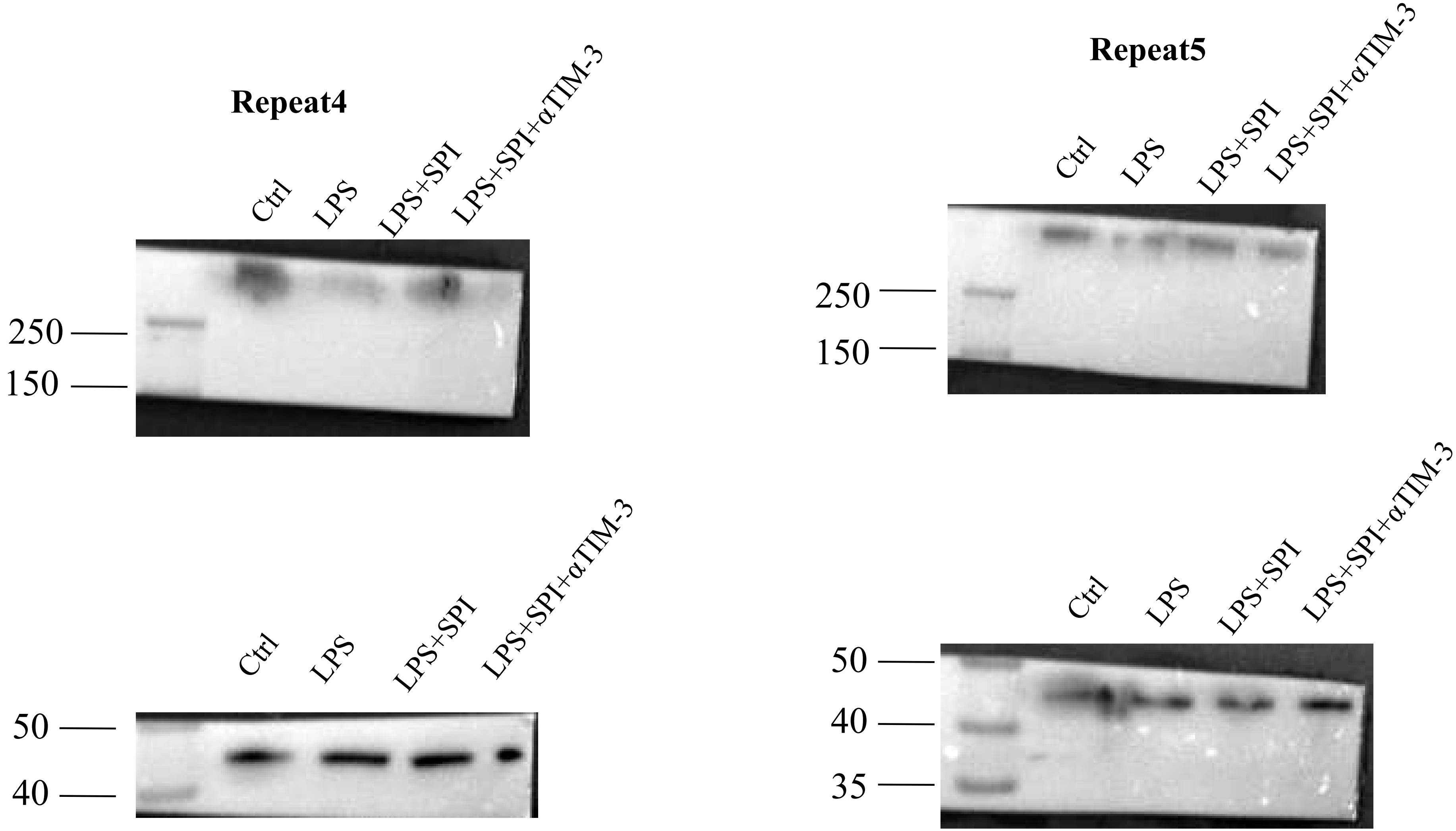

P-mTOR

β-actin

Fig.8C

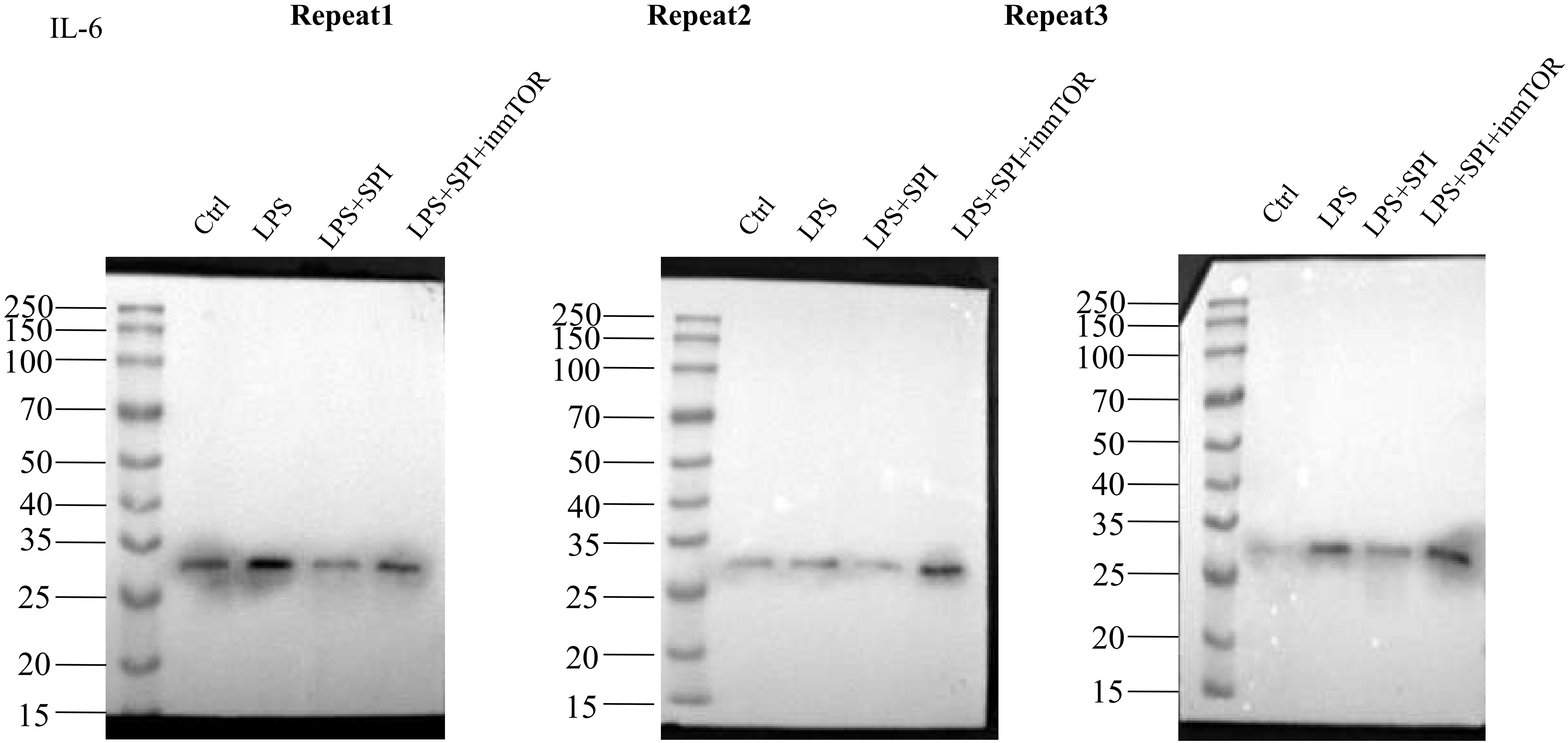
IL-6
Repeat1
Repeat2
Repeat3
Ctrl
LPS
LPS+SPI
LPS+SPI+inmTOR
250
150
100
70
50
40
35
25
20
15

Fig.8C

β-actin

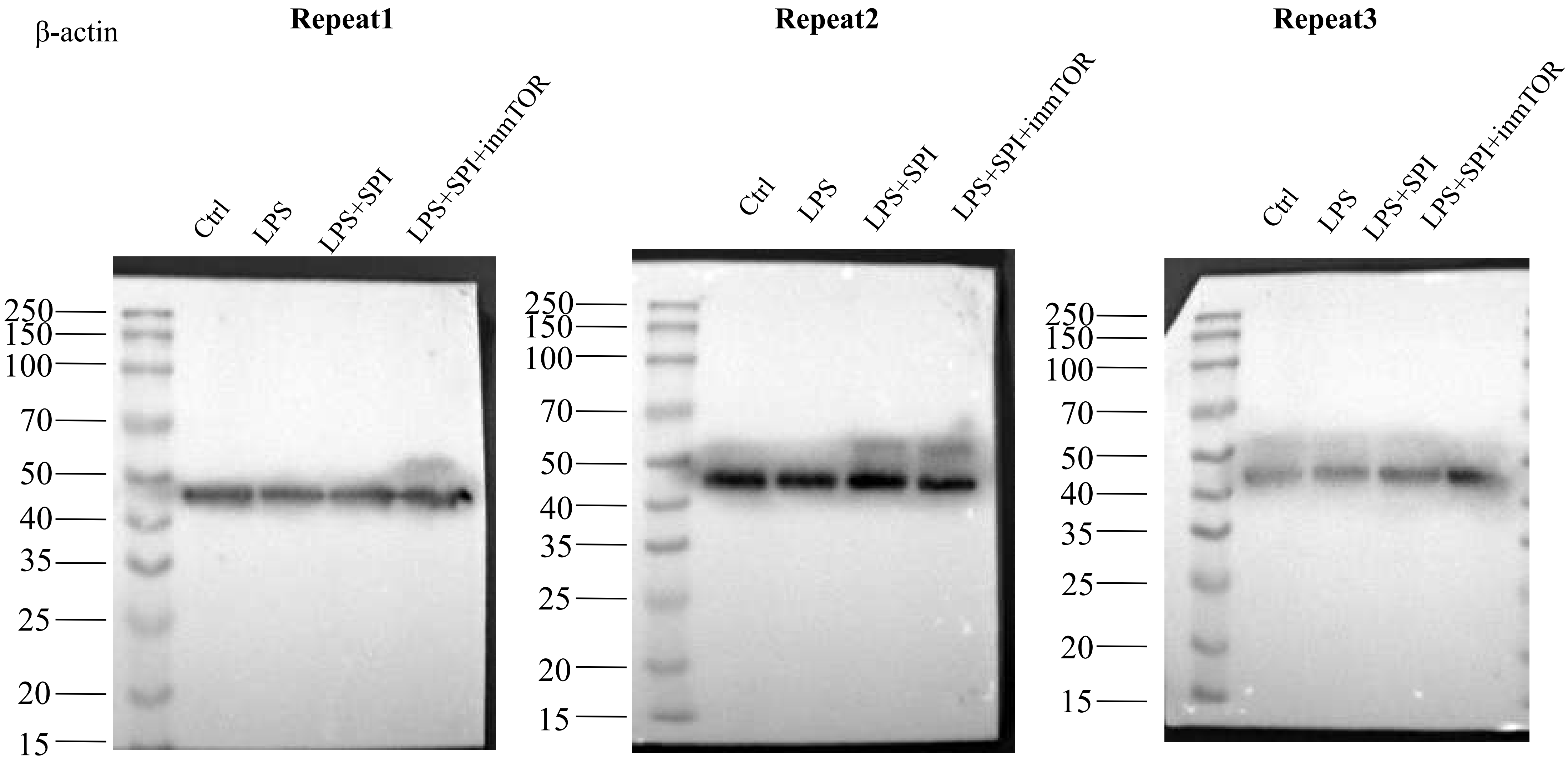

Fig.8C

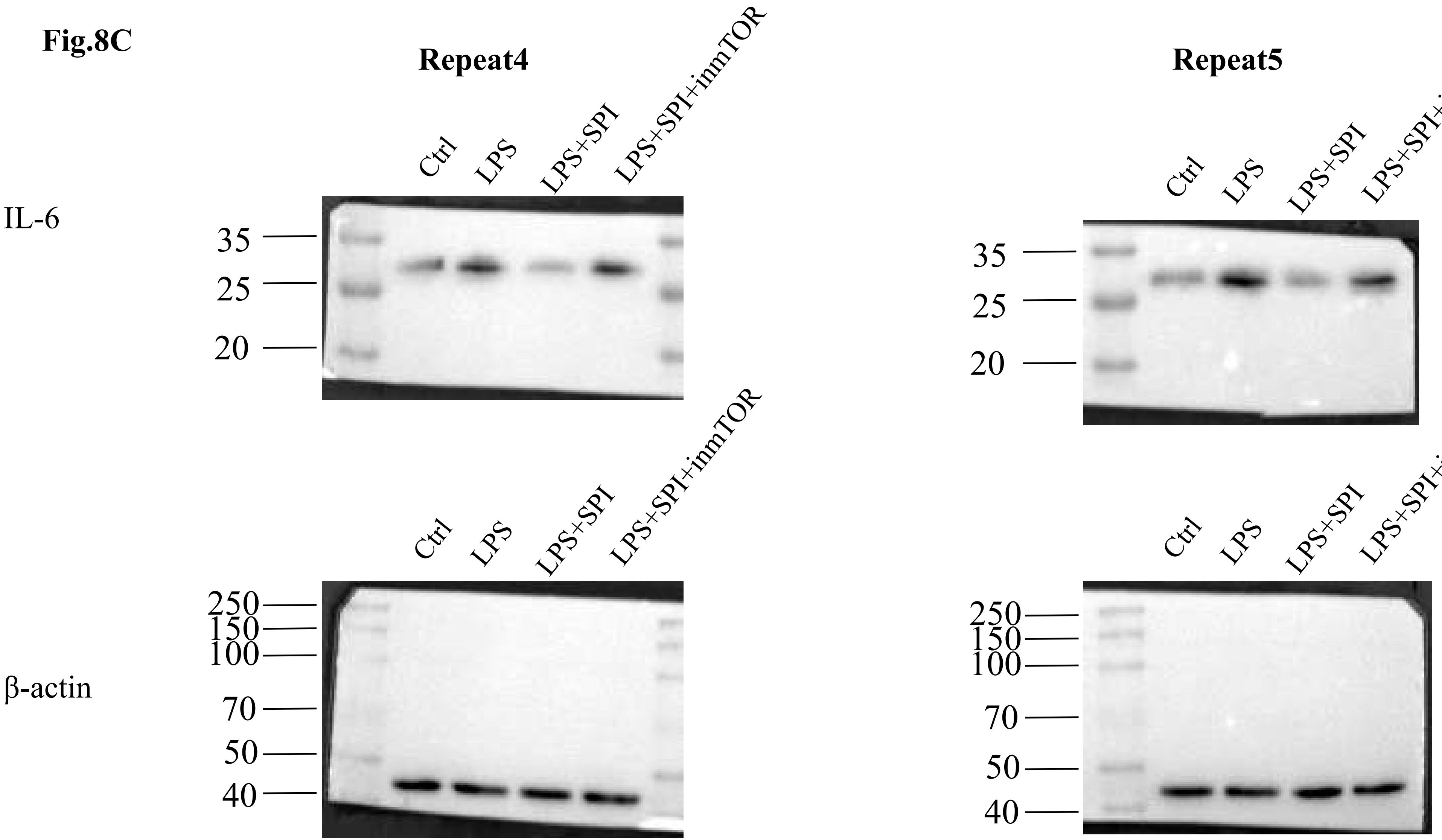
Repeat4
Repeat5
IL-6
β-actin
Ctrl
LPS
LPS+SPI
LPS+SPI+inmTOR
35
25
20
250
150
100
70
50
40

Fig.8C

mTOR

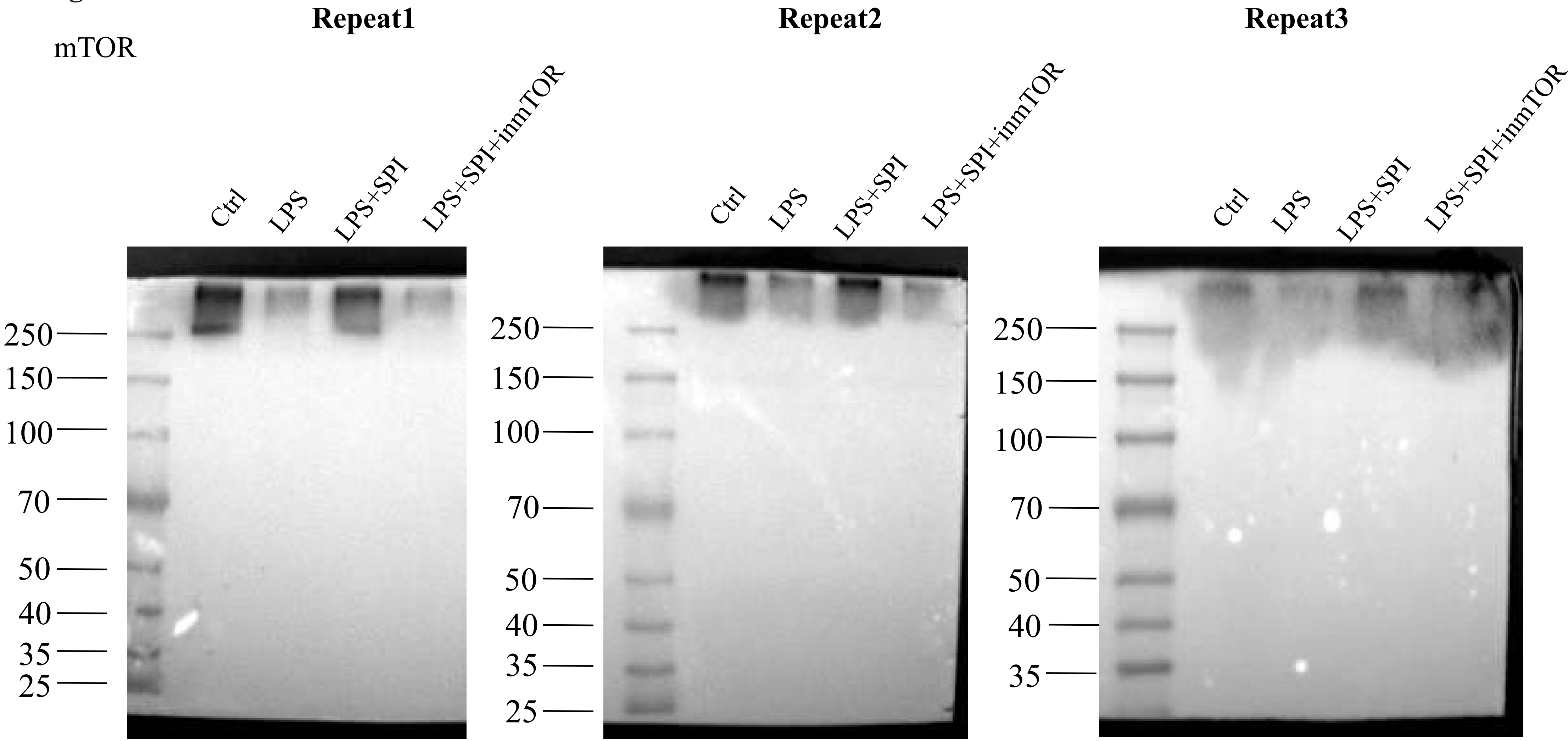

Repeat1
Repeat2
Repeat3
Ctrl
LPS
LPS+SPI
LPS+SPI+inmTOR
250
150
100
70
50
40
35
25

Fig.8C

β-actin

Repeat1 **Repeat2** **Repeat3**

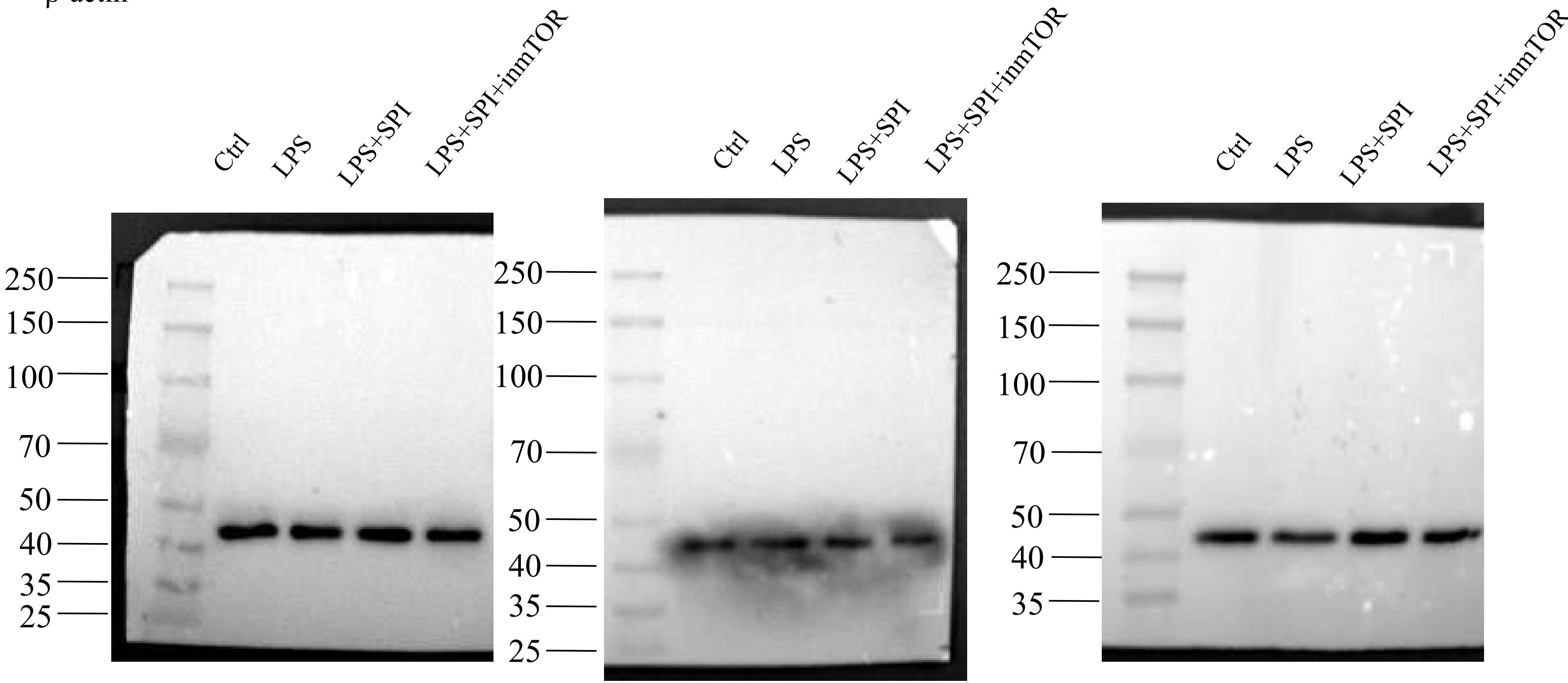

Fig.8C

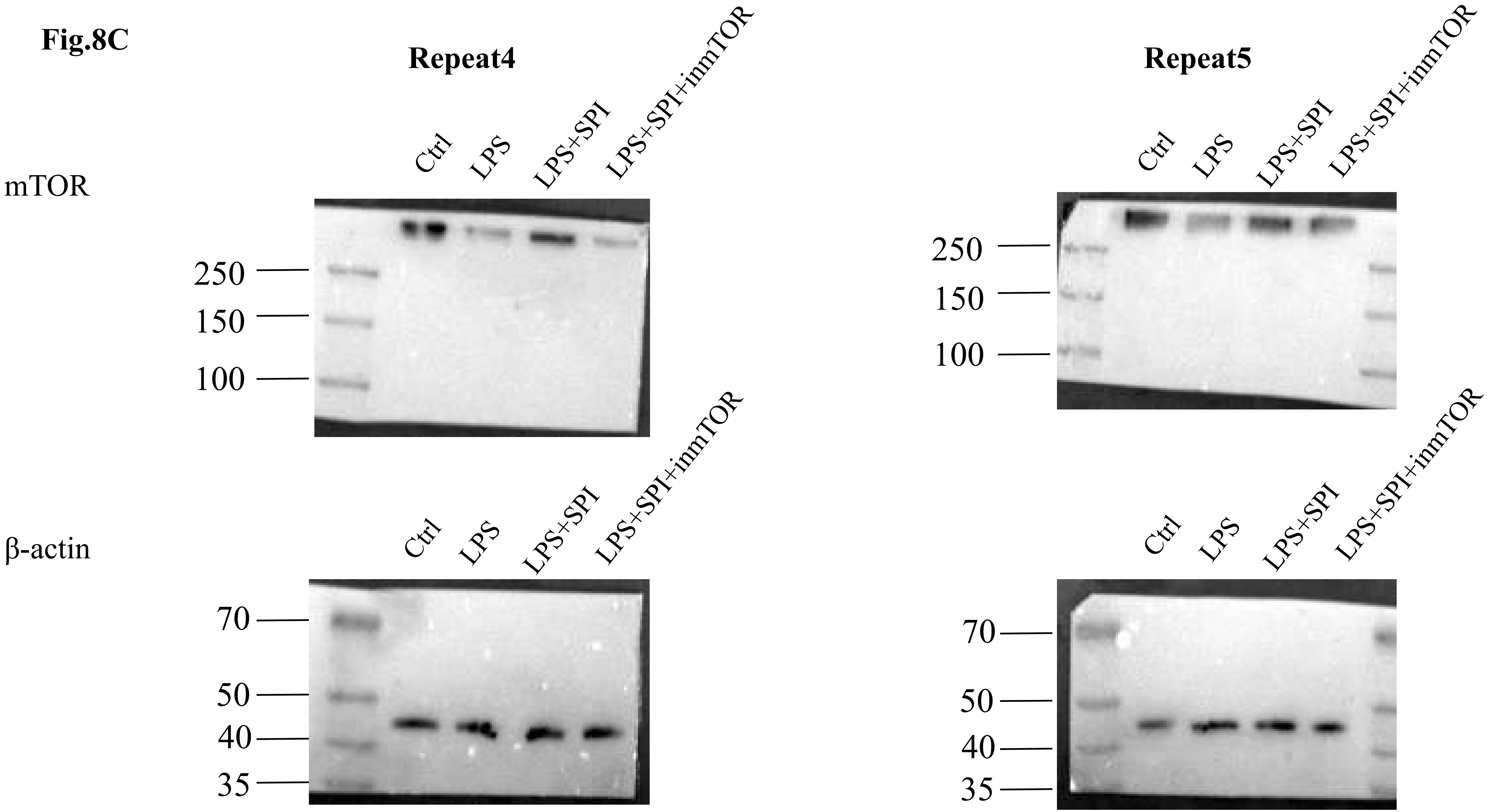
Repeat4
Repeat5
mTOR
Ctrl
LPS
LPS+SPI
LPS+SPI+inmTOR
250
150
100
β-actin
70
50
40
35
