# Supplementary material for: Serine protease inhibitor from Trichinella spiralis ameliorates diet-induced obesity and adipose tissue inflammation through TIM-3-dependent macrophage reprogramming
Source: PLoS Pathog. 2026 Sep 25;22(9):e1014606. doi: 10.1371/journal.ppat.1014606 (PMC13614578; doi:10.1371/journal.ppat.1014606)
Supplement: Supplementary file 2 [file ppat.1014606.s001.docx]

S1 Fig. Flow cytometric analysis of F4/80⁺ cells in BMDMs. Bone marrow cells were flushed from the long bones of C57BL/6J mice, seeded, and cultured in DMEM with 10% FBS and 40 ng/mL recombinant mouse (rm) M-CSF at 37°C with 5% CO_2_ for 48 h. Subsequently, all adherent cells were cultured in DMEM with 10% FBS and 40 ng/mL rm M-CSF for 72 h. Cells were then collected and analyzed for F4/80^+^ expression by flow cytometry.

S1 Table. Primer sequences used in qRT-RCR assays

| **Genes** | **Organism** | **Primer sequence (5’→3’)** | **Genebank NO.** |
| --- | --- | --- | --- |
| TNF-α | Mouse | F:GAGGTCAATCTGCCCAAGTA | NM_013693.3 |
|  |  | R:GAGCCATAATCCCCTTTCTA |  |
| IL-1β | Mouse | F:GGCTGGACTGTTTCTAATGC | NM_008361.4 |
|  |  | R:ATGGTTTCTTGTGACCCTGA |  |
| IL-6 | Mouse | F:TTCTTGGGACTGATGCTGGTG | NM_031168.2 |
|  |  | R:CACAACTCTTTTCTCATTTCCACGA |  |
| TGF-β | Mouse | F:GCCGCTTCTGCTCCCACTCC | M13177.1 |
|  |  | R:CGATGCGCTTCCGTTTCACC |  |
| IL-10 | Mouse | F:TGGACAACATACTGCTAACCGAC | NM_010548.2 |
|  |  | R:CCTGGGGCATCACTTCTACC |  |
| iNOS | Mouse | F:GGTGAAGGGACTGAGCTGTT | NM_010927.4 |
|  |  | R:ACGTTCTCCGTTCTCTTGCAG |  |
| Arg-1 | Mouse | F:CATTGGCTTGCGAGACGTAGAC | NM_007482.3 |
|  |  | R:GCTGAAGGTCTCTTCCATCACC |  |
| MCP-1 | Mouse | F:ACTGCATCTGCCCTAAGGTCTTCA | NM_011333.3 |
|  |  | R:AGAAGTGCTTGAGGTGGTTGTGGA |  |
| TIM-3 | Mouse | F:TCAGGTCTTACCCTCAACTGTG | [NM_134250.2](https://www.ncbi.nlm.nih.gov/entrez/viewer.fcgi?db=nucleotide&id=160333698) |
|  |  | R:GGCATTCTTACCAACCTCACACACA |  |
| β-actin | Mouse | F:GCTGTCCCTGTATGCCTCTG | NM_007393.5 |
|  |  | R:TTTGATGTCACGCACGATTT |  |
